# Regulation of fungal raw-starch-degrading enzyme production depends on transcription factor phosphorylation and recruitment of the Mediator complex

**DOI:** 10.1038/s42003-023-05404-x

**Published:** 2023-10-12

**Authors:** Yuan-Ni Ning, Di Tian, Man-Li Tan, Xue-Mei Luo, Shuai Zhao, Jia-Xun Feng

**Affiliations:** 1 State Key Laboratory for Conservation and Utilization of Subtropical Agro-bioresources, Guangxi University, 100 Daxue Road, Nanning, Guangxi 530004 PR China; 2 Guangxi Research Center for Microbial and Enzyme Engineering Technology, Guangxi University, 100 Daxue Road, Nanning, Guangxi 530004 PR China; 3 College of Life Science and Technology, Guangxi University, 100 Daxue Road, Nanning, Guangxi 530004 PR China

**Keywords:** Fungal genetics, Gene regulation, Applied microbiology

## Abstract

Filamentous fungus can produce raw-starch-degrading enzyme (RSDE) that efficiently degrades raw starch below starch gelatinization temperature. Employment of RSDE in starch processing can save energy. A key putative transcription factor PoxRsrA (production of **r**aw-**s**tarch-degrading enzyme **r**egulation in ***P****enicillium* ***ox****alicum*) was identified to regulate RSDE production in *P. oxalicum*; however, its regulatory mechanism remains unclear. Here we show that PoxRsrA_1434–1730_ was the transcriptional activation domain, with essential residues, D1508, W1509 and M1510. SANT (**S**WI3, **A**DA2, **N**-CoR and **T**FIIIB)-like domain 1 (SANT1) bound to DNA at the sequence 5′-RHCDDGGD-3′ in the promoter regions of genes encoding major amylases, with an essential residue, R866. SANT2 interacted with a putative 3-hydroxyisobutyryl-CoA hydrolase, which suppressed phosphorylation at tyrosines Y1127 and Y1170 of PoxRsrA_901–1360_, thereby inhibiting RSDE biosynthesis. PoxRsrA_1135–1439_ regulated mycelial sporulation by interacting with Mediator subunit Med6, whereas PoxRsrA_1440–1794_ regulated RSDE biosynthesis by binding to Med31. Overexpression of *PoxRsrA* increased sporulation and RSDE production. These findings provide insights into the regulatory mechanisms of fungal RSDE biosynthesis.

## Abstract (web summary)

Transcription factor PoxRsrA of *Penicillium oxalicum* is a nuclear protein containing two SANT domains with different functions, and regulates raw-starch-degrading enzyme production.

## Introduction

Amylases form an important part of the global enzyme market, accounting for about 25%^1^. Amylases can catalyze the hydrolysis of starch into glucose and maltose, and the main amylases are α-amylase, β-amylase and glucoamylase. Current starch processing methods involve high-temperature (80−105 °C) starch gelatinization, then liquefaction with α-amylase, followed by saccharification with glucoamylase at 60−65 °C^2,3^.

Interestingly, the recently-discovered raw-starch-degrading enzymes (RSDEs), specifically raw-starch-degrading glucoamylase (RSDG), from filamentous fungi, can digest relatively intact starch granules into glucose, below starch gelatinization temperature, suggesting that they have great potential to improve the efficiency and economics of starch processing. However, the low natural production of RSDEs by filamentous fungi severely limits their availability and is a major barrier to industrial use.

The biosynthesis of RSDEs is strictly regulated by transcription factors (TFs), such as PoxRsrA (formerly POX01907, production of RSDE **r**egulation in ***P****enicillium* ***ox****alicum*)^4^, AmyR^3^ and PoxCxrC^5^. TFs regulate transcription through binding to their target DNA sequence in the promoter region of the target gene, direct protein-protein interaction, or effect on chromatin remodeling; their functions are also controlled by transcriptional co-regulators^6–8^. However, the mechanisms of action of TFs in regulating the expression of RSDE genes are still only partially understood.

A classic TF contains a DNA-binding domain and a transcriptional activation domain (AD) that promotes transcription by recruitment of essential co-activators, including the Mediator, SAGA, and SWI/SNF^9^. The Mediator mediates the connection between specific TFs and the basal transcriptional machinery required for transcription. Previous reports mainly focused on the yeast, mammalian^8^ and *Arabidopsis*^10^ Mediators, whereas few involve filamentous fungal Mediators. The distinct functions of 11 Mediator subunits were recently elucidated in development, virulence, stress response and secondary metabolism of the fungal pathogen *Fusarium verticillioides*^11^, however, studies on Mediators in industrially useful fungi, such as *Penicillium oxalicum* have not been reported thus far.

*P. oxalicum* is widely distributed in natural habitats and produces RSDEs^12,13^. In a previous study, a TF, PoxRsrA was identified as essential for regulating the production of RSDEs, as well as expression of *PoxGA15A*, the gene encoding a key RSDG^4^. This study was to fully elucidate the molecular mechanisms of action of PoxRsrA. Phosphorylation of PoxRsrA and recruitment of Mediator by PoxRsrA were essential for its function in up-regulating expression of *PoxGA15A* and other RSDE genes, and thereby RSDE production. Overexpression of *PoxRsrA* in *P. oxalicum* significantly enhanced RSDE production.

## Results

### PoxRsrA is a nuclear protein

To investigate the subcellular localization of PoxRsrA, green fluorescent protein (GFP) was employed as a reporter. A mutant PoxRsrA::GFP was constructed by homologous recombination in a background strain, Δ*PoxKu70* (also used as the wild-type [WT] control). In the mutant, the whole of PoxRsrA was fused with reporter GFP at the C-terminus (Supplementary Fig. S1a), then the mutant was confirmed by PCR (Supplementary Fig. S1b). In addition, a quantitative PCR (qPCR) assay demonstrated that a single DNA cassette was inserted into the WT genome during the construction of mutant PoxRsrA::GFP (Supplementary Fig. S2). When cultured in medium containing soluble corn starch (SCS) for 2–4 days after transfer from glucose, the RSDE and soluble starch degrading enzyme (SSDE) production by mutant PoxRsrA::GFP were similar to those of WT (Supplementary Fig. S3a, b). The colony phenotypes and sporulation on plates containing potato dextrin agar (PDA), glucose and SCS, were also similar for the two strains (Supplementary Fig. S3c–e).

**Fig. 1:**
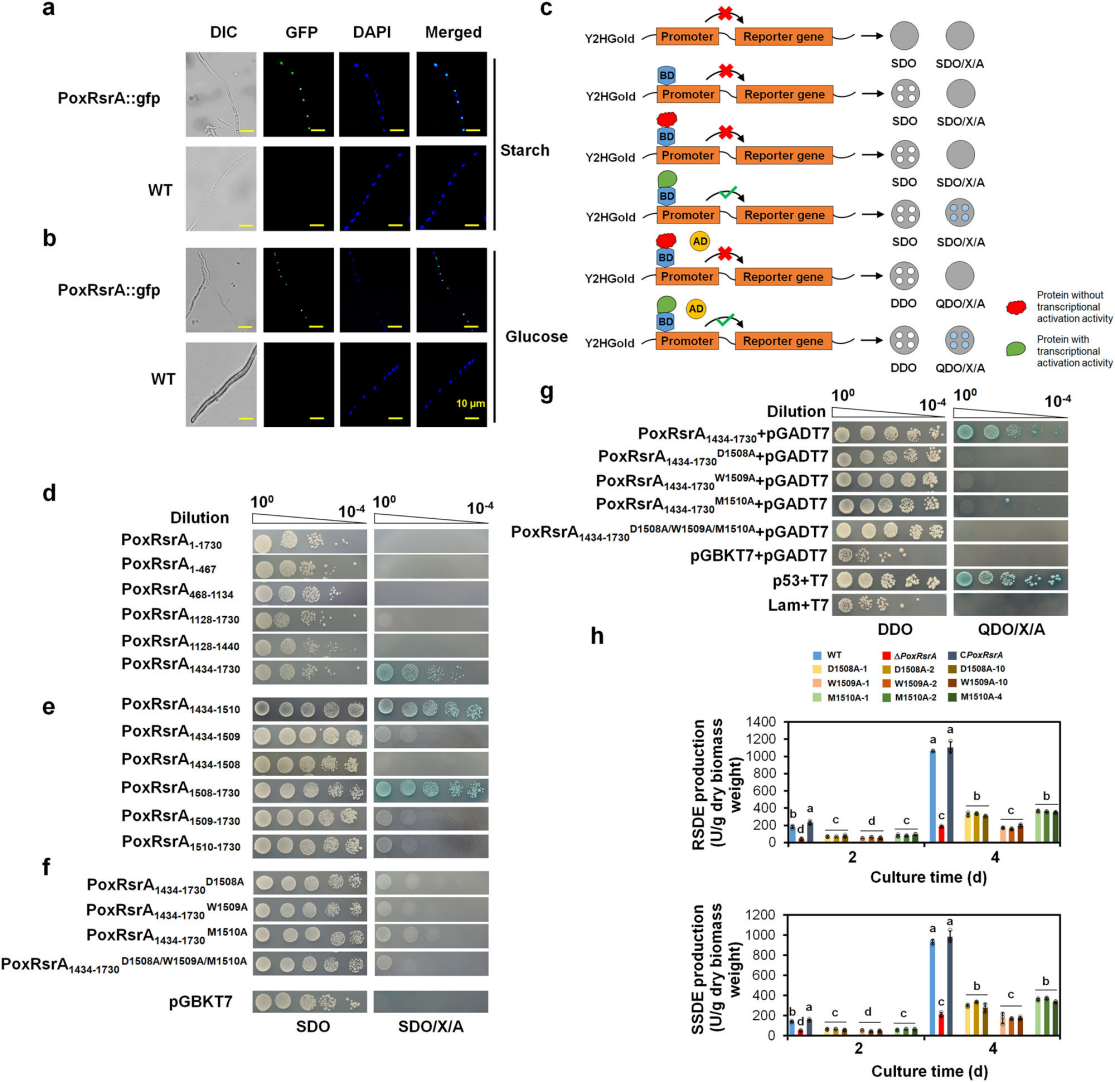
Subcellular localization of PoxRsrA and identification of transcriptional activation domain and key amino acids in PoxRsrA. **a** Images indicating the localization of PoxRsrA in *P. oxalicum* when cultivated in the presence of soluble corn starch. **b** Images indicating the localization of PoxRsrA in *P. oxalicum* when cultivated in the presence of glucose. The hyphae from *P*. *oxalicum* mutant PoxRsrA::GFP and the wild-type strain (WT) Δ*PoxKu70* cultured in medium containing soluble corn starch or glucose for 24 h, respectively, were observed by fluorescence microscopy. The expression of *PoxRsrA-gfp* was controlled by the natural promoter of *PoxRsrA*. 4,6-Diamidino-2-phenylindole (DAPI) dye was used for specific nuclear staining. GFP: green fluorescent protein. DIC: differential interference contrast. **c** Schematic illustration showing principle of yeast autoactivation assay. BD: DNA-binding domain. AD: transcriptional activation domain. SDO: synthetic dropout medium (SD) without tryptophan (SD/-Trp). SDO/X/A: SD-Trp/+X-α-Gal/+Aureobasidin A. DDO: SD-Trp/-Leu. QDO/X/A: SD-Trp/-Leu/-Ade/-His/+X-α-Gal/+Aureobasidin A. **d** Identification of AD in PoxRsrA. **e**–**g** Identification of the key amino acids for transcription activation. Yeast cells carrying different length of PoxRsrA polypeptides, or site-mutated polypeptides were diluted by a tenfold gradient and then cultured on SDO, SDO/X/A, DDO, or QDO/X/A plates for four days. The original concentration of each yeast sample was adjusted to OD_600_ = 1.0. **h** Effects of the key amino acids on RSDE and SSDE production in *P. oxalicum*. Each mutant included three independent transformants. Fungal strains were pre-grown in glucose medium for 24 h, then transferred into medium containing soluble corn starch and cultured for 2–4 days. Data values are mean ± standard deviation. All experiments were performed at least three times. Values marked with different lower case letters indicate statistically significant differences (*p* < 0.05) between each group according to one-way ANOVA. RSDE raw-starch-degrading enzyme; SSDE soluble-starch-degrading enzyme.

After culture in medium containing SCS, or glucose for 24 h, the mycelia of both mutant PoxRsrA::GFP and WT were harvested. Observation by fluorescence microscopy revealed that the green fluorescence was observed as discontinuous spots in the hyphae of mutant PoxRsrA::GFP and overlapped the blue fluorescent signal from 4,6-diamidino-2-phenylindole, a specific nuclear stain. No green fluorescence signal was detected in WT hyphae. These results indicate that PoxRsrA is translocated to the nucleus in the presence of SCS and glucose (Fig. 1a and b).

### PoxRsrA_1434–1730_ identified as the transcriptional activation domain

An autoactivation experiment in yeast Y2HGold cells was carried out to locate the AD of PoxRsrA. Y2HGold cells can grow well and turn blue on SDO/X/A (SD/-Trp/+X-α-Gal/+ Aureobasidin A) plates, only when harboring a protein with transcriptional activation activity (Fig. 1c). The sequence of PoxRsrA_1–1730_ was divided into fragments of various lengths (Fig. 1d), the coding DNA fragments of these polypeptides were cloned into the plasmid pGBKT7, then each recombinant plasmid was introduced into yeast Y2HGold cells. Recombinant Y2HGold/pGBKT7-*PoxRsrA*_1434–1730_ grew normally on SDO (SD/-Trp) and SDO/X/A plates, whereas the other recombinants could only grow on SDO plates. Moreover, Y2HGold/pGBKT7-*PoxRsrA*_1434–1730_ colonies turned blue on SDO/X/A plates (Fig. 1d), indicating that PoxRsrA_1434–1730_ has transcriptional activation activity and is the location of the AD.

### Residues D1508, W1509 and M1510 of PoxRsrA are required for production of RSDE

To identify the essential residues required for transcriptional activation, DNA sequence encoding PoxRsrA_1434–1730_ was progressively truncated from either end, to generate polypeptides of different lengths (Supplementary Fig. S4a). The DNA fragments encoding these polypeptides were separately cloned into the plasmid pGBKT7, then each recombinant plasmid was introduced into yeast Y2HGold cells. Most of the resulting yeast transformants grew well on SDO plates and on SDO/X/A plates, with blue colonies. Notably, 16 yeast transformants as shown in Fig. 1e and Supplementary Fig. S4, for example, Y2H/PoxRsrA_1434–1508_, Y2H/PoxRsrA_1434–1509_, Y2H/PoxRsrA_1509–1730_, and Y2H/PoxRsrA_1510–1730_, grew very poorly on SDO/X/A plates, but grew well on SDO plates. Notably, yeast cells carrying PoxRsrA_1434-1508_ at high concentration were unable to grow at all on SDO/X/A plates, whereas cells carrying PoxRsrA_1434-1509_, at high concentration, could grow on SDO/X/A plates (Fig. 1e and Supplementary Fig. S4b). These results indicate that the residues D1508, W1509 and M1510 are essential for transcriptional activation of PoxRsrA.

In addition, these conserved residues were site-mutated to A, to generate PoxRsrA_1434-1730_^D1508A^, PoxRsrA_1434-1730_^W1509A^, PoxRsrA_1434-1730_^M1510A^ and PoxRsrA_1434-1730_^D1508A/W1509A/M1510A^. Yeast autoactivation experiments revealed that yeast transformants carrying these mutated polypeptides were unable to grow on SDO/X/A, but grew normally on SDO (Fig. 1f). In addition, the yeast transformants carrying both site-mutated PoxRsrA_1434-1730_ and plasmid pGADT7 could not grow on QDO/X/A (SD/-Trp/-Leu/-His/-Ade/+X-α-Gal/+Aureobasidin A), but grew normally on DDO (SD/-Trp/-Leu), while the transformant carrying PoxRsrA_1434-1730_ and pGADT7 grew well on both DDO and QDO/X/A (Fig. 1g). Therefore, the site-mutated polypeptide PoxRsrA_1434-1730_ almost completely lost its transcriptional activation activity, confirming that residues D1508, W1509 and M1510 are essential for functioning of the AD.

To verify the effects of these three residues on the biosynthesis of amylase and sporulation in *P. oxalicum*, the DNA sequences encoding site-mutated PoxRsrA^D1508A^, PoxRsrA^W1509A^ and PoxRsrA^M1510A^ were introduced into mutant Δ*PoxRsrA* at the *POX_d05452* site, to obtain the strains D1508A, W1509A and M1510A, respectively (Supplementary Fig. S5). The *POX_d05452* site was used because no mutant was obtained when the DNA cassette was inserted into the original *PoxRsrA* locus in ∆*PoxRsrA*. *POX_d05452* encodes an aspartic protease, PepA; its deletion resulted in a phenotype indistinguishable from WT, including the same production levels of RSDE and SSDE^14^. qPCR determined that a single DNA cassette was inserted into the genome of Δ*PoxRsrA* (Supplementary Fig. S2).

Enzyme assays to determine enzyme production revealed that the RSDE and SSDE production of W1509A was similar to that of the deletion mutant Δ*PoxRsrA*, whereas both D1508A and M1510A had higher RSDE and SSDE production than Δ*PoxRsrA*, but lower than WT and the complementation strain, C*PoxRsrA*, after 2–4 days of induction by SCS. These data indicate that D1508, W1509 and M1510 are essential for RSDE and SSDE biosynthesis and that W1509 is the most important (Fig. 1h).

### SANT1 is responsible for DNA-binding

**Fig. 2:**
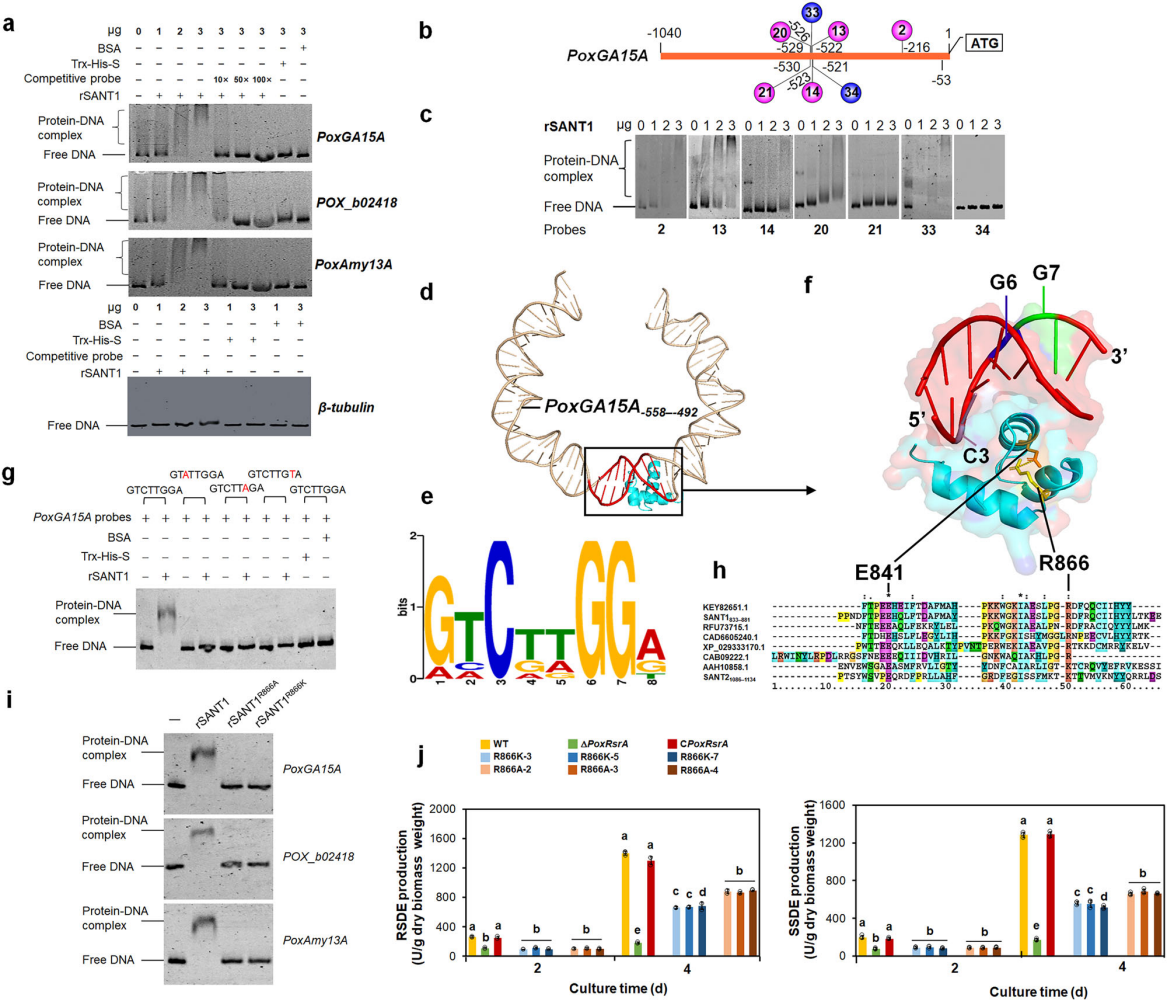
Identification of DNA sequence bound by SANT1 and key amino acids for DNA binding. **a** In vitro electrophoretic mobility shift assay (EMSA) between the recombinant protein rSANT1 and the promoter regions of major amylase genes. Each reaction contained Trx-His-S-tagged rSANT1 (0–3 μg) and 6-carboxyfluorescein-tagged probe (~50 ng). The Trx-His-S peptide and bovine serum albumen (BSA) were used as controls, as well as the promoter region of the β-tubulin gene. Competitive probes were DNA fragments without 6-carboxyfluorescein. *PoxGA15A*: raw-starch-degrading glucoamylase gene. *POX_b02418*: glucoamylase gene. *PoxAmy13A*: α-amylase gene. **b** Schematic diagram indicating the truncated *PoxGA15A* probes for EMSA. Probe 2: *PoxGA15A*_-1040–-216_, 13: *PoxGA15A*_-1040–-522_, 14: *PoxGA15A*_-1040–-523_, 20: *PoxGA15A*_-1040–-529_, 21: *PoxGA15A*_-1040–-530_, 33: *PoxGA15A*_-526–-53_, 34: *PoxGA15A*_-521–-53_. **c** EMSA between rSANT1 and the truncated probes shown in panel (**b**). **d** Auto-docking analysis between SANT1 motif and DNA fragment *PoxGA15A*_*-558–492*_. The three-dimensional structural model of SANT1 was constructed, based on the homologous HDAC3 (pdb ID: 4A69) via SWISS-MODEL. The 3dRNA/DNA Web Server was employed to construct the model of *PoxGA15A*_-558–-492_. Molecular docking of SANT1 with *PoxGA15A*_-558–-492_ was carried out by the HDOCK SERVER, then visualized by PyMOL software. In the rectangle, red and blue colors represent DNA fragment *PoxGA15A*_-529–-522_ (5’-GTCTTGGA-3’) and the SANT1 motif, respectively. **e** MEME-predicted conserved DNA sequence bound by SANT1. **f** Enlarged view marked with rectangle in panel d. C3, G6 and G7 represent the conserved C, G and G at 3rd, 6th and 7th positions in the motif as shown in panel (**e**). **g** EMSA indicating the binding of mutant probes by rSANT1 (3 μg). The mutated *PoxGA15A* probe has “A” or “T” instead of C, G and G at 3rd, 6th and 7th positions as shown in panel (**e**), respectively. **h** Sequence alignment analysis of SANT1, SANT2 and their homologs. “*” indicates the identical amino acids in all aligned sequences. “:” and “.” indicate conserved amino acids with very and moderately similar properties, respectively. **i** EMSA between the mutated SANT1 (3 μg) and probes from genes encoding major amylases. In the mutated SANT1, R866 was exchanged for A or K. “–” indicates no protein added. **j** Effects of R866 on the production of RSDE and SSDE in *P. oxalicum*. The site-mutants R866A and R866K, deletion mutant Δ*PoxRsrA*, complementation strain C*PoxRsrA* and wild-type strain (WT) were cultured in medium containing soluble corn starch for 2–4 days after transfer from glucose. Each mutant included three independent transformants. Data values are mean ± standard deviation. All experiments were performed at least three times. Values marked with different lower case letters indicate statistically significant differences (*p* < 0.05) between each group according to one-way ANOVA. RSDE raw starch degrading enzyme; SSDE soluble starch degrading enzyme.

Simple Modular Architecture Research Tool (SMART) analysis showed that PoxRsrA contained two SANT (**S**witching-defective protein 3 [Swi3], **A**daptor 2 [Ada2], **N**uclear receptor corepressor [N-CoR] and general TF [**T**FIIIB])-like domains but with different sequences, namely SANT1 (residues 830–883) and SANT2 (residues 1080–1140). An in vitro electrophoretic mobility shift assay (EMSA), using 6-carboxyfluorescein (6-FAM) labeling, showed clear shift bands when either recombinant rSANT1, or rSANT1-SANT2 from *Escherichia coli* was mixed with test probes of *PoxGA15A*, glucoamylase gene *POX_b02418* and α-amylase gene *PoxAmy13A*, with lengths of 988, 1036 and 864 bp upstream of the start code ATG (Supplementary Fig. S6a), but no shift band was seen with rSANT2 (Supplementary Fig. S6b). The shift bands became faint, or disappeared when competitive probes without 6-FAM were loaded; neither the negative control β-tubulin gene promoter probe (length 1002 bp), nor protein controls BSA and Tris-His-S resulted in detectable interaction with the tested protein or probes (Fig. 2a and Supplementary Fig. S6a), suggesting that rSANT1 specifically binds to the promoter regions of the three tested amylase genes.

### Identification of the core-DNA sequence bound by SANT1

To identify DNA sequences bound by rSANT1, a set of truncated *PoxGA15A* probes was designed and constructed for an EMSA through nested deletions, namely probes 1–39 (Fig. 2b and Supplementary Fig. S7a). Probes 1–20 were bound by rSANT1, whereas there was no binding between rSANT1 and probes 21–25. Similarly, when the 3′-terminus of *PoxGA15A*_-1040–-53_ was fixed and the other end was successively truncated, all the truncated probes were bound by rSANT1, except 34–39 (Fig. 2c and Supplementary Fig. S7b). It therefore appears that the core-DNA sequence bound by rSANT1 is *PoxGA15A*_-529–-522_ (5’-GTCTTGGA-3’).

Protein-DNA docking (Fig. 2d) and analysis by the MEME Suite (https://meme-suite.org/meme/), with the promoter regions from *PoxGA15A*, *POX_b02418* and *PoxAmy13A*, identified the core DNA-binding sequence 5′-RHCDDGGD-3′ (R: G/A; H: T/C/A; D: T/G/A) (Fig. 2e). Three conserved nucleotides C3, G6 and G7 were localized at the binding site between rSANT1 and DNA (Fig. 2f). When they were mutated to A, A and T, respectively, in vitro EMSA showed no shift band, when loading the mutated probes and the recombinant rSANT1 (Fig. 2g), suggesting that these residues are essential for the target sequence to be bound by SANT1.

### Residue R866 of SANT1 is required for binding to target DNA

Protein sequence alignment found two conserved residues, E841 and I859 in all SANT-like domains analyzed. R866 was found to be highly conserved among SANT1 and SANT-like domains of other origins (Fig. 2h). Molecular structure analysis indicated that R866 may form a salt bridge with E841 (Fig. 2f). However, the corresponding residue in SANT2 was K (1119), not R (Fig. 2h).

When R866 of SANT1 was site-mutated to A and K, respectively, the resulting mutants, rSANT1^R866A^ and rSANT1^R866K^ lost the ability to bind probes of *PoxGA15A*, *POX_b02418* and *PoxAmy13A* (Fig. 2i), indicating that R866 is essential for SANT1 to bind to DNA.

DNA sequences encoding mutated PoxRsrA with R866 site-mutated to A or K, were introduced into the mutant Δ*PoxRsrA*^4^, to generate mutants R866A and R866K (Supplementary Fig. S8). qPCR determined that a single DNA cassette was inserted into the genome of Δ*PoxRsrA* (Supplementary Fig. S2). RSDE and SSDE production by mutants R866K and R866A was markedly higher than that of Δ*PoxRsrA*, but lower than that of the complementation strain C*PoxRsrA*, after 4 days of induction with SCS, but comparable with that of Δ*PoxRsrA* after 2 days. Notably, RSDE and SSDE production by R866A was slightly higher than that of R866K on day 4 (Fig. 2j).

### Distinct regions of PoxRsrA have various functions

**Fig. 3:**
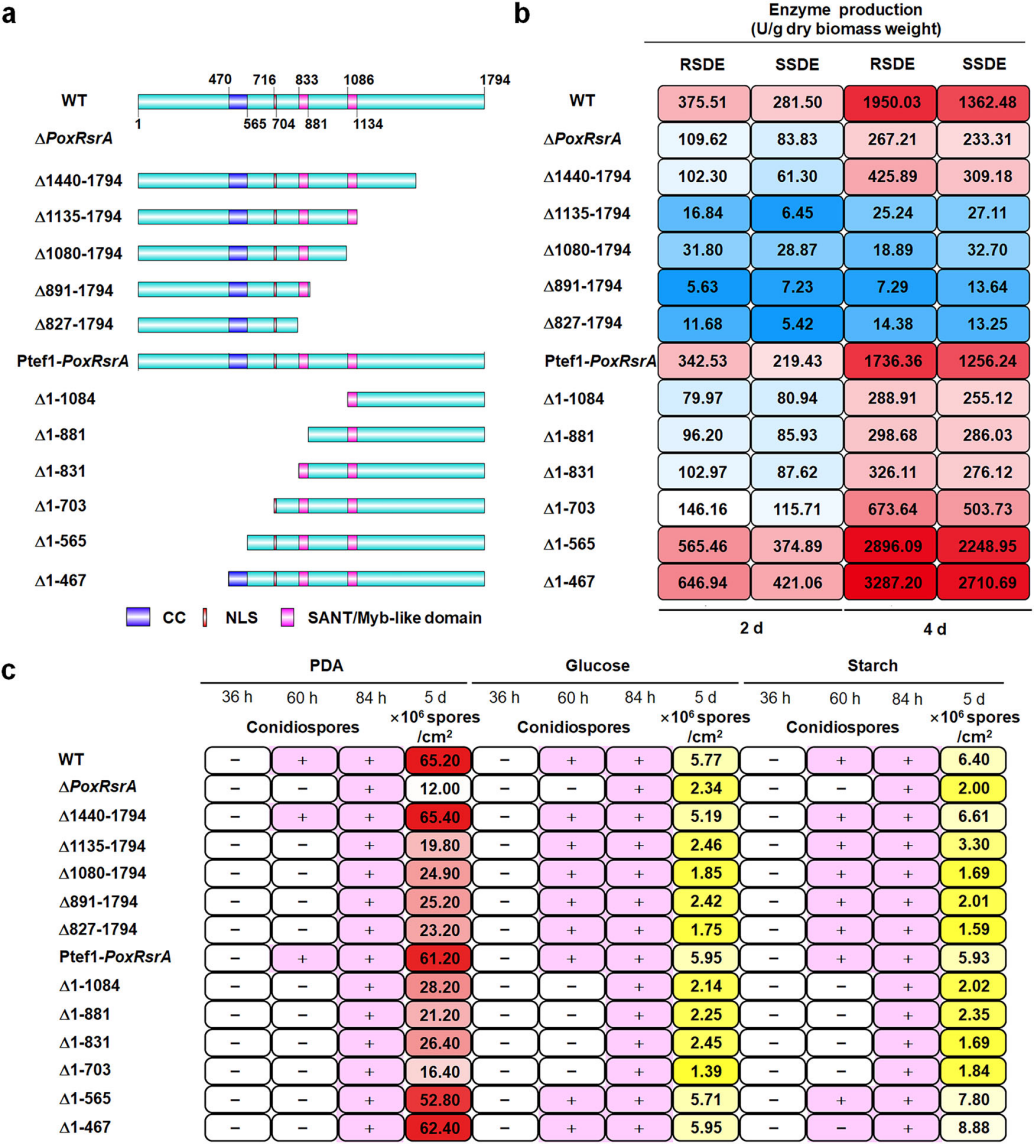
Functional analysis of distinct regions in PoxRsrA. **a** Schematic diagram indicating truncation of PoxRsrA. CC: coiled coil region. NLS: nuclear localization sequence. SANT: **S**WI3, **A**DA2, **N**-CoR and **T**FIIIB DNA-binding domain. **b** Heat mapping indicating the production of RSDE and SSDE and sporulation (**c**) by mutants carrying different truncated peptides described in panel A. In (**b**), fungal strains shown in the panel a were pre-cultured in glucose medium for 24 h, then transferred into medium containing SCS and cultured for 2–4 days. Data expressed as mean ± standard deviation. In (**c**), fungal mycelia were harvested and investigated when cultured on solid plates containing different carbon sources for 36–84 h, respectively, and spore production was determined after 5 days. PDA potato dextrin agar. WT the wild-type strain. ‘+’ and ‘-’ show conidiospore formation or not, respectively. All experiments were performed at least three times.

To investigate the functions of different domains in PoxRsrA that are involved in the biosynthesis of RSDEs and SSDEs, various DNA sequences encoding truncated PoxRsrA polypeptides were used to replace the full-length PoxRsrA (Fig. 3a) in a WT background (Supplementary Fig. S9). qPCR determined that a single DNA cassette was inserted into the WT genome (Supplementary Fig. S2). After culture in medium containing SCS for 2–4 days, mutants Δ1135–1794, Δ1080–1794, Δ891–1794, and Δ827–1794 produced negligible amounts of RSDE and SSDE, whereas Δ1440–1794 produced 72.8–78.2% less RSDE and SSDE than WT (Fig. 3b). In addition, mutant Δ1731–1794::*gfp* was constructed in the WT strain, in which PoxRsrA_1731–1794_ was replaced by GFP (Supplementary Fig. S1a), and confirmed by PCR (Supplementary Figs. S1c and S2). When exposed to SCS for 2–4 days, the RSDE and SSDE production by Δ1731–1794::*gfp* was similar to that of WT (Supplementary Figs. S10a and b). These data suggest that PoxRsrA_1135–1730_ has an important function in regulation of RSDE and SSDE production by PoxRsrA in *P. oxalicum*.

To investigate the functions of the N-terminal polypeptide of PoxRsrA, six mutants Δ1–1084, Δ1–881, Δ1–831, Δ1–703, Δ1–565 and Δ1–467 were constructed, then confirmed by PCR. *PoxRsrA* lacking one of these sequences encoding different lengths of N-terminal polypeptide was controlled by a constitutive promoter, Ptef1 (Supplementary Fig. S9). In addition, the control strain Ptef1*-PoxRsrA*, in which the native promotor of *PoxRsrA* was replaced by Ptef1, was constructed (Supplementary Fig. S9). Comparison of RSDE and SSDE production by these mutants found that enzyme production by Δ1–1084, Δ1–881 and Δ1–831 was 60.1–83.4% lower than the Ptef1*-PoxRsrA* control, whereas the mutant Δ1–703 decreased by 47.3–61.2%, when cultured in SCS medium for 2–4 days after transfer from glucose. Notably, the RSDE and SSDE production of mutants Δ1–565 and Δ1–467 was enhanced by 65.1–79.0% and 88.9–115.8%, respectively. This suggests that residues 1–467 are involved in down-regulation of RSDE and SSDE production, whereas residues 468–703 (carrying a coiled-coil region) are involved in up-regulating enzyme production (Fig. 3b).

In addition, distinct regions of PoxRsrA influenced the colony phenotypes and mycelial development of *P. oxalicum*, after culture on different carbon sources for 5 days, as shown in Supplementary Figs. S11 and S12.

Quantification of asexual spores revealed that spore production by mutant Δ1440–1794 was similar to that of WT, whereas those of Δ1135–1794, Δ1080–1794, Δ891–1794 and Δ827–1794 markedly decreased on glucose, starch or PDA; the last four mutants had similar spore production. Moreover, on glucose or starch, mutant Δ1–703 had sharply reduced spore production, compared with Δ1–565 and Δ1–467 (Fig. 3c), indicating that residues 1135–1439 and 566-703 functioned to up-regulate sporulation on starch, glucose and/or PDA.

**Fig. 4:**
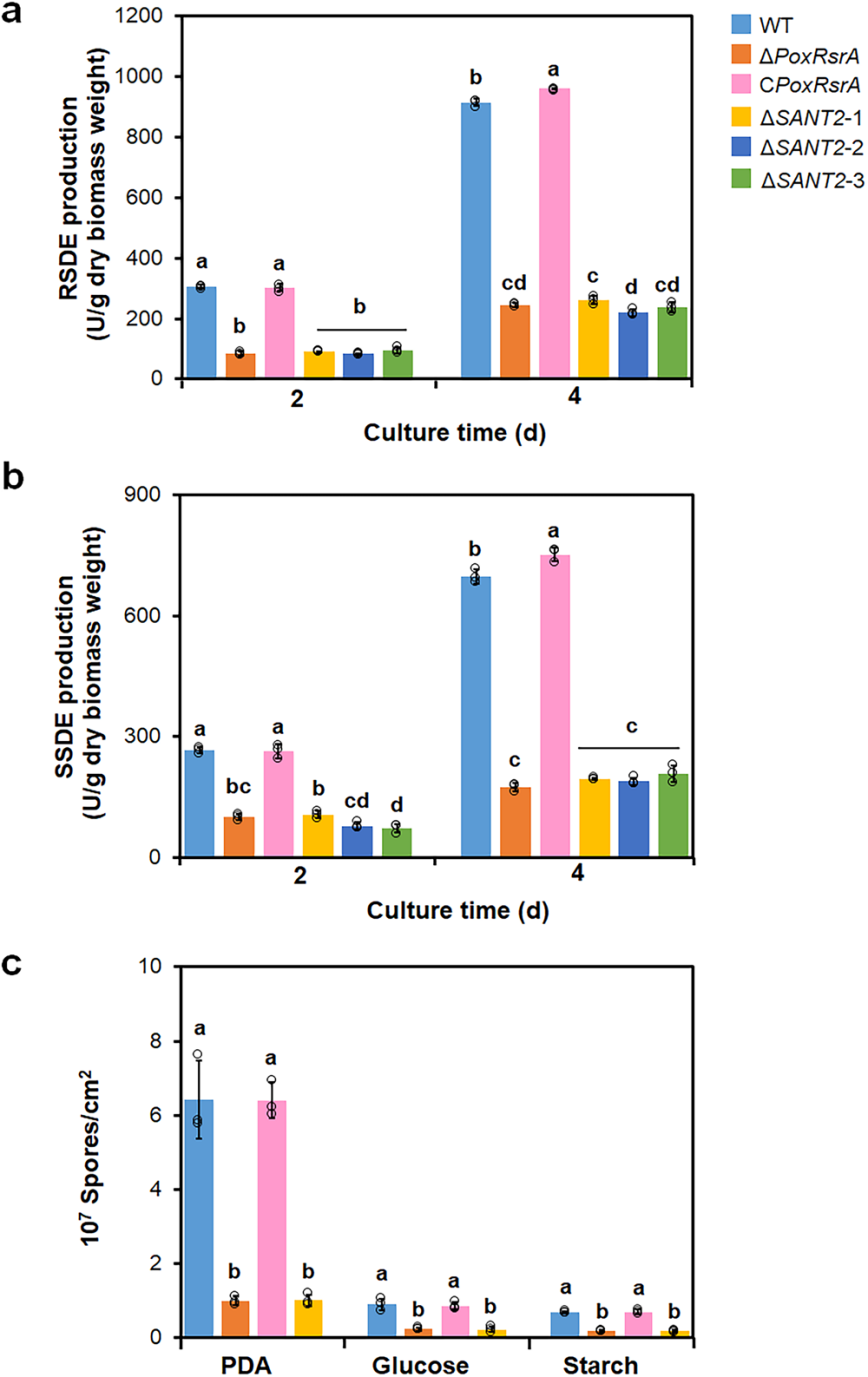
Effects of SANT2 domain in PoxRsrA on production of amylases and spores by *P. oxalicum*. **a** RSDE production. **b** SSDE production. Fungal strains were pre-cultured in glucose medium for 24 h, then transferred into medium containing soluble corn starch and cultured for 2–4 days. **c** Spore production. Fungal cells were cultured on solid plates containing different carbon sources for 5 days, respectively. Δ*SANT2*-1, Δ*SANT2*-2 and Δ*SANT2*-3 as three different transformants, were generated by introducing a DNA fragment encoding PoxRsrA, but lacking SANT2, into the deletion mutant Δ*PoxRsrA*. WT wild-type strain. PDA potato dextrin agar. Values marked with different lower case letters indicate statistically significant differences (*p* < 0.05) between each group according to one-way ANOVA. RSDE raw starch degrading enzyme, SSDE soluble starch degrading enzyme.

In addition, whether SANT2 affects amylase production and sporulation was determined in *P*. *oxalicum*. Mutant Δ*SANT2* was successfully constructed and verified by PCR (Supplementary Fig. S13). qPCR determined that a single DNA cassette, containing a DNA fragment encoding PoxRsrA without SANT2, was inserted into the mutant Δ*PoxRsrA* genome (Supplementary Fig. S2). Mutant Δ*SANT2* had similar RSDE and SSDE production to that of Δ*PoxRsrA* (Fig. 4), as well as similar spore production, and colony and mycelial phenotypes (Supplementary Figs. S14 and S15), suggesting that SANT2 is also essential for PoxRsrA functions.

### PoxRsrA interacts with protein POX_g08550

Tandem affinity purification-mass spectroscopy (TAP-MS) was applied to detect proteins that may interact with PoxRsrA. Recombinant protein His-PoxRsrA_901–1360_-GFP was produced by *E. coli* and used as a probe protein to search for interacting proteins. The total proteins from *P. oxalicum* WT Δ*PoxKu70*, cultured in medium containing SCS, were extracted, and used as target proteins (Supplementary Fig. S16). In vitro TAP-MS analysis detected 12 candidate proteins that interacted with PoxRsrA_901–1360_ (Supplementary Table S1).

**Fig. 5:**
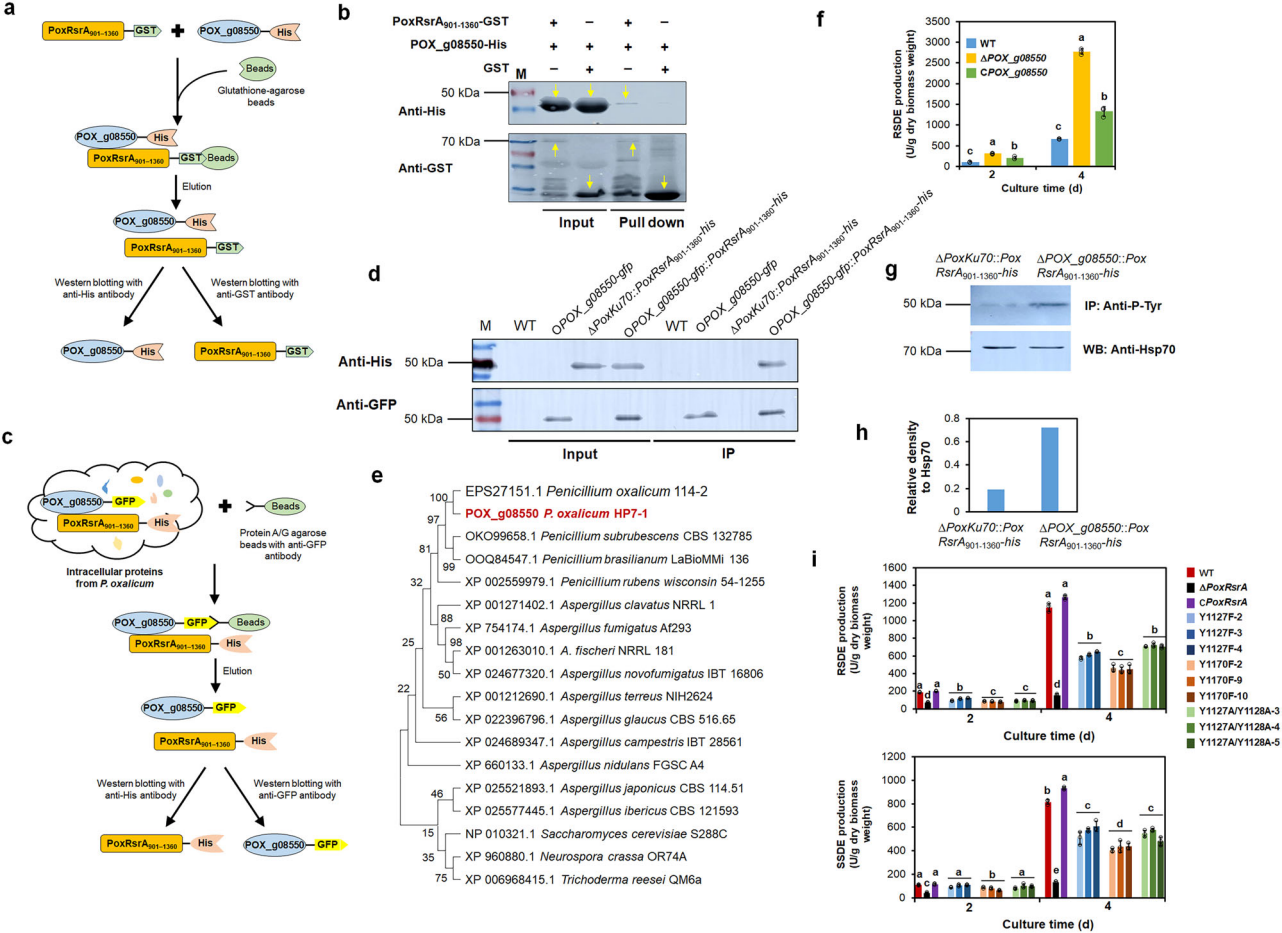
Interaction between POX_g08550 and PoxRsrA, and effects of POX_g08550 on phosphorylation of PoxRsrA, RSDE and SSDE production by *P. oxalicum*. **a** Schematic illustration indicating principle of Glutathione S-transferase (GST)-pulldown assay. **b** GST-pulldown assay between PoxRsrA_901-1360_ and POX_g08550. GST-tagged PoxRsrA_901-1360_ and histidine (His)-tagged POX_g08550 were recombinantly expressed and purified in *Escherichia coli*, and GST was used as control. Yellow arrows indicate target proteins of the right size detected by Western blotting with anti-GST and anti-His antibodies. “+” and “−” mean with and without proteins, respectively. **c** Schematic illustration indicating principle of Co-Immunoprecipitation assay. **d** Co-Immunoprecipitation assay between PoxRsrA_901-1360_ and POX_g08550. *P. oxalicum* strains were cultured in medium containing soluble corn starch for 48 h, then total proteins were extracted. The target proteins were detected by Western blotting with anti-GFP and anti-His antibodies. **e** Phylogenetic analysis of POX_g08550 and its homologous proteins. **f** RSDE production by *P. oxalicum* mutant Δ*POX_g08550*, complementation strain C*POX_g08550* and WT. Fungal strains were pre-cultured in glucose medium for 24 h, then transferred into medium containing soluble corn starch and cultured for 2–4 days. Values marked with different lowercase letters indicate statistically significant differences (*p* < 0.05) between each group according to one-way ANOVA. **g** Western blotting indicating phosphorylation level of PoxRsrA_901-1360_ at tyrosine (Tyr; Y). M: Protein marker. The phosphorylated protein was enriched by anti-His antibody, then detected with anti-P-Tyr antibody. The heat shock protein Hsp70 was used as control. IP: Immunoprecipitation. **h** Relative density of bands corresponding to the phosphorylated PoxRsrA_901-1360_, normalized to that of Hsp70 in (**e**). **i** Identification of key phosphorylated Tyr residues for controlling RSDE and SSDE production. *P*. *oxalicum* site-mutants Y1127F, Y1170F and Y1127A/Y1128A, deletion mutant Δ*PoxRsrA*, complementation strain C*PoxRsrA* and WT were pre-cultured in glucose medium for 24 h, then transferred into medium containing soluble corn starch and cultured for 2–4 days. Each mutant includes three independent transformants. Data values indicate mean ± standard deviation. All experiments were performed at least three times. Values marked with different lowercase letters indicate statistically significant differences (*p* < 0.05) between each group according to one-way ANOVA. RSDE raw starch degrading enzyme, SSDE soluble starch degrading enzyme, WT wild-type strain Δ*PoxKu70*.

In addition, both Glutathione S-transferase (GST)-pulldown and Co-immunoprecipitation (IP) assays were employed for further confirmation of the above interactions. Only the experiments confirming interaction between PoxRsrA_901–1360_ and candidate POX_g08550 are shown; the 11 other candidates require further study. For the GST-pulldown assay, the fusion proteins PoxRsrA_901–1360_-GST and POX_g08550-His were produced by *E*. *coli*, and purified. PoxRsrA_901–1360_-GST and POX_g08550-His were incubated with Glutathione-agarose beads. Complexes recovered from the beads were analyzed by Western blotting (Fig. 5a). Bands corresponding to POX_g08550-His (46 kDa) and PoxRsrA_901–1360_-GST (70 kDa) were observed from pulldown solution from the beads, with anti-His and anti-GST antibodies, respectively. No band appeared when incubating POX_g08550-His and GST as control (Fig. 5b), confirming that PoxRsrA_901–1360_ interacts specifically with POX_g08550.

To confirm that PoxRsrA_901–1360_ and POX_g08550 interact in vivo, *P*. *oxalicum* mutants Δ*PoxKu70*::*PoxRsrA*_901-1360_-*his*, O*POX_g08550-gfp*::*PoxRsrA*_901-1360_-*his* and O*POX_g08550-gfp* were constructed, in which the expression of fused *PoxRsrA*_901-1360_-*his* and *POX_g08550-gfp* were controlled by the constitutive promoter, Ptef1 and the natural promoter of *POX_g08550*, respectively (Supplementary Fig. S17). Total intracellular proteins from the above-mentioned three mutants and WT were each extracted and incubated with Protein A/G agarose beads with anti-GFP antibody. The complexes recovered from the beads were analyzed by Western blotting (Fig. 5c). Bands corresponding to PoxRsrA_901–1360_-His (50 kDa) and POX_g08550-GFP (50 kDa) from the bead eluate were observed, after loading the proteins from O*POX_g08550-gfp*::*PoxRsrA*_901-1360_-*his* with anti-His and anti-GFP antibodies, respectively. A band corresponding to POX_g08550-GFP was observed from the bead eluate, after loading proteins from O*POX_g08550-gfp*, but no band was observed from WT or Δ*PoxKu70*::*PoxRsrA*_901-1360_-*his* (Fig. 5d). These results confirm that PoxRsrA_901–1360_ and POX_g08550 can physically interact in vivo.

POX_g08550 contained 282 amino acid residues, an ECH_1 domain (IPR001753; residues 16–281) and shared 99.7, 77.0, 63.2 and 21.9% identities with PDE_02094 (EPS27151.1) from *P. oxalicum* 114-2, AFUA_3G14520 (XP_754174.1) from *Aspergillus fumigatus* Af293, enoyl-CoA hydratase/isomerase (XP_960880.1) from *Neurospora crassa* OR74A and 3-hydroxyisobutyryl-CoA hydrolase (NP_010321.1) from *Saccharomyces cerevisiae* S288C, respectively. Evolutionary analysis indicated that POX_g08550 was closely related to the corresponding proteins in *Penicillium* and *Aspergillus* (Fig. 5e).

To analyze the functions of POX_g08550 in *P. oxalicum*, the deletion mutant Δ*POX_g08550* and complementation strain C*POX_g08550* were constructed by homologous recombination. In C*POX*_*g08550*, the complementation cassette of *POX_g08550* was introduced into an ectopic locus of *POX_d05452* (Supplementary Fig. S18). qPCR determined that a single DNA cassette was inserted into the WT and Δ*POX_g08550* genomes (Supplementary Fig. S2).

When cultured on SCS for 2–4 days, RSDE production by Δ*POX_g08550* was 1.0–2.1-fold higher than those of WT and the complementation strain C*POX_g08550* (Fig. 5f), suggesting that POX_g08550 down-regulates the biosynthesis of RSDEs in *P. oxalicum*. Notably, the complementation strain C*POX_g08550* did not fully complement the phenotype of the Δ*POX_g08550* (Fig. 5f), which may result from different effects related to the alternative locus (*POX_d05452*) integrated by the complementation cassette.

### POX_g08550 inhibits tyrosine phosphorylation in PoxRsrA

To determine whether POX_g08550 influences the phosphorylation of PoxRsrA, total protein extracts from mutants Δ*PoxKu70*::*PoxRsrA*_901–1360_-*his* and Δ*POX_g08550*::*PoxRsrA*_901–1360_-*his* (Supplementary Fig. S17) were analyzed by Western blotting with anti-Tyr antibody. Tyrosine (Y) phosphorylation of *PoxRsrA*_901–1360_ in Δ*POX_g08550*::*PoxRsrA*_901-1360_-*his* markedly increased, compared with that of Δ*PoxKu70*::*PoxRsrA*_901-1360_-*his* (Figs. 5g, h and S19). IP-mass spectrometry (MS) found that Y1127, Y1128 and Y1170 in PoxRsrA_901-1360_ were phosphorylated in Δ*POX_g08550*::*PoxRsrA*_901-1360_-*his*, but not in Δ*PoxKu70*::*PoxRsrA*_901-1360_-*his*, indicating that the phosphorylation of these amino acids was inhibited by POX_g08550 (Supplementary Fig. S20).

### Phosphorylation of PoxRsrA on tyrosine affects its functions

To investigate the effects of Y1127, Y1128 and Y1170 phosphorylation on amylase production in *P. oxalicum*, three *PoxRsrA* mutants, encoding PoxRsrA^Y1127F^, PoxRsrA^Y1170F^ and PoxRsrA^Y1127A/Y1128A^ were respectively introduced into the mutant Δ*PoxRsrA* at the *POX_d05452* site, to generate mutants Y1127F, Y1170F and Y1127A/Y1128A (Supplementary Fig. S21). qPCR determined that a single DNA cassette was inserted into the Δ*PoxRsrA* genome (Supplementary Fig. S2). Enzyme assays revealed that RSDE and SSDE production by Y1127F, Y1170F and Y1127A/Y1128A was markedly higher than that of Δ*PoxRsrA*, but lower than that of the complementation strain C*PoxRsrA* after 4 days of SCS induction. Notably, Y1127F and Y1127A/Y1128A produced similar amounts of RSDE and SSDE, but much more than Y1170F (Fig. 5i). However, at 2 days, overall enzyme production by Y1127F, Y1170F and Y1127A/Y1128A was higher than that of Δ*PoxRsrA*, whereas RSDE production by all three and SSDE production by Y1170F were lower than those of C*PoxRsrA*. In addition, SSDE production by both Y1127F and Y1127A/Y1128A was higher than Y1170F, whereas only RSDE production by Y1127F was higher than Y1170F (Fig. 5i). Therefore, it appears that phosphorylation of Y1127 and Y1170 up-regulates RSDE and SSDE biosynthesis in *P. oxalicum*, and phosphorylation of Y1170 appears to have the greater effect, mainly during the late period of induction.

Furthermore, phenotypic observation found colony color and diameter variation of Y1127F on PDA, compared with C*PoxRsrA*, as shown in Supplementary Figs. S22–S24.

### PoxRsrA interacts with four Mediator complex subunits

**Fig. 6:**
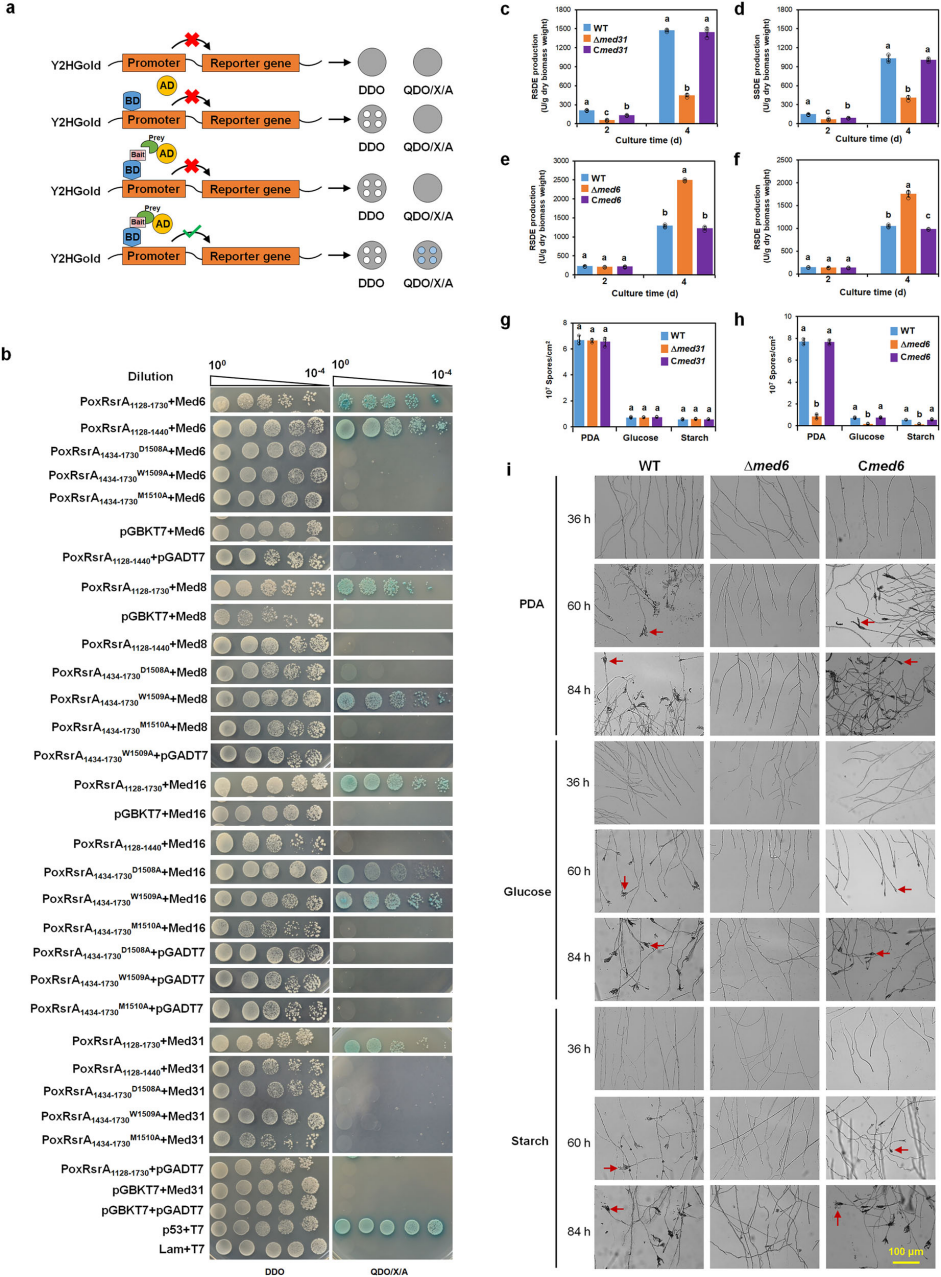
Identification of Mediator complex subunits interacting with PoxRsrA. **a** Schematic illustration showing principle of yeast two-hybrid assay. BD: DNA-binding domain. AD: transcriptional activation domain. DDO: synthetic dropout medium (SD) without tryptophan and leucine (SD/-Trp/-Leu). QDO/X/A: SD-Trp/-Leu/-Ade/-His/+X-α-Gal/+Aureobasidin A. **b** Yeast two-hybrid assay of PoxRsrA and Mediator subunits Med6, Med8, Med16 and Med31. Yeast cells carrying different lengths of PoxRsrA or site-mutated PoxRsrA as the bait and Mediator subunits as the prey were diluted by a 10-fold gradient and then cultured on DDO, or QDO/X/A for 4 days. The original concentration of each yeast sample was adjusted to OD_600_ = 1.0. **c**, **d** Effects of Med31 and Med6 (**e**, **f**) on RSDE and SSDE production. All tested *P. oxalicum* mutants Δ*med31* and Δ*med6*, complementation strains C*med31* and C*med6* and the wild-type strain (WT) were cultured in soluble corn starch medium for 2–4 days after transfer from glucose. **g** Effects of Med31 and Med6 (**h**) on number of asexual spores produced. Strains were cultured on solid plates containing various carbon sources for 5 days, respectively. In (**c**–**h**), data values indicate mean ± standard deviation. All experiments were performed at least three times. Values marked with different lowercase letters indicate statistically significant differences (*p* < 0.05) between each group according to one-way ANOVA. RSDE raw starch degrading enzyme; SSDE soluble starch degrading enzyme. **i** Effects of Med6 on mycelial development. *P. oxalicum* strains were cultured on solid plates containing potato dextrose agar (PDA), glucose or soluble corn starch for 36–84 h. The red arrows indicate conidiospores. Scale bar = 100 μm.

To determine which Mediator subunits interacted with PoxRsrA, polypeptide PoxRsrA_1128-1730_ was used as the bait in the yeast two hybrid system (Y2H) assay. Of the Mediator subunits annotated in the *P. oxalicum* strain HP7-1 genome^13^, the whole proteins of 22 subunits were used as candidate preys. The reporter genes were activated only when the prey and bait interacted (Fig. 6a), thereby allowing the yeast cells to grow normally, both on DDO and QDO/X/A plates. The Y2H assay revealed that yeast Y2HGold cells supplying PoxRsrA_1128-1730_ and Med6, Med8, Med16 or Med31, grew well on DDO and QDO/X/A plates. In addition, yeast colonies on QDO/X/A were blue, as well as positive cells carrying p53 and T7. Yeast Y2HGold cells carrying PoxRsrA_1128-1730_ and other Mediator subunits, as well as the negative controls, such as Lam and T7, only grew on DDO (Fig. 6b and Supplementary Fig. S25). This indicates that PoxRsrA_1128-1730_ interacts with four Mediator subunits Med6 (POX_c04649), Med8 (POX_b02367), Med16 (POX_d05606) and Med31 (POX_g08593) (Fig. 6b).

In addition, a truncated PoxRsrA_1128-1440_ was found, via Y2H assays, to interact with Med6, but not with Med8, Med16 and Med31. Therefore, it appears that PoxRsrA_1434-1730_ interacted with Med8, Med16 and Med31. However, PoxRsrA_1434-1730_ had transcriptional autoactivation ability, but mutated PoxRsrA_1434–1730_^D1508A^, PoxRsrA_1434–1730_^W1509A^ and PoxRsrA_1434–1730_^M1510A^ did not, so they were suitable for further Y2H assays. Interactions were found between mutated PoxRsrA_1434–1730_^W1509A^ and Med8, PoxRsrA_1434–1730_^D1508A^ and Med16, as well as PoxRsrA_1434–1730_^W1509A^ and Med16, whereas other combinations did not interact (Fig. 6b). It appears that PoxRsrA_1434–1730_ interacts with Med8, Med16 and Med31, and that D1508 and/or M1510 are required for PoxRsrA_1434–1730_ to interact with Med8 and Med16, as well as D1508, W1509 and M1510 for PoxRsrA_1434–1730_ to interact with Med31.

### Med6 and Med31 exhibit opposite functions in *P*. *oxalicum*

To elucidate the effects on mycelial growth, RSDE and SSDE production of the four Mediator subunits that interact with PoxRsrA_1128-1730_, the deletion mutants Δ*med6* and Δ*med31*, and the knockdown mutants Ptcu_*med8* and Ptcu_*med16*, were constructed (Supplementary Fig. S26). qPCR determined that a single DNA cassette was inserted into the WT genome (Supplementary Fig. S2). In Ptcu_*med8* and Ptcu_*med16*, the native promoters of *med8* and *med16* were replaced by the copper-responsive promoter Ptcu of the copper transporter-encoding gene *tcu1*. The promoter Ptcu was inhibited in *P. oxalicum* when cultured in medium with a high concentration of copper ions, such as 5 μM^15^. RT-qPCR indicated that the expression of *med8* and *med16* in Ptcu_*med8* and Ptcu_*med16* were markedly repressed when cultured with 5 μM Cu^2+^ (Supplementary Fig. S26d).

When cultured on SCS for 2–4 days after transfer from glucose, Δ*med31* had 60.2–69.6% decreased RSDE and SSDE production (Fig. 6c, d), whereas Δ*med6* had 74.8–75.4% increased amylase production at 4 days, compared with WT (Fig. 6e, f). Ptcu_*med8* cultured with 5 μM Cu^2+^ for 4 days had 18.4–43.6% reduced RSDE and SSDE production, relative to WT (Supplementary Fig. S27a). The RSDE and SSDE production of Ptcu_*med16* were similar to those of WT (Supplementary Fig. S27b).

Moreover, Δ*med6* produced white colonies on PDA, whereas WT was turquoise; no significant difference was found between Δ*med6* and WT on either glucose or SCS (Supplementary Fig. S28). Δ*Med31* produced a similar colony phenotype and sporulation as WT (Fig. 6g and Supplementary Figs. S28 and S29). Δ*med6* had delayed conidiophore formation and reduced spore production on PDA, glucose and SCS, compared with WT (Fig. 6h and i).

### Overexpression of *PoxRsrA* in *P*. *oxalicum* enhances amylase production and mycelial growth

To determine the feasibility of *PoxRsrA* as a target for enhancing RSDE and SSDE production, the overexpression strain O*PoxRsrA* was constructed, in which *PoxRsrA* was controlled by the constitutive promoter, Ptef1 (Supplementary Fig. S30a and b). qPCR determined that a single DNA cassette was inserted into the WT genome (Supplementary Fig. S2). In addition, RT-qPCR determined that transcription of *PoxRsrA* in overexpression strain O*PoxRsrA* was enhanced by 10.8–18.8-fold, compared with WT (Supplementary Fig. S30c).

**Fig. 7:**
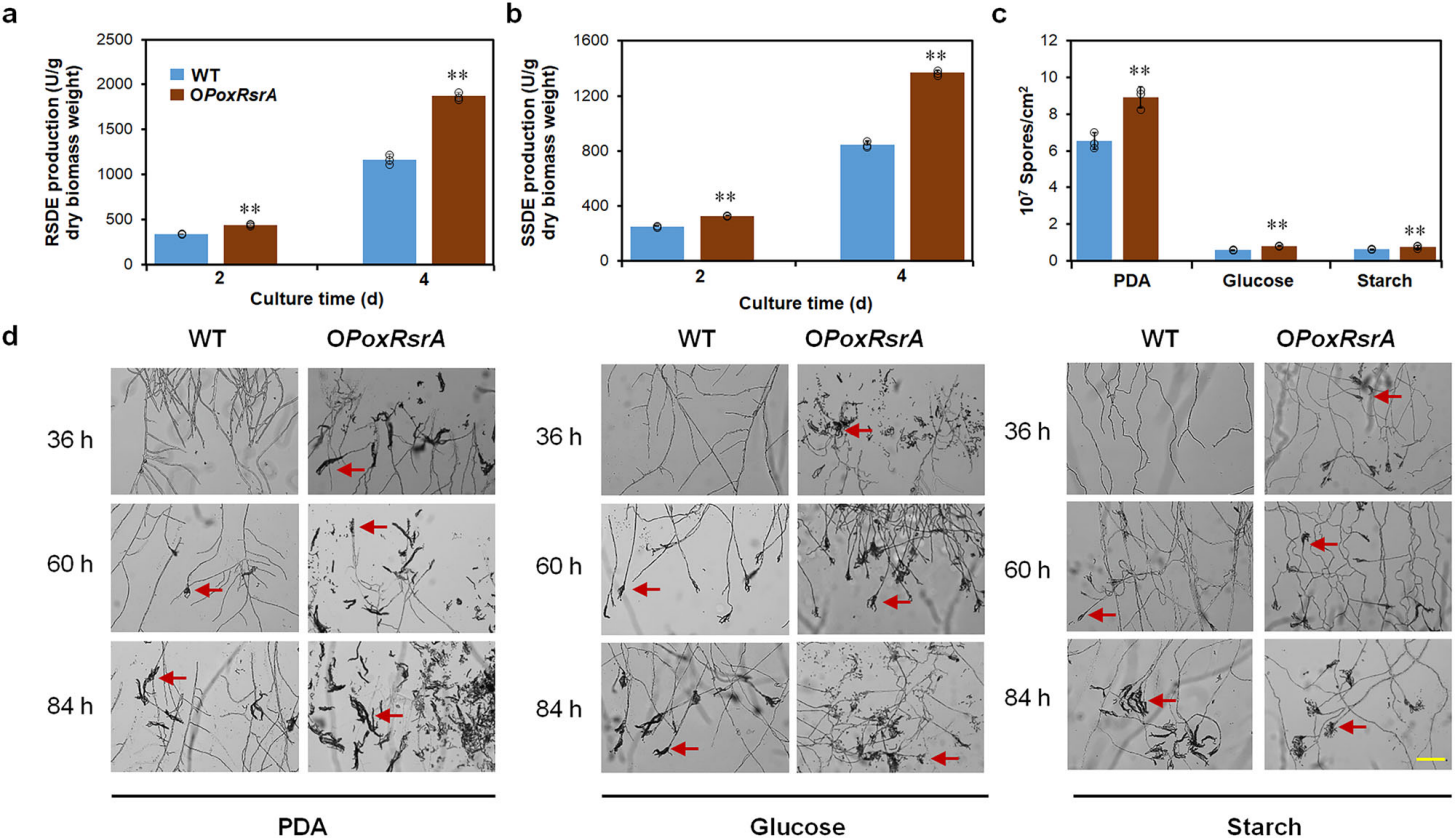
Effects of overexpression of PoxRsrA on production of amylases and spores by *P. oxalicum* when cultivated in the presence of various carbon sources. **a** RSDE production. **b** SSDE production. All tested *P. oxalicum* strains were cultured in soluble corn starch medium for 2–4 days after transfer from glucose. WT: the wild-type strain. **p < 0.01 indicates significant differences between the overexpression strain and parental strain, assessed by Student’s *t* test. RSDE raw starch degrading enzyme; SSDE soluble starch degrading enzyme. **c** Spore production. Number of asexual spores was measured after growth on solid plates containing different carbon sources for 5 days and mycelial growth assessed after 36–84 h. **d** Mycelial development. The red arrows indicate conidiospores. PDA potato dextrin agar. Scale bar = 100 μm.

RSDE and SSDE production by O*PoxRsrA* was enhanced by 30.0–61.6% compared with WT, after SCS induction for 2–4 days (Fig. 7a and b). Moreover, O*PoxRsrA* had increased colony size on PDA and glucose (Supplementary Fig. S31), as well as increased conidiospore formation and spore count on PDA, glucose, or starch (Fig.7c and d).

## Discussion

**Fig. 8:**
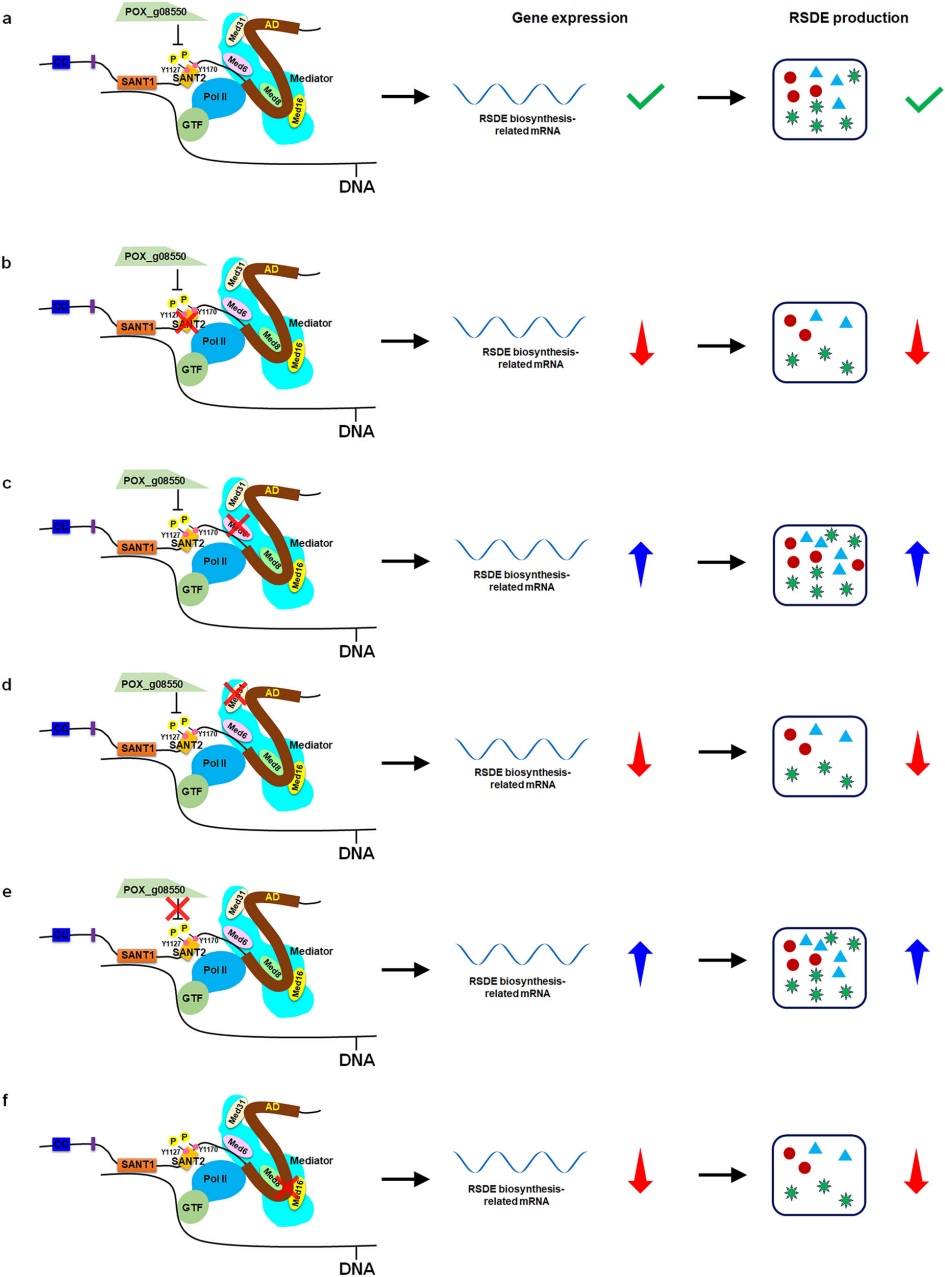
A proposed model of PoxRsrA regulation in *P. oxalicum* in the presence of soluble corn starch. After receiving inductive signals, the SANT1 domain in PoxRsrA is responsible for binding to DNA sequences located in the promoter regions of target genes, meanwhile, the C-terminus of PoxRsrA recruits the transcription pre-initiation complex by binding four Mediator complex subunits, which activates the expression of RSDE biosynthesis-related genes, then initiates RSDE biosynthesis (**a**). Reduced phosphorylation of tyrosine Y1127 in SANT2 results in decreased expression of RSDE biosynthesis-related genes and lower RSDE production (**b**); interaction between Med6 and PoxRsrA_1128_–_1440_ inhibits the transcription of RSDE biosynthesis-related genes and RSDE production (**c**); interaction between Med31 and the transcription activation domain (AD) stimulates the expression of RSDE biosynthesis-related genes and RSDE production (**d**), POX_g08550 represses the phosphorylation of Y1127 and Y1170, thereby reducing expression of RSDE biosynthesis-related genes and RSDE production (**e**); inhibition of the interaction between PoxRsrA and Med8 or Med16 inhibits the production of RSDE (**f**). Pol II: RNA polymerase II. GTF general transcription factor. RSDE raw-starch-degrading enzyme. Y1127 and Y1170 represent the tyrosine residues at positions 1127 and 1170, respectively. A red cross indicates a deletion. A green check mark indicates normal gene expression and RSDE production in wild-type *P*. *oxalicum*. Red and blue arrows represent decrease and increase, respectively. Various shapes included in the rectangle show different types of RSDE.

In this study, the DNA-binding and transcription activation domains of TF PoxRsrA, as well as key amino acid residues and one interacting protein were identified. Furthermore, phosphorylation of PoxRsrA appeared to enhance RSDE biosynthesis. Two Mediator subunits, Med6 and Med31, which interacted with PoxRsrA, had different effects on RSDE biosynthesis in *P. oxalicum* (Fig. 8). These findings enable a deeper understanding of the mechanisms of fungal RSDE production and provide a solid basis for breeding of fungi to improve RSDE yields.

Truncated peptide design and yeast auto-stimulation experiments confirmed that PoxRsrA_1434–1730_ contained the transcription activation domain. Three adjacent residues, D-W-M (1508–1510) appeared to be essential for transcriptional activation and RSDE and SSDE production; they were highly conserved in PoxRsrA and its homologs. However, substitution of D-W-M did not affect spore production by *P. oxalicum*, so further study will be needed to fully elucidate the mechanisms involved.

Notably, the Y2H assay indicated that PoxRsrA_1434–1730_ had transcriptional activation ability, whereas the full-length protein (PoxRsrA_1–1730_) and other sections of PoxRsrA (e.g., PoxRsrA_1128–1730_) did not, speculating that the reason may be that protein folding and tertiary structures in yeast are different from those in *P. oxalicum*.

PoxRsrA contains two SANT-like domains^4^ with different functions. SANT domains are commonly found in proteins participating in chromosome remodeling and transcription regulation, such as Swi3, Ada2, N-CoR and TFIIIB. They can bind to DNA sequences, as they are structurally similar to the DNA-binding Myb family^16^. The SANT1 of PoxRsrA could bind to the promoter regions of genes encoding major amylases, but the SANT2 could not, which might be attributed to residue, R866. R866 may form a salt bridge with another conserved residue, E841^17^. Comparatively, the residue corresponding to R866 in SANT2 is K1119, which eliminates a structurally-important salt bridge and disrupts the three-dimensional structure.

Alteration of R866 to either K or A resulted in complete loss of PoxRsrA regulation of RSDE and SSDE production during the early period of induction by SCS, and partial loss during the late period of induction. It appears that the mutated SANT1 is unable to bind to the promoters of genes encoding major amylases. However, the mutant protein may regulate amylase biosynthesis by another unknown mechanism, during the late period of induction, such as by indirectly controlling the expression of related regulatory genes. The mutant R866A also had higher RSDE and SSDE production than R866K, which may be attributable mutated SANT1^R866K^ and SANT1^R866A^ binding to different target genes, but further research is needed to investigate this potential mechanism of action.

SANT domains also interact with proteins, such as histone H4 or non-histone HMG1 or Sin1^16,18^. Here, POX_g08550, annotated as a putative 3-hydroxyisobutyryl-CoA hydrolase, involved in fatty acid β-oxidation/lipid metabolism^19^, is confirmed to interact with SANT2 of PoxRsrA. POX_g08550 down-regulated RSDE production by inhibiting phosphorylation of Y1127 and Y1170, which is required for up-regulation of RSDE production by PoxRsrA. Furthermore, mutation of the phosphorylation sites, Y1127 and Y1170, to A in PoxRsrA resulted in decreased RSDE production. Previous evidence indicates that phosphorylation of TFs is required for their normal function^20,21^. However, the detailed mechanism of the effects of phosphorylation on the function of PoxRsrA remains unclear.

Western blotting showed tyrosine phosphorylation of PoxRsrA_901–1360_ in both Δ*POX_g08550* and WT, which appeared to contradict the IP-MS data, indicating phosphorylation of Y1127, Y1128 and Y1170 in Δ*POX_g08550*::*PoxRsrA*_901–1360_-*his*, but no phosphorylation of Δ*PoxKu70*::*PoxRsrA*_901–1360_-*his*. However, it is common for only partial phosphorylation of protein molecules to occur. It is possible that the non-phosphorylated PoxRsrA_901–1360_ molecules in the mutant ∆*POX_g08550* were detected by IP-MS. In addition, the possibility cannot be excluded that other tyrosine residues were phosphorylated, but not detected by IP-MS.

Notably, the absence of SANT2 resulted in similar enzyme production and phenotype to that of Δ*PoxRsrA*, which may be attributable to non-phosphorylation of Y1127. However, this did not exclude the possibility of disruption of the PoxRsrA 3D structure as a result of the absence of SANT2.

PoxRsrA could interact with four Mediator subunits, Med6, Med8, Med16 and Med31, as determined by the Y2H assay. Notably, Med6 and Med31 bound to different sites in PoxRsrA, namely PoxRsrA_1128–1440_ and PoxRsrA_1434–1730_, whereas Med8 and Med16 appeared to interact with the same region, because D1508 and/or M1510 were required for their interactions. In the structure of the Mediator complex, Med6 and Med8 located in the head module, whereas Med31 and Med16 are in the middle and tail modules, respectively^8^.

Notably, Med6 and Med31 had different functions in *P. oxalicum*, i.e., Med6 activated mycelial sporulation, but suppressed RSDE production, whereas Med31 only activated RSDE production. In *Drosophila* and yeast, a deficiency of Med6 leads to transcriptional defects in a wide range of developmental genes^22,23^. Apparently, Med6 in filamentous fungi evolved additional function of regulating RSDE production, through recruitment as PoxRsrA_1134–1440_. However, the regulatory effects of PoxRsrA and Med6 on RSDE biosynthesis were opposite, implying that other regulatory proteins were involved, which needs further research for a more understanding.

Med31 is functionally diverse in eukaryotes, for instance, in *S. cerevisiae*, the Δ*med31* mutant grew slowly, but in *Tetrahymena thermophila*, Med31 down-regulates the expression of developmental genes^24^. In *Fusarium verticillioides*, Med31 is involved in regulation of mycelial growth, conidiation, virulence and secondary metabolism^11^. However, in *P. oxalicum*, Med31 participates in regulation of enzymatic biosynthesis, rather than growth and development, through recruitment of PoxRsrA_1434–1730_.

Surprisingly, Med8 and Med16 could not be deleted from *P. oxalicum*, indicating that they were essential for mycelial growth and their deletion was lethal. Therefore, the knockdown mutants Ptcu_*med8* and Ptcu_*med16* were constructed, and had slightly reduced or similar RSDE production compared with the background strain. Med8 has essential regulatory functions in growth and development of yeast and plants^25,26^. Med16 is involved in fungal development, the stress response and secondary metabolism in *T. reesei*^11^, but not in controlling the expression of genes encoding major cellulases^27^. Site-mutation of D1508 and/or M1510, which are required for the interaction between PoxRsrA and Med8 or Med16, resulted in markedly reduced RSDE production, suggesting that their binding interactions are very important for the proper function of PoxRsrA, possibly because these interactions were responsible for the stability of the PoxRsrA-Mediator complex.

It should be noted that the interactions between Mediator subunits and PoxRsrA were only demonstrated through the Y2H assay, which was not sufficient to demonstrate that they interacted similarly in *P*. *oxalicum*; confirmation of this requires additional in vivo evidence.

Surprisingly, mutants ∆1–565 and ∆1–467 showed enhanced production of RSDE and SSDE, compared with the control strain Ptef1-*PoxRsrA*. However, the increase in amylase production resulting from deletion of *PoxRsrA*_*1–565*_ was lower than that from deletion of *PoxRsrA*_*1–467*_, suggesting that PoxRsrA_468–565_ promotes amylase biosynthesis, whereas PoxRsrA_1–467_ suppresses amylase biosynthesis in *P. oxalicum*. However, the mechanism by which PoxRsrA_1–467_ and PoxRsrA_468–565_ have opposite effects on amylase biosynthesis is unknown, and requires further investigation. In addition, most of the C-terminal truncation mutants had lower enzyme production than the ∆*PoxRsrA* null mutant and ∆1440–1794, possibly because of the different functions of different regions of PoxRsrA polypeptides, as described above.

## Methods

### Microorganisms and culture conditions

This study used strains of *P. oxalicum*, *S. cerevisiae* and *E. coli* (Supplementary Table S2). *P. oxalicum* strains were cultured on PDA (Becton, Dickinson and Company, Sparks, MD) plates for 5 days at 28 °C to produce asexual spores. Tween 80 (Sangon Biotech Co. Ltd., Shanghai, China) was used to harvest asexual spores for reproduction.

Asexual spores (1 × 10^8^) were cultured on modified minimal medium containing SCS (Sigma-Aldrich, St. Louis, MO, USA)^5^, then used for measurement of RSDE and SSDE activities and for RT-qPCR assay. Solid modified minimal medium containing glucose, SCS or PDA were used for investigation of colony and mycelial development.

*S. cerevisiae* and *E. coli* strains were grown on yeast extract peptone dextrose (YPD) medium and Luria-Bertani (LB) medium for preservation and/or reproduction, respectively.

### Nucleic acid manipulation

Mycelia of *P. oxalicum* strains were fractured by liquid nitrogen, then dissolved in DNA extraction buffer (g/L: EDTA 3.72, SDS 10, NaAc·3H_2_O 2.72, Tris 4.864, pH 8.0)^5,28^. Phenol-chloroform was added to remove proteins, then the DNA was precipitated with anhydrous ethanol (Tianjin Fuyu Fine Chemicals Co. Ltd., China). Finally, the DNA was dissolved in ddH_2_O for further use.

Total RNA extraction of *P. oxalicum* strains by RNAsimple Total RNA Kit (Tiangen Biotech Co. Ltd., Beijing, China) was conducted according to the manufacturer’s instructions.

Plasmid extraction from *E. coli* was carried out with a Plasmid Minipreparation Kit (YFXM0038; Yifeixue Bio Tech, Nanning, China).

### *P. oxalicum* protoplast preparation

Fresh asexual spores of *P. oxalicum* were inoculated into completed medium (peptone 2.0 g/L, MnSO_4_·H_2_O 0.8 mg/L, acid hydrolyzed casein 1.0 g/L, D-glucose 10.0 g/L, yeast extract 1.0 g/L, FeSO_4_·7H_2_O 2.5 mg/L, ZnCl_2_ 0.85 mg/L, CoCl_2_ 1 mg/L, 20× nitrate solution 50 ml/L), then cultured at 180 rpm and 28 °C for 9–12 h. The harvested culture was centrifuged on horizontal centrifuge to collect mycelia. The mycelia were washed twice with sterile water. The protoplasts of *P. oxalicum* were obtained by removing cell walls of mycelia using enzymatic lysate (lysozyme 4.0 g/L, lysing enzyme from *Trichoderma harzianum* 6.0 g/L, snailase 6.0 g/L, MgSO_4_·7H_2_O 1.2 M, and NaH_2_PO_4_ 10 mM). Extraction of protoplasts from the mixture was carried out with trapping buffer (sorbitol 72.9 g/L and Tris 12.2 g/L). The protoplasts were rinsed with 1 M sorbitol (183.8 g/L) and STC (sorbitol 182.0 g/L, Tris 12.0 g/L, CaCl_2_ 11.2 g/L) twice, respectively. Finally, the mixture of STC and PTC (PEG3350 400 g/L, Tris 12.0 g/L, CaCl_2_ 11.2 g/L) was added at volume ratio of STC : PTC = 4 : 1, and the protoplasts of *P. oxalicum* were re-suspended and stored at −80 °C for further use.

### Transformation of *P. oxalicum* protoplast with polyethylene glycol-mediated chemical method

DNA cassette of 2–5 μg was mixed with 100 mM spermidine of 4 μL (Solarbio Life Sciences, Beijing, China). The mixture was added to the protoplasts of *P. oxalicum*, then placed on ice for 30 min. Subsequently, PTC solution of 1 mL was added, and kept at 28 °C for 25 min. After added STC solution of 2 mL, the mixture was mixed with OCM medium (yeast extract 1.0 g/L, casein enzymatic hydrolysate 1.0 g/L, sucrose 342.0 g/L, agar 20.0 g/L), and poured into sterile petri dish. The medium mixture was kept for 30 min. PDA medium containing antibiotics G418 (800 μg/mL) or bleomycin (100 μg/mL) was added and then cultured at 28 °C for 4 days to obtain transformants.

### Construction of *P. oxalicum* mutants

Construction of *P. oxalicum* mutants was performed based on homologous recombination^28^. The primers for DNA cassette construction and recombinant confirmation are listed in Supplementary Data 1. All DNA fragments were obtained by PCR amplification with specific primers. The cassettes for mutant construction were generated by fusion PCR, then introduced into the corresponding *P. oxalicum* protoplasts by the polyethylene glycol-mediated chemical method. The transformants were screened with the antibiotic bleomycin (100 μg/mL) and/or G418 (800 μg/mL), then verified by PCR with specific primers.

### Observation of *P. oxalicum* colony phenotype and mycelial development

Fresh asexual spores (1 × 10^8^) of *P. oxalicum* strains were inoculated directly onto solid plates containing PDA, glucose, or SCS, then cultured at 28 °C for 5 days. *P. oxalicum* colonies were observed with a Canon EOS 6D camera (Canon Inc., Tokyo, Japan) by automated pattern recognition, f-stop = 4.5, time of exposure = 1/100 s, IOS speed = 500, focal length = 105 mm. The mycelia was observed under a light microscope (OLYMPUS DP480, Tokyo, Japan), and the parameters were set as 20× objectives, 10× eyepieces, time of exposure = 30 ms, IOS speed = 400. Photomicrographs were analyzed in cellSence Dimension digital imaging software (Olympus)^5^.

### RT-qPCR assay

The relative gene expression in *P. oxalicum* strains was analyzed by RT-qPCR^28^. The total RNA extract from *P. oxalicum* strains was converted to single stranded cDNA by HiScript III RT SuperMix for qPCR (+gDNA wiper) (Vazyme Biotech Co, Ltd., Nanjing, China). The tested genes were detected by PCR amplification with ChamQTM Universal SYBR qPCR Master Mix (Vazyme), using the synthesized cDNA as template, and suitable primers (Supplementary Data 1), on a 7500 Real Time PCR System. The relative expression of each gene detected was analyzed by the 2^−ΔΔCT^ method^29^.

### Determination of RSDE and SSDE activities

The RSDE and SSDE activities of crude enzyme from *P. oxalicum* were determined by the 3,5-dinitrosalicylic acid method^30^, with raw cassava flour, or SCS as substrates, respectively^31^. In brief, crude enzyme solution (50 μL), extracted from *P*. *oxalicum* was added to citrate-phosphate buffer (450 μL, 0.1 M, pH 4.5/5.0) containing 1% (w/v) raw cassava flour, or SCS, then reacted at 65 °C, or 55 °C for 30 min, respectively. The reaction was stopped by heating in boiling water for 10 min. The inactivated crude enzyme was used as control. The released reducing sugar was detected with dinitrosalicylic acid^30^. One unit (U) of enzyme activity was defined as the amount of enzyme required to produce 1 μmoL of reducing sugar per min. RSDE and SSDE production was expressed as U/gram of dry mycelial weight.

### Subcellular localization

Subcellular localization of PoxRsrA in the hyphae of *P. oxalicum* was determined with GFP as the reporter. Gene *gfp* was inserted at 3’-terminus of *PoxRsrA* by replacing the stop code in the background strain Δ*PoxKu70*, to form a fusion gene *PoxRsrA-gfp*. The obtained mutant PoxRsrA::GFP was cultivated in modified minimal medium containing 1% SCS or 1% glucose for 24 h at 28 °C. The culture was centrifuged on horizontal centrifuge to collect mycelia, then washed twice with sterile water. Mycelial nucleus was stained by DAPI staining solution (Solarbio Life Sciences, Beijing, China). The hyphae were observed under a fluorescence microscope (OLYMPUS DP480; Olympus). The parameters were set as 40× objectives, 10× eyepieces, time of exposure 40 ms, IOS speed 400. The images were taken with white excitation source, green excitation source and blue excitation source respectively, and analyzed using cellSence Dimension digital imaging software (Olympus).

### Identification of transcription activation domain (AD) in PoxRsrA

*PoxRsrA* fragments of different lengths were amplified using the cDNA of *P. oxalicum* strain Δ*PoxKu70* as template, with specific primers (Supplementary Data 1), then subcloned into the vector pGBKT7 (TaKaRa, Dalian, China) at the *Eco*R1 and *Bam*H1 restriction enzyme sites. The recombinant plasmids were transformed into *S. cerevisiae* Y2HGold strain (TaKaRa). The resulting transformants were diluted by a 10-fold gradient and then cultured on SDO/X/A plates, with an SDO plate as control. The initial concentration of yeast cells was adjusted to OD_600_ = 1.0. The final concentrations of Aureobasidin A and X-α-gal were adjusted to 100 ng/mL and 0.04 mg/mL, respectively, in SDO/X/A. After 3–5 days of culture at 30 °C, transcriptional activation of two reporter genes, *AUR1-C* and *MEL1*, encoding inositol phosphoryl ceramide synthase and α-galactosidase, resulted in resistance to Aureobasidin A and formation of blue colonies in the presence of X-α-Gal. Growth of the tested transformants on SDO and SDO/X/A plates, with white and blue colonies, respectively, indicated that the tested protein/peptide had transcriptional activation activity (Fig. 1c).

### Y2H assay

One-to-one Y2H assays (Matchmaker^®^ Gold Yeast Two-Hybrid System, TaKaRa) were employed to investigate protein-protein interactions. The recombinant pGBKT7-bait and pGADT7-prey were co-transformed into competent Y2HGold cells. The resulting positive transformants were diluted by a tenfold gradient and then simultaneously spread on QDO/X/A and DDO plates. The initial concentration of yeast cells was adjusted to OD_600_ = 1.0. In QDO/X/A, the final concentrations of X-α-Gal and Aureobasidin A were 1 mol/L and 100 ng/mL, respectively. The Y2HGold strain carrying pGBKT7-p53 and pGADT7-T7 was used as positive control, and multiple pairs including pGBKT7-Lam and pGADT7-T7, pGBKT7 and pGADT7 were employed as negative controls. The expected results were observed after 3–5 days of culture at 30 °C. The white and blue colonies of the tested transformants appeared on DDO and QDO/X/A, respectively, whereas the negative controls only grew on DDO plates with white colonies and could not grow on QDO/X/A plates, which confirmed that the prey and bait had interacted (Fig. 6a).

### LC-MS/MS protein analysis

Protein labeled with GFP, or GST tags was precipitated with the corresponding antibody, then characterized by LC-MS/MS^5^. The recombinant proteins labeled with GFP or His were purified by ChromoTek GFP-Trap^®^ Agarose (ChromoTek, Planegg-Martinsried, Germany), or enriched using the corresponding antibody. The sample was digested with 10 ng/μL trypsin (Sigma-Aldrich) at 37 °C overnight, and the generated peptides were separated and detected by LC-MS/MS on a liquid chromatography system (Waters, Milford, MA) coupled to a Thermo Scientific LTQ-Orbitrap Mass Spectrometer (Thermo Fisher Scientific, Bremen, Germany) according to the manufacturer’s instructions. The used parameters were set as the flow phase A: 2% acetylene, 0.1% methic acid, 97.9% water; the flow phase B: 98% acetylene, 0.1% methic acid, 1.9% water; flow velocity 250 nL/min.ESI positive ion scanning mode was selected. The ion of the mass load (M/Z) 445.12003 was used as internal standard of the instrument, and the electric injection voltage was set as 1.9 kV. Data dependency collection mode was performed with second-level MS/MS analysis. The Sequest search engine of the Proteome DiscoverR 2.1 software retrieved the spectrum obtained by NanoLCMS. The collected spectrum aligning with the theoretical spectrum library of the protein database was scored.

### TAP-MS

The recombinant fusion protein His-PoxRsrA_901–1360_-GFP was expressed in *E. coli*, then used as the bait when mixed with the whole PoxRsrA protein from WT. The mixture was treated twice, successively with ProteinIso® Ni-NTA Resin (TransGen Biotech Co. Ltd, Beijing, China) and ChromoTek GFP-Trap® Agarose (ChromoTek). The isolated peptides/proteins were characterized by LC-MS/MS (Supplementary Fig. S21).

### GST-pulldown

The GST-pulldown assay was performed to confirm protein-protein interactions in vitro^5^. The recombinant protein PoxRsrA_901–1360_-GST and POX_g08550-His were recombinantly expressed in *E. coli*, then purified by ProteinIso® Ni-NTA Resin (TransGen) and *ProteinIso*^®^ GST Resin (TransGen), respectively. Equal amounts of PoxRsrA_901–1360_-GST and POX_g08550-His were incubated on ice for one hour. The mixture was subsequently purified by ProteinIso^®^ GST Resin (TransGen), to detect interacting proteins with PoxRsrA_901–1360_-GST. Monoclonal anti-His and anti-GST antibodies were employed to verify the interactions by Western blotting (Fig. 5a).

### In vitro Electrophoretic Mobility Shift Assay (EMSA)

Protein-DNA interaction was verified by EMSA^5^. DNA probes were amplified by PCR with the corresponding primers (Supplementary Data 1). The reverse primers were added with FAM at the 3′-terminus. DNA fragments encoding the putative DNA-binding domain of PoxRsrA were cloned into the plasmid pET-32a (+) to generate the recombinant plasmids. The recombinant plasmids were introduced into fresh competent cells of *E. coli* Rossetta (TaKaRa, Dalian, China), via the ClonExpress II One Step Cloning Kit (Vazyme). The resulting positive transformants were cultured in Luria-Bertani medium for 5 h at 30 °C, then for 24 h at 16 °C, after addition of 1 mM isopropyl-β-d-thiogalactopyranoside (Solarbio Life Sciences, Beijing, China). *E. coli* Rossetta cells were collected and disrupted to extract the recombinant polypeptides. The recombinant proteins were purified by ProteinIso® Ni-NTA Resin (TransGen).

The DNA probe (40 ng) was mixed with different amounts of recombinant protein (0–3 μg) in binding buffer (1 mM dithiothreitol, 0.1 mg/mL BSA, 50 mM KCl, 20 mM Tris-HCl, pH 8.0, 5% glycerol), then reacted at 28 °C for 20 min. The protein-DNA complexes were detected by 4% polyacrylamide-Tris-acetic acid-EDTA (TAE) gel electrophoresis and recorded with a Bio-Rad ChemiDoc™ MP Imaging System (Bio-Rad Laboratories, Hercules, CA, USA). For the competitive EMSA assay, DNA probes without FAM were substituted.

### Auto-docking analysis

SWISS-MODEL (https://swissmodel.expasy.org/) was used to model the protein homology of SANT1, compared with HDAC3 (pdb ID: 4A69 or SMTL ID: 4a69.1). GMQE global gave an overall model quality measurement between 0 and 1, with a higher number indicating higher expected quality.

A model of the DNA structure of *PoxGA15A*_*-558–-492*_ was constructed using the 3dRNA/DNA Web Server (http://biophy.hust.edu.cn/new/3dRNA/create), with the following parameters: molecule type, DNA; procedure, default; enter the double-stranded DNA sequence of *PoxGA15A*_*-558–-492*_ in the sequence box, enter the same length base pair format “()” in the 2D structure box; other parameters were the default values.

The HDOCK SERVER (http://hdock.phys.hust.edu.cn/) was used for molecular docking of SANT1 with *PoxGA15A*_*-558–-492*_. The *PoxGA15A*_*-558–-492*_ and SANT1 models were uploaded to the boxes of Input Receptor Molecule and Input Ligand Molecule, respectively.

PyMOL software (PyMOL Molecular Graphics System Version 2.4.1, copyright (C) Schrodinger, LLC) was used to analyze the docking results and visualize the surface structures.

### Co-IP

The intracellular proteins were extracted from *P. oxalicum* strains ∆*PoxKu70*, O*PoxRsrA*_901–1360_-*his*, O*POX_g08550*-*gfp* and O*POX_g08550-gfp*::*PoxRsrA*_901–1360_-*his*, after culture in liquid modified minimal medium containing 1% SCS for 48 h, then dissolved in PBS buffer. The protein extracts were incubated with anti-GFP antibody (TransGen) and Protein A/G agarose beads (Beaver Biomedical Engineering Co. Ltd, Suzhou, China) on ice for 4 h. The precipitated proteins were eluted by BeaverBeads™ Protein A/G immunoprecipitation Kit (Beaver), and the eluted samples were detected by anti-GFP and anti-His antibodies (TransGen Biotech) to verify the interaction by Western blotting (Fig. 5c).

### Bioinformatic software

Homologous alignment performed with MUSCLE (https://www.ebi.ac.uk/Tools/msa/muscle/). To be specific, a set of protein sequences were obtained from NCBI (https://www.ncbi.nlm.nih.gov/), and then performed alignment MUSCLE with default parameters.

Evolution analysis performed with MEGA version X^31^. The phylogenetic tree was construed based on the neighbor-joining method and Poisson correction model. The bootstrap values on branches were generated when 1000 replicates were set. The other parameters were default.

### Statistics and reproducibility

Statistical analysis of experimental data was performed with Microsoft Excel (Office 2019, Microsoft, Redmond, WA) and SPSS (IBM, Armonk, NY), using a two-tailed Student’s *t* test and one-way ANOVA. Results were generated from at least three independent experiments and reproducibility was confirmed. Data values indicate mean ± standard deviation, where *N* equals the number of independent experiments.

### Reporting summary

Further information on research design is available in the Nature Portfolio Reporting Summary linked to this article.

### Supplementary information

Supplementary materials_4revised

Description of Additional Supplementary Data

Supplementary Data 1_4revised

Supplementary Data 2_4revised

Supplementary Data 3_4revised

Reporting summary

## Data Availability

All data supporting the findings of this study have been included in the article and Supplementary information. Numerical source data for the graphs and charts were shown in Supplementary Data 2. All uncropped and unedited blots/gels were found in Supplementary Data 3. All data for gene sequences could be found in the submitted genome of *P. oxalicum* strain HP7-1 in GenBank (accession number JRVD02000002). All other data are available from the corresponding author (or other sources, as applicable) on reasonable request.
