# Supplementary material for: Regulation of fungal raw-starch-degrading enzyme production depends on transcription factor phosphorylation and recruitment of the Mediator complex
Source: Commun Biol. 2023 Oct 12;6:1032. doi: 10.1038/s42003-023-05404-x (PMC10570388; doi:10.1038/s42003-023-05404-x)
Supplement: Supplementary file 1 — Supplementary materials_4revised [file 42003_2023_5404_MOESM1_ESM.pdf]

Supplementary Information

Regulation of fungal raw-starch-degrading enzyme production depends on transcription factor phosphorylation and recruitment of the Mediator complex

Yuan-Ni Ning[1,2,3], Di Tian[1,2,3], Man-Li Tan[3], Xue-Mei Luo[3], Shuai Zhao[1,2,3]*, Jia-Xun Feng[1,2,3]*

[1]State Key Laboratory for Conservation and Utilization of Subtropical Agro-bioresources, Guangxi University, 100 Daxue Road, Nanning, Guangxi 530004, People's Republic of China.

[2]Guangxi Research Center for Microbial and Enzyme Engineering Technology, Guangxi University, 100 Daxue Road, Nanning, Guangxi 530004, People's Republic of China.

[3]College of Life Science and Technology, Guangxi University, 100 Daxue Road, Nanning, Guangxi 530004, People's Republic of China.

Content:

Supplementary Figures 1–31 and legends

Supplementary Tables 1–2

***Correspondence:** jiaxunfeng@sohu.com; shuaizhao0227@gxu.edu.cn

Tel: +86-771-323-9401

Mailing address: College of Life Science and Technology, Guangxi University,

100 Daxue Road, Nanning, Guangxi 530004, People's Republic of China

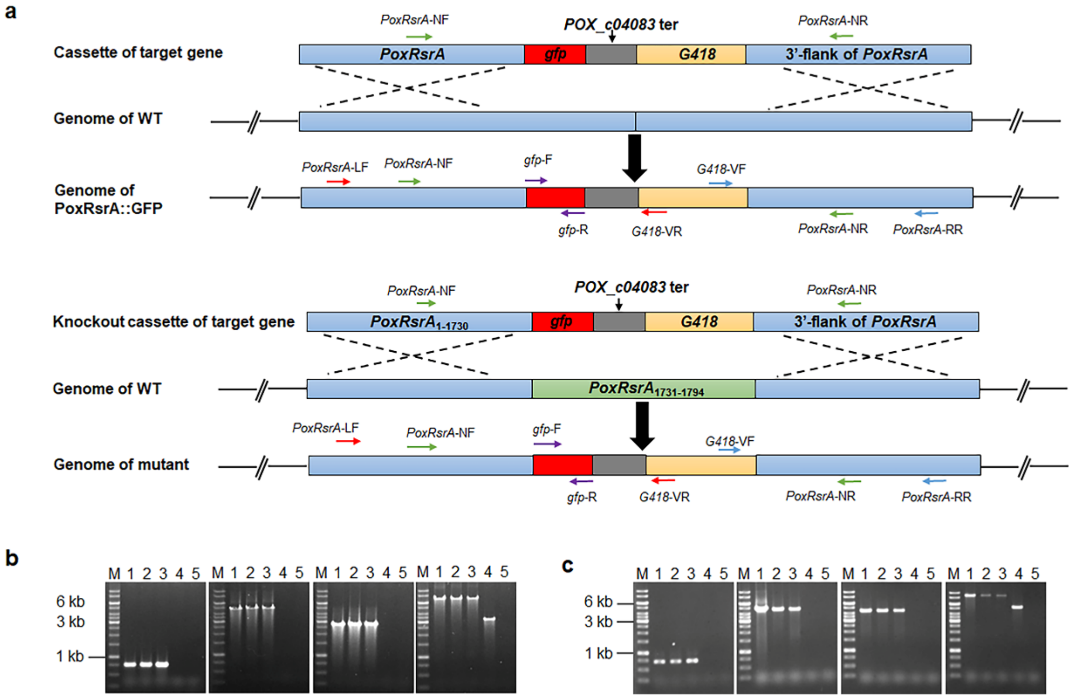

Supplementary Fig. S1. Construction of *Penicillium oxalicum* mutants PoxRsrA::GFP and Δ1731–1794::*gfp*.

a Construction strategy of the *P. oxalicum* mutants. **b** PCR confirmation of thePoxRsrA::GFP transformants. **c** PCR confirmation of the Δ1731–1794::*gfp* transformants. In (**a**), *POX_c04083* ter refers to *POX_c04083* terminator. *gfp*: gene encoding green fluorescence protein. *G418*: Geneticin resistance gene. WT: the wild-type strain. In (**b, c**), M: 1 kb DNA Marker, 1–3: three different transformants of PoxRsrA::GFP or Δ1731–1794::*gfp*, 4: WT*,* 5: ddH_2O. The panels from left to right indicate PCR production of the *gfp* gene (using primers *gfp*-F and *gfp*-R), left-cross fragment (using primers *PoxRsrA*-LF and *G418*-VR), right-cross fragment (using primers *g418*-VF and *PoxRsrA*-RR) and *PoxRsrA* expression cassette (using primers *PoxRsrA*-NF and *PoxRsrA*-NR).

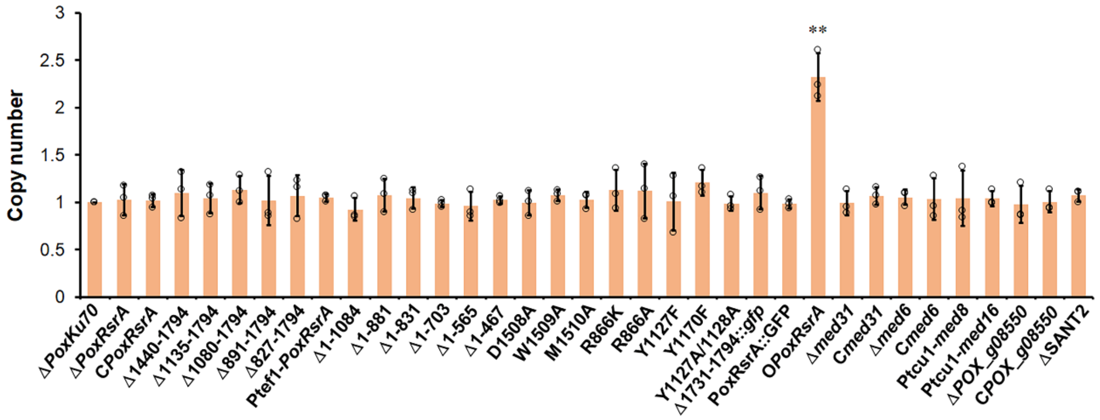

Supplementary Fig. S2. Copy number of DNA cassette the construct-of-interest integrated into genome of *P. oxalicum* strains in this study.

Total DNAs from all tested *P. oxalicum* strains are extracted, then used as the template for quantitative PCR, and WT is used as control. The actin gene is used as internal reference. Copy number is calculated based on the $2^{-\Delta\Delta Ct}$. ***p* < 0.01 indicates significant differences between mutant and WT. WT: the wild-type strain.

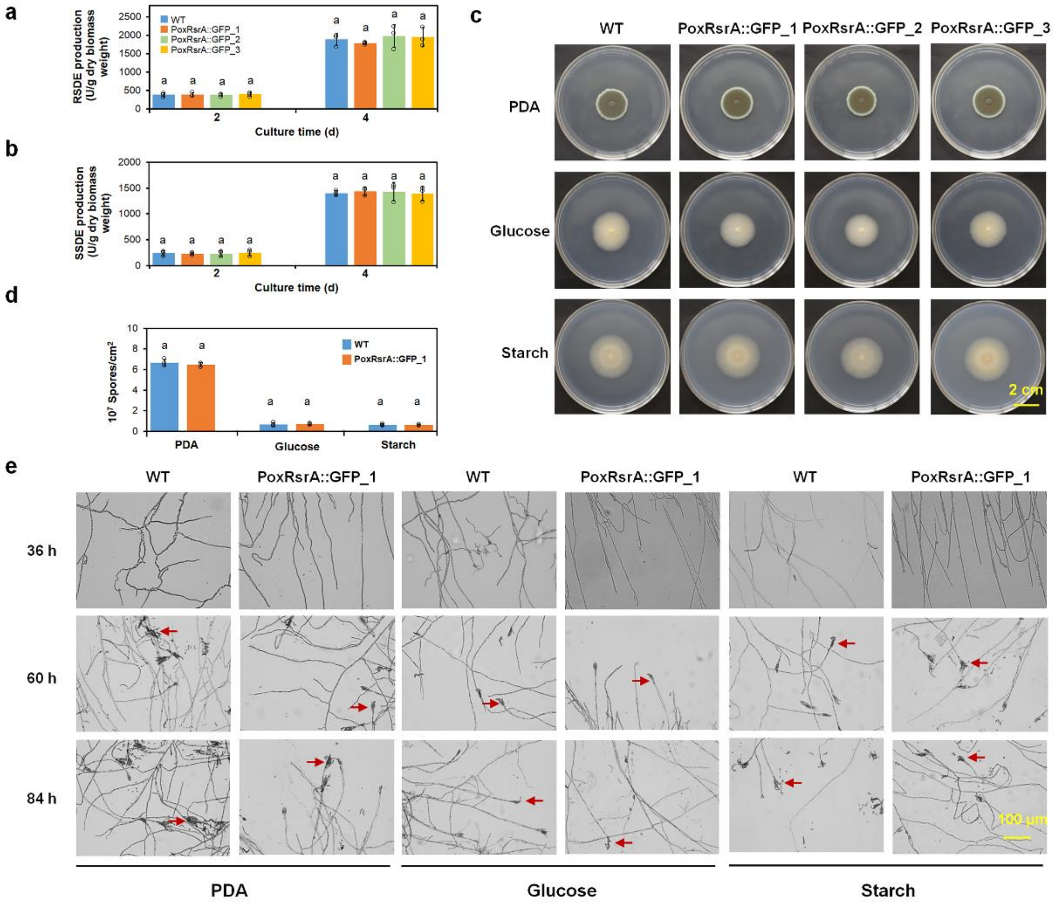

Supplementary Fig. S3. Amylase production and phenotypic analysis of *P. oxalicum* mutant PoxRsrA::GFP.

a RSDE production. **b** SSDE production. In (**a, b**), *P. oxalicum* strains are pre-grew in glucose medium for 24 h, then transferred into medium containing soluble corn starch and cultured for 2–4 days. Data values are mean ± standard deviation. RSDE: raw-starch-degrading enzyme. SSDE: soluble-starch-degrading enzyme. **c** Colony phenotypes on solid plates. **d** Spore production**.** In (**c, d**), fungal strains are cultured on solid plates containing different carbon sources for 5 days. PDA: potato dextrin agar. In panels a, b & d, values marked with same lower letters indicate no statistically significant difference ($p > 0.05$) between each group according to Student's two-tail *t*-test. **e** Observation of

mycelia. The mycelia are harvested when cultured on solid plates containing different carbon sources for 36–84 h. The mycelia are observed with a light microscope (OLYMPUS DP480, Tokyo, Japan). The red arrows indicate conidiospores. GFP: green fluorescence protein.

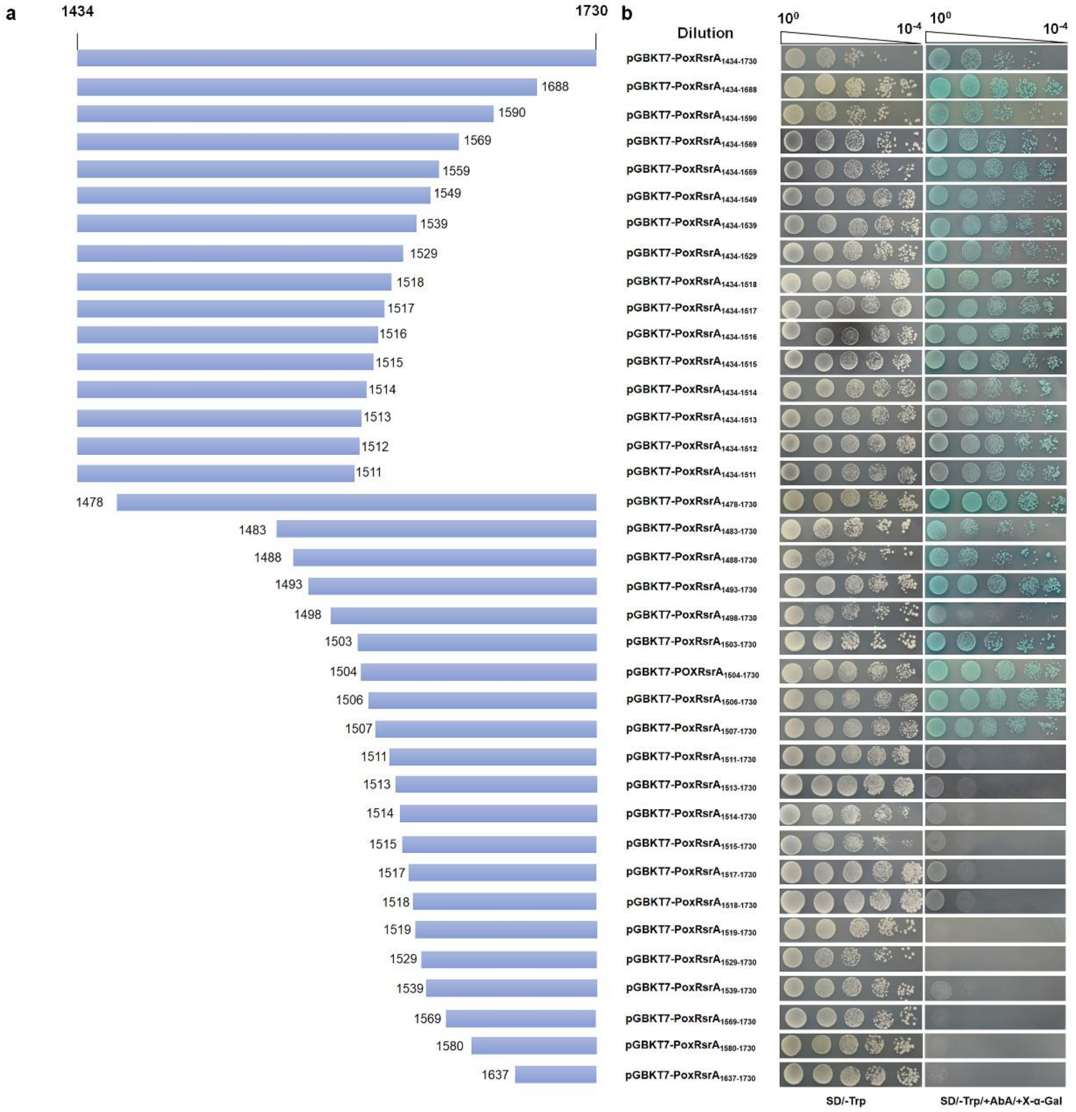

Supplementary Fig. S4. Determination of transcription-activation domain in PoxRsrA.

a Schematic diagram indicating truncation of PoxRsrA. **b** Transcription-activation assays in yeast**.** Yeast cells were grown on SD-Trp and SD/-Trp/+AbA/+X-α-Gal for 4 days. The original concentration of each yeast sample was adjusted to OD_{600} = 1.0.

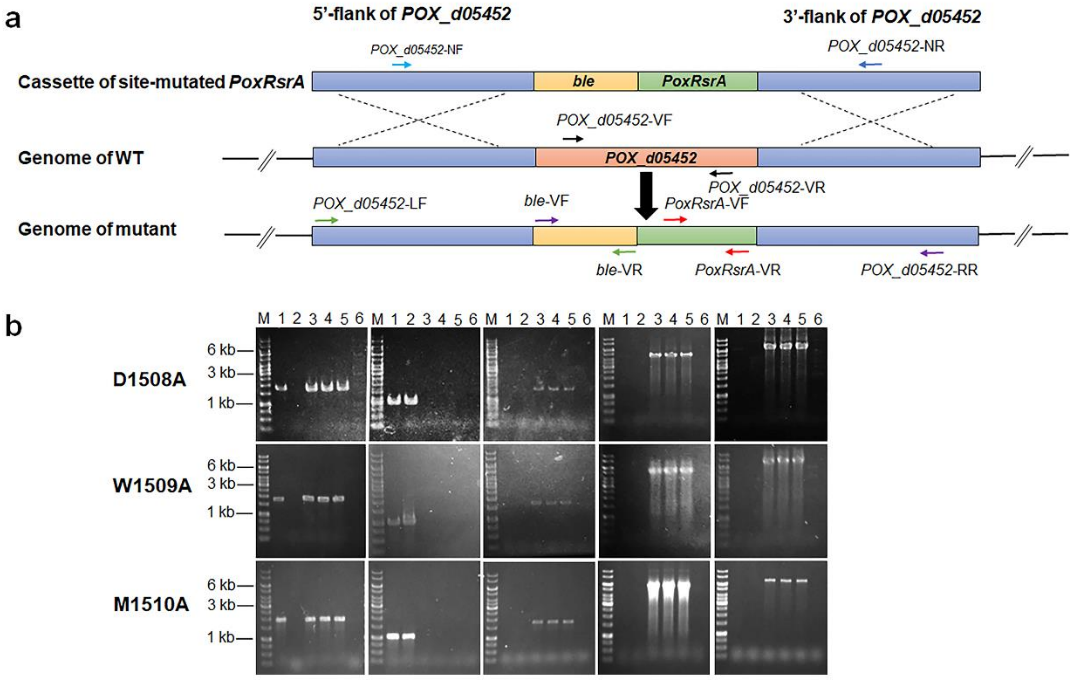

Supplementary Fig. S5. Construction of *P. oxalicum* mutants D1508A, W1509A and M1510A.

a Construction strategy of the *P. oxalicum* mutants. **b** PCR confirmation. In (**b**), M: 1 kb DNA Marker, 1: Δ*PoxKu70*, 2: Δ*PoxRsrA*, 3: Mutant-1, 4: Mutant-2, 5: Mutant-3, 6: ddH_2O. The panels from left to right indicate PCR production of *PoxRsrA* (using primers *PoxRsrA*-VF and *PoxRsrA*-VR), *POX_d05452* (using primers *POX_d05452*-VF and *POX_d05452*-VR), the bleomycin resistance gene (using primers *ble*-VF and *ble*-VR), left-cross fragment (using primers *POX_d05452*-LF and *ble*-VR) and right-cross fragment (using primers *PoxRsrA*-VF and *POX_d05452*-RR). *ble*: Bleomycin resistance gene.

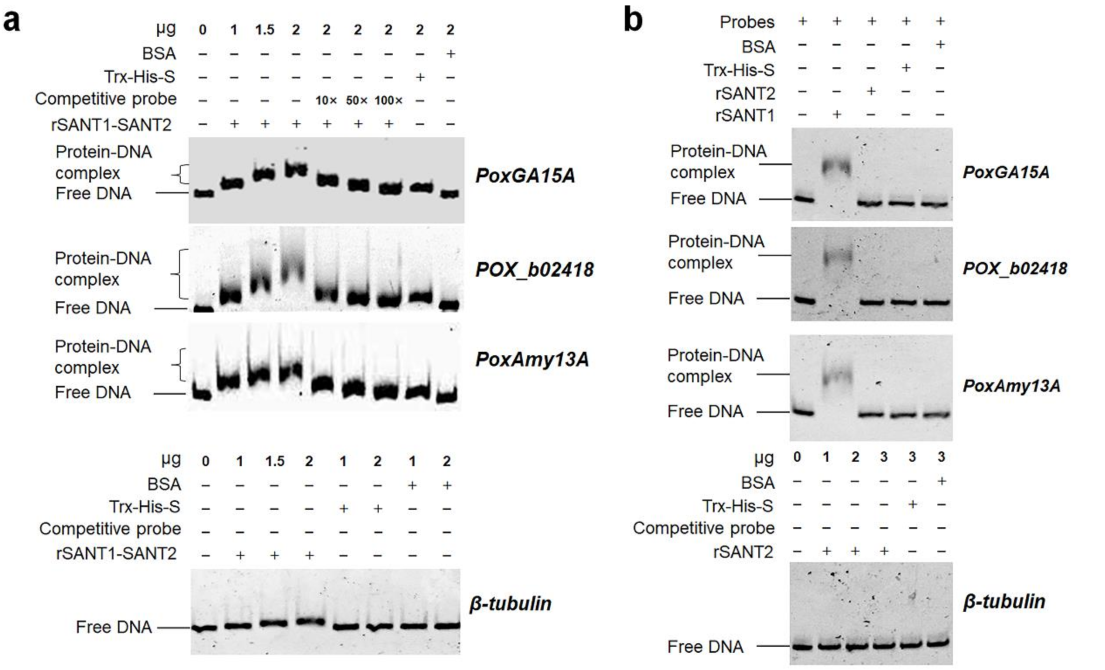

Supplementary Fig. S6. In vitro electrophoretic mobility shift assay (EMSA) of the recombinant proteins and the promoter regions of the genes encoding major amylases.

a EMSA showing the interaction between the rSANT1-SANT2 and probes. **b** EMSA for testing the interaction between the rSANT2 and probes. Each reaction comprises of Trx-His-S-tagged rSANT1-SANT2 (0–2 µg) or rSANT1/ rSANT1 (2 µg) and 6-carboxyfluorescein-tagged probe (~50 ng). The Trx-His-S peptide and bovine serum albumen (BSA) were used as controls, as well as the promoter region of gene encoding β-tubulin. Competitive probes were DNA fragments without 6-carboxyfluorescein. *PoxGA15A*: raw-starch-degrading glucoamylase gene. *POX_b02418*: glucoamylase gene. *PoxAmy13A*: α-amylase gene.

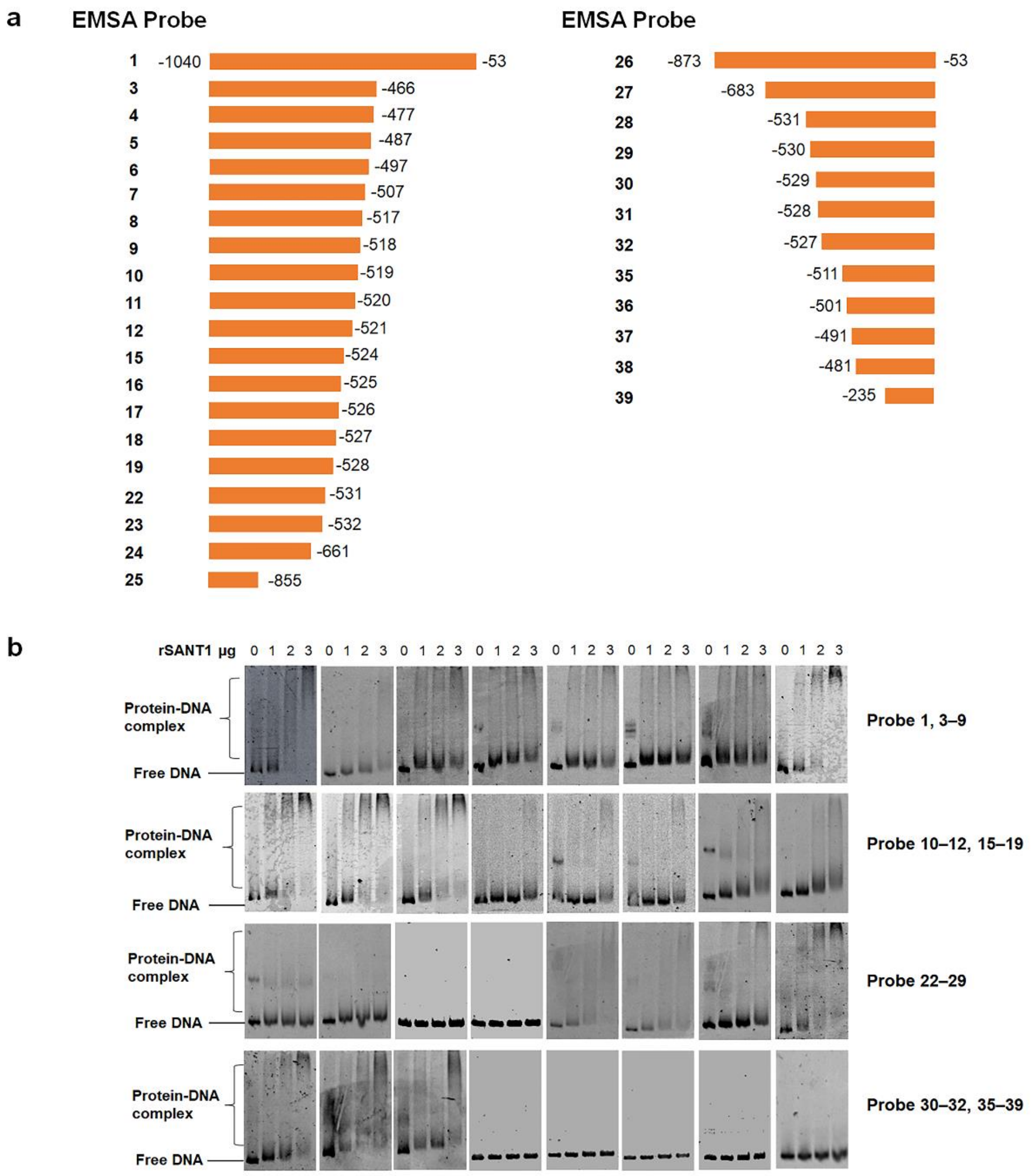

Supplementary Fig. S7. In vitro electrophoretic mobility shift assay (EMSA) for testing interaction between rSANT1 and the truncated probes.

a Schematic diagram indicating truncation of the promoter region of raw-starch-degrading glucoamylase gene *PoxGA15A*. **b** EMSA for testing the interaction between rSANT1 and the truncated probes from (**a**). Each reaction comprises of Trx-His-S-tagged rSANT1 (0–3 μg) and 6-carboxyfluorescein-tagged probe (~50 ng).

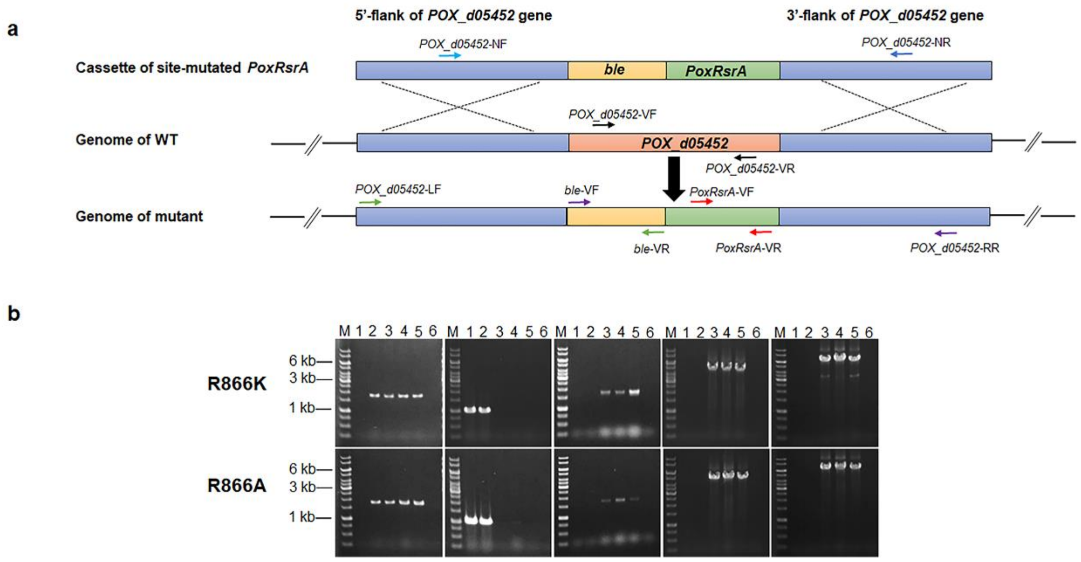

Supplementary Fig. S8. Construction of the *P. oxalicum* mutants R866K and R866A.

a Construction strategy of the *P. oxalicum* mutants**. b** PCR confirmation**.** In (**b**), M: 1 kb DNA Marker, 1: Δ*PoxRsrA*, 2: WT, 3: R866K-1 or R866A-1, 4: R866K-2 or R866A-2*,* 5: R866K-3 or R866A-3, 6: ddH$_2$O. The panels from left to right indicate PCR production of mutated *PoxRsrA* (using primers *PoxRsrA*-VF and *PoxRsrA*-VR), *POX_d05452* (using primers *POX_d05452*-VF and *POX_d05452*-VR), bleomycin resistance gene (using primers *ble*-VF and *ble*-VR), left-cross fragment (using primers *POX_d05452*-LF and *ble*-VR) and right-cross fragment (using primers *PoxRsrA*-VF and *POX_d05452*-RR). WT: the wild-type strain. *ble*: Bleomycin resistance gene.

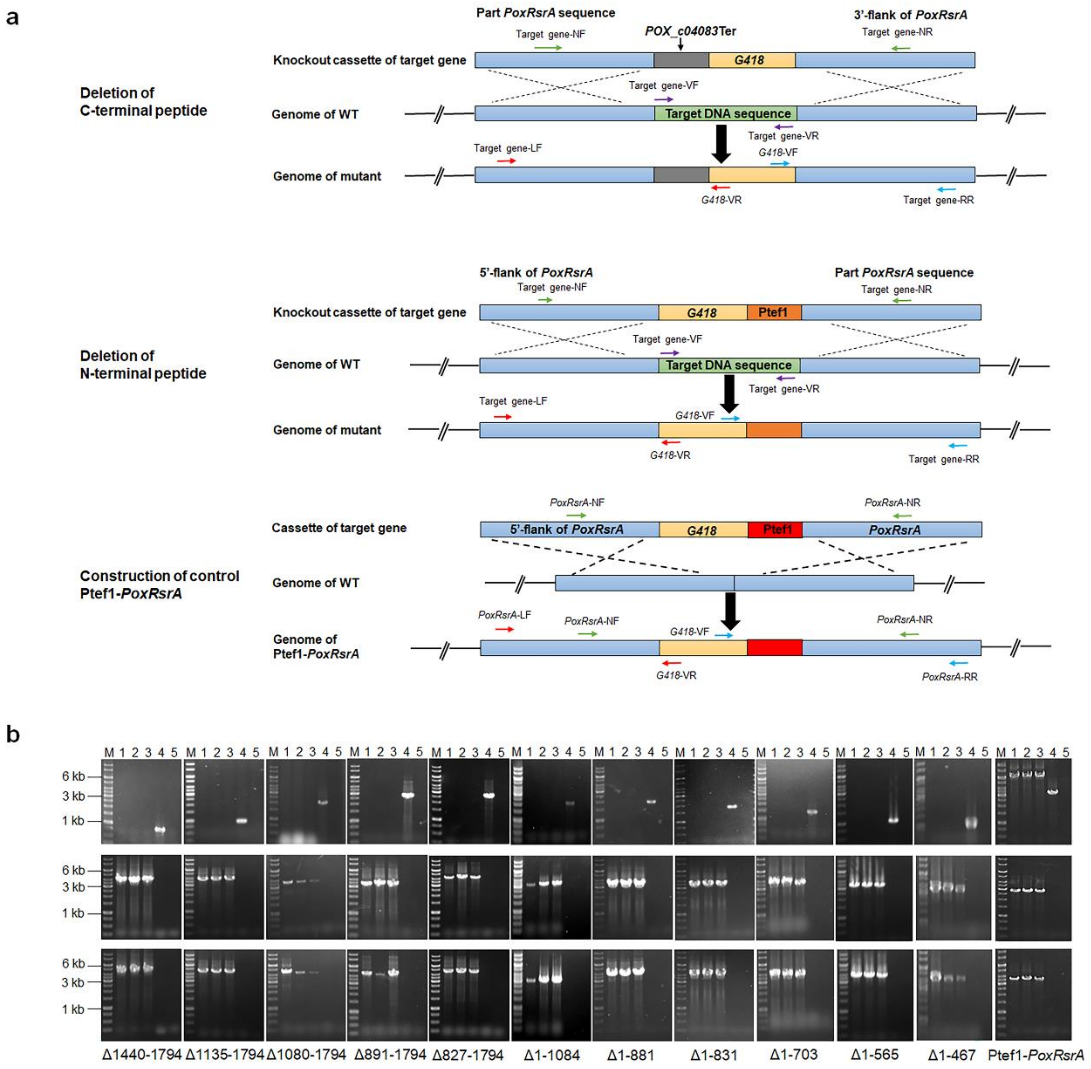

Supplementary Fig. S9. Construction of the *P. oxalicum* mutants with different lengths of PoxRsrA peptides deleted.

a Construction strategy of the *P. oxalicum* mutants. **b** PCR confirmation. *POX_c04083* ter and Ptef1 refer to the *POX_c04083* terminator and the promoter of translation elongation factor eEF1a (Tef1) gene, respectively. *G418*: Geneticin resistance gene. WT: the wild-type strain. In panel b, M: 1 kb DNA Marker, 1: Mutant-1, 2: Mutant-2, 3: Mutant-3, 4: WT*,* 5: ddH$_2$O. The panels from top to bottom indicate PCR production of the target gene (using primers target gene-VF and target gene-VR), left-cross fragment (using primers target

gene-LF and *G418*-VR), and right-cross fragment (using primers *G418*-VF and target gene-RR).

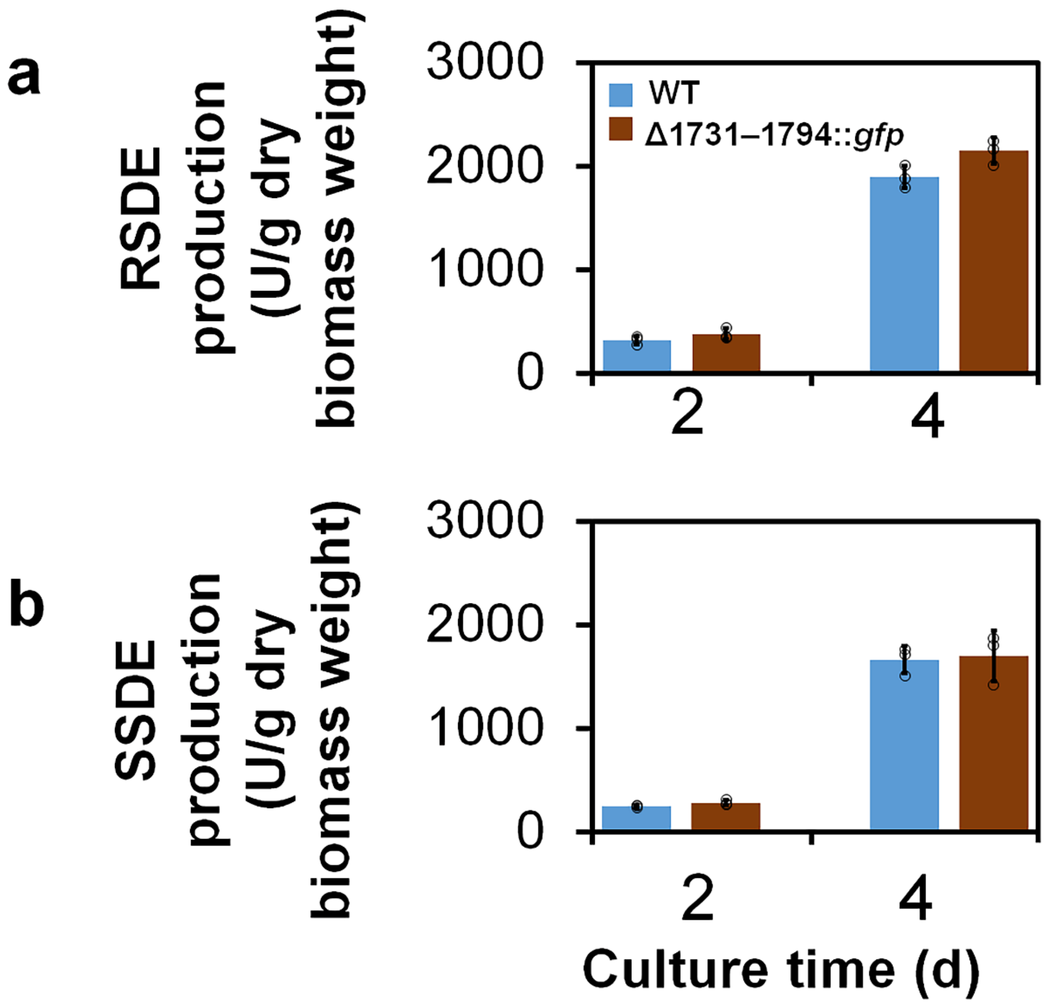

Supplementary Fig. S10. Effects of oligopeptide PoxRsrA$_{1731–1794}$ on amylase production by *P. oxalicum*.

a RSDE production. **b** SSDE production. *P. oxalicum* mutant Δ1731–1794::*gfp* and wild-type strain (WT) pre-grew in glucose medium for 24 h, then transferred into medium containing soluble corn starch and cultured for 2–4 days. Data values are mean ± standard deviation. RSDE: raw-starch-degrading enzyme.

SSDE: soluble-starch-degrading enzyme.

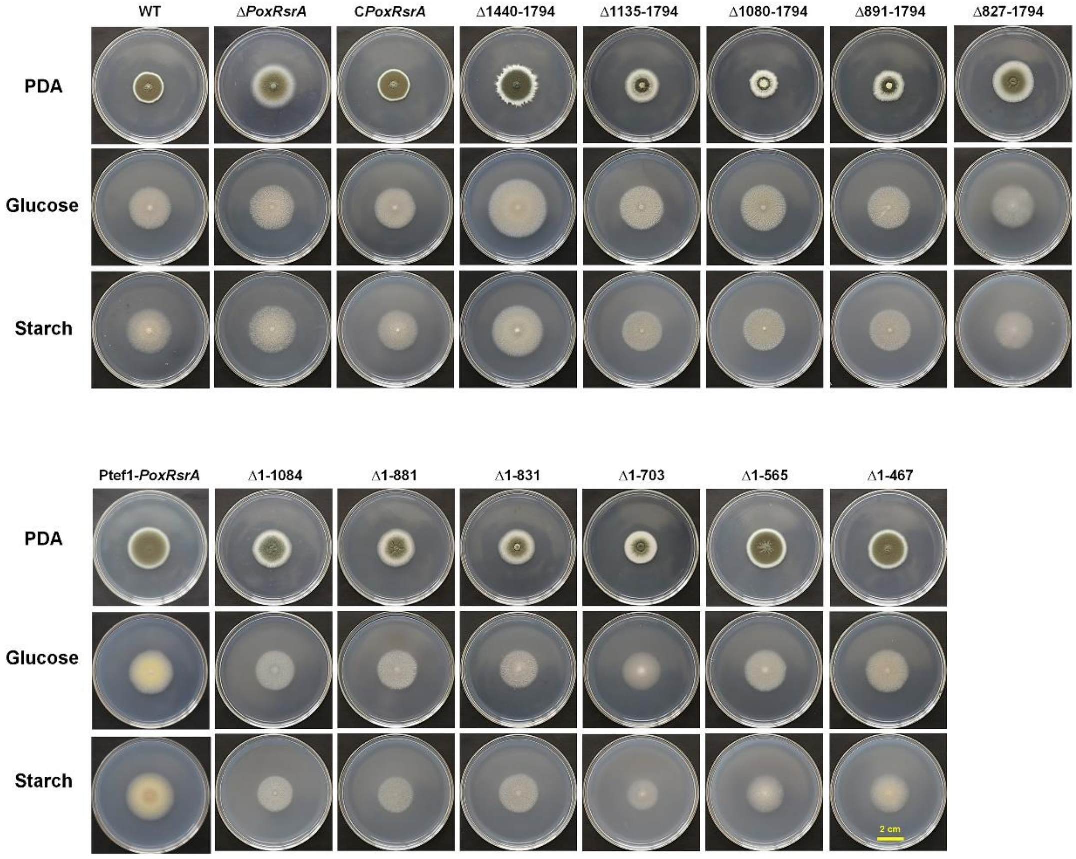

Supplementary Fig. S11. Colony observation of *P. oxalicum* mutants with different lengths of PoxRsrA peptides deleted. All tested strains are cultured on solid plates containing different carbon sources for 5 days. The wild-type strain (WT), mutant Δ*PoxRsrA* and complementation strain C*PoxRsrA* are used as controls. PDA: potato dextrose agar.

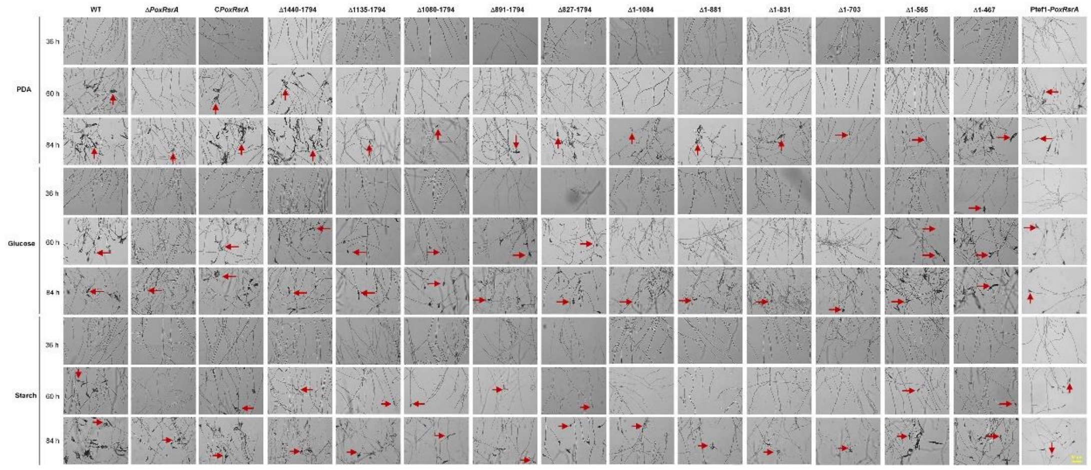

Supplementary Fig. S12. Observation of mycelial development of *P. oxalicum* mutants with different lengths of PoxRsrA peptides deleted. All tested strains are cultured on solid plates containing different carbon sources for 36–84 h. The wild-type strain (WT), mutant Δ*PoxRsrA* and complementation strain C*PoxRsrA* are used as controls. PDA: potato dextrose agar. The red arrows indicate conidiospores. Scale bar = 50 μm.

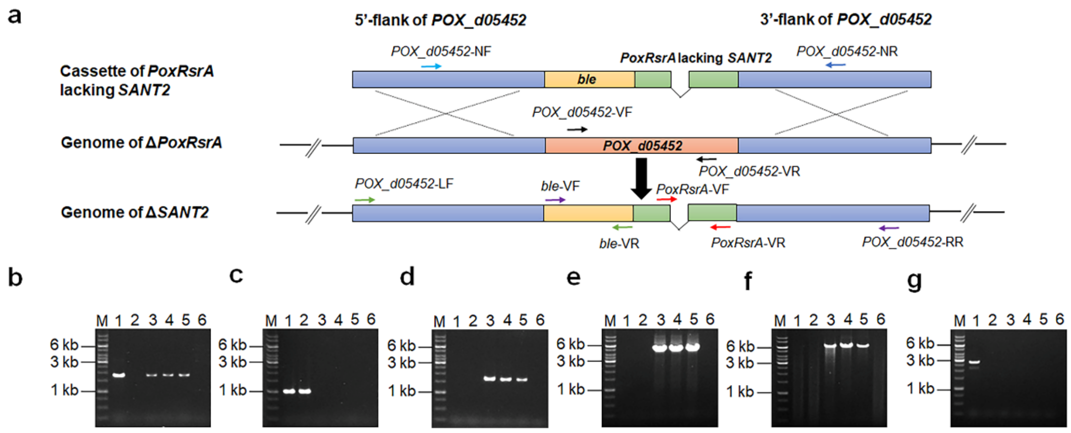

Supplementary Fig. S13. Construction of *P. oxalicum* mutant Δ*SANT2*.

a Construction strategy of *P. oxalicum* mutant. **b** PCR production of *PoxRsrA* lacking *SANT2* (using primers *PoxRsrA*-VF and *PoxRsrA*-VR). **c** PCR production of *POX_d05452* (using primers *POX_d05452*-VF and *POX_d05452*-VR). **d** PCR production of *ble* resistance gene (using primers *ble*-VF and *ble*-VR). **e** PCR production of left-cross fragment (using primers *POX_d05452*-LF and *ble*-VR). **f** PCR production of right-cross fragment (using primers *PoxRsrA*-VF and *POX_d05452*-RR). **g** PCR production of *SANT2* (using primers *SANT2*-VF and *PoxRsrA*-VR). In (**b–g**), M: 1 kb DNA Marker, 1: the wild-type strain Δ*PoxKu70*, 2: Δ*PoxRsrA*, 3–5: three different transformants of Δ*SANT2*, 6: ddH_2O. *ble*: Bleomycin resistance gene.

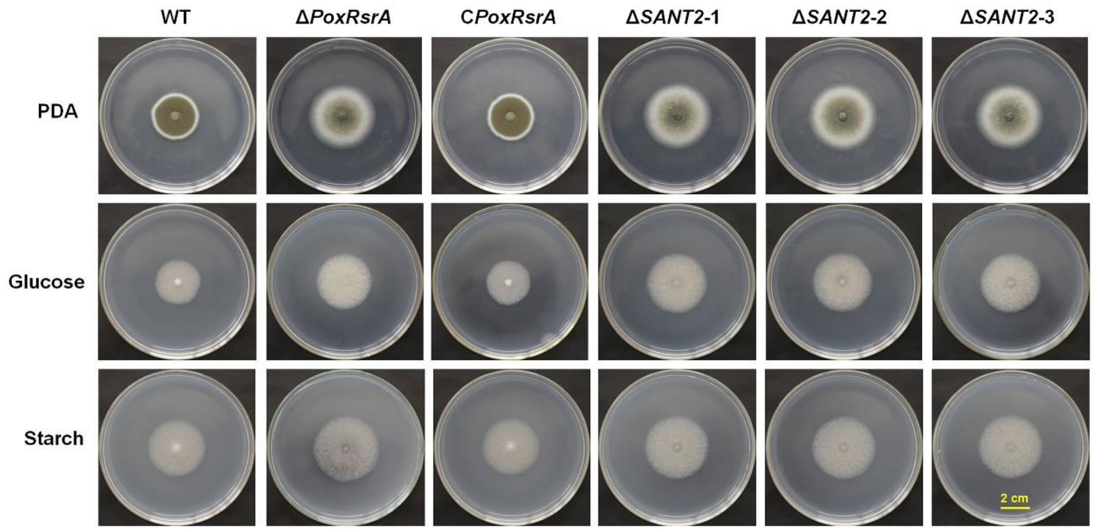

Supplementary Fig. S14. Colony observation of *P. oxalicum* mutant Δ*SANT2*. All tested strains are cultured on solid plates containing different carbon sources for 5 d. The wild-type strain (WT), mutant Δ*PoxRsrA* and complementation strain C*PoxRsrA* are used as controls. PDA: potato dextrose agar.

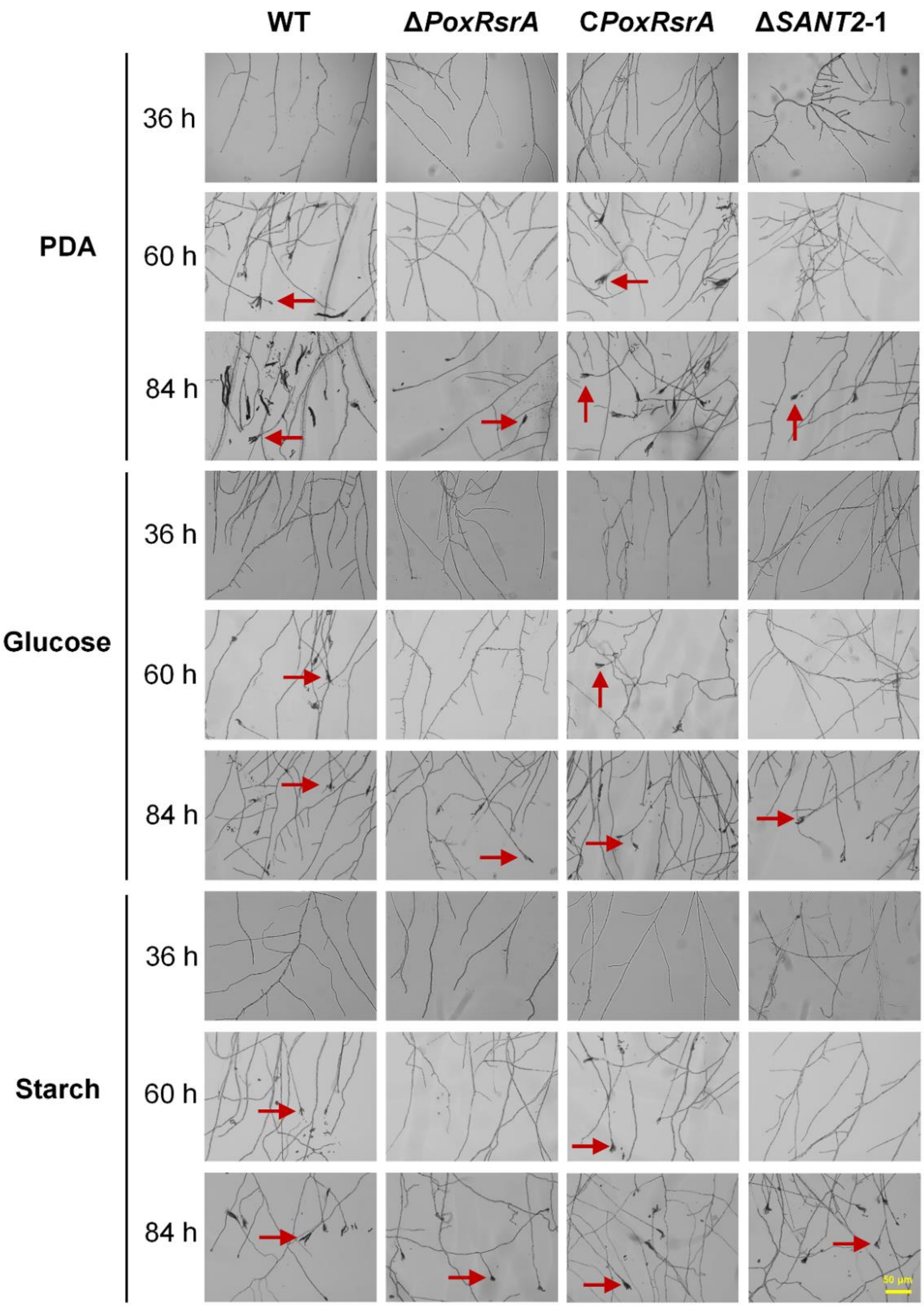

Supplementary Fig. S15. Observation of mycelial development of *P. oxalicum* mutant Δ*SANT2*. All tested strains are cultured on solid plates containing different carbon sources for 36–84 h. The wild-type strain (WT), mutant Δ*PoxRsrA* and complementation strain C*PoxRsrA* are used as controls. PDA: potato dextrose agar. The red arrows indicate conidiospores.

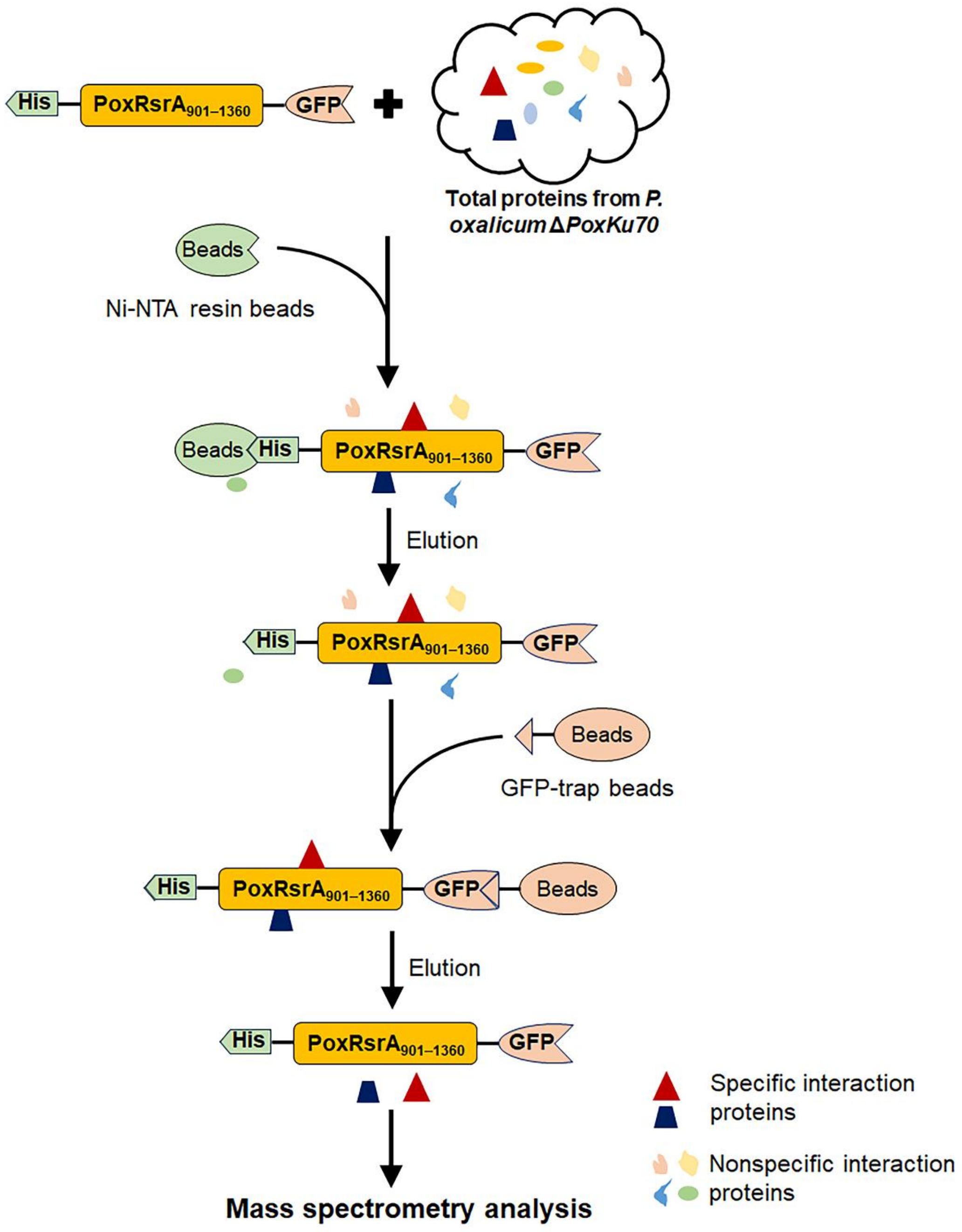

Supplementary Fig. 16. Schematic illustration indicating principle for tandem affinity purification-mass spectroscopy analysis. GFP: green fluorescence protein.

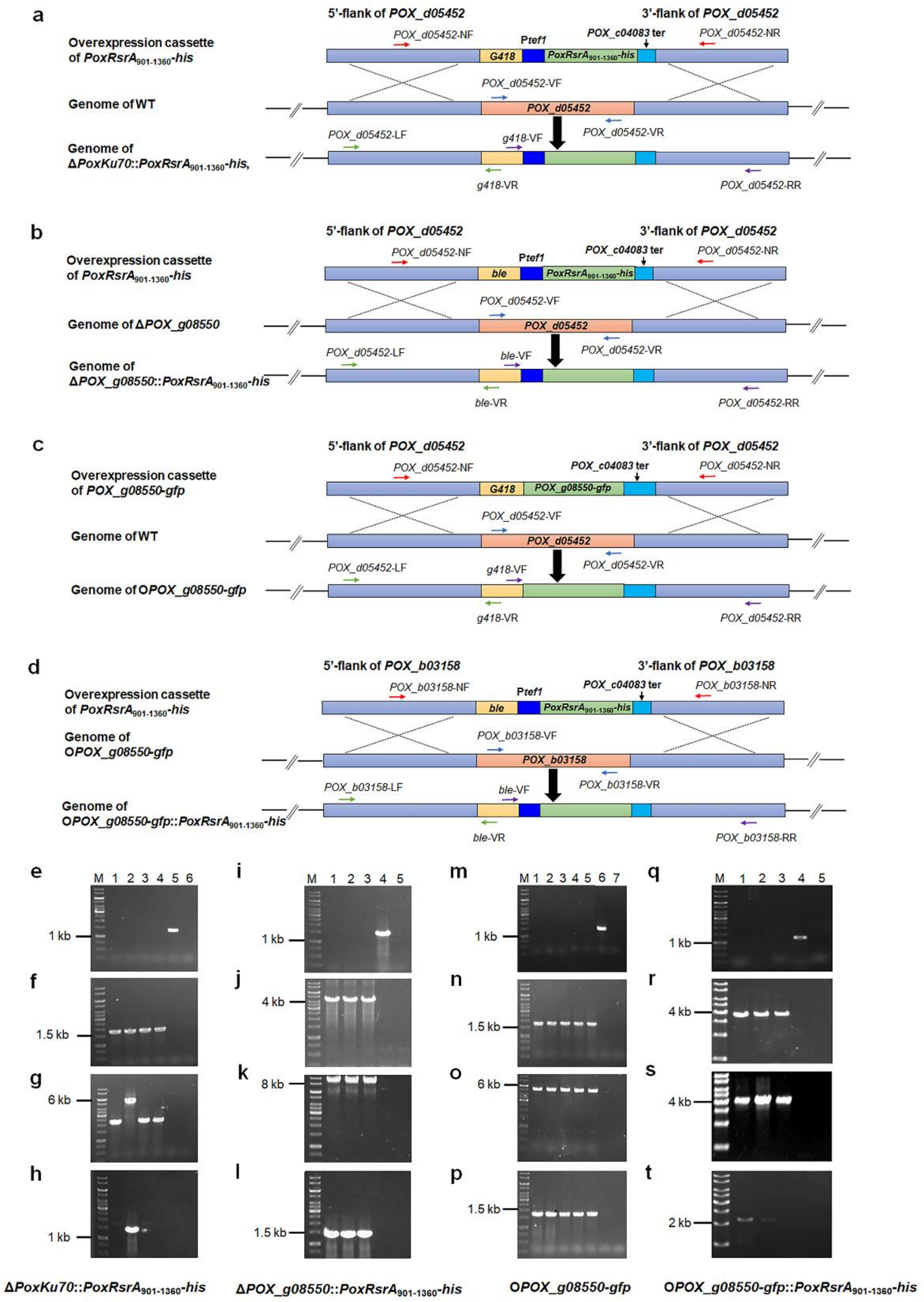

Supplementary Fig. S17. Construction of *P. oxalicum* mutants.

a Construction strategy of the mutant Δ*PoxKu70*::*PoxRsrA*$_{901-1360}$-*his*. **b** Construction strategy of the mutant Δ*POX_g08550*::*PoxRsrA*$_{901-1360}$-*his*. **c** Construction strategy of the mutant O*POX_g08550-gfp*. **d** Construction

strategy of the mutant O*POX_g08550-gfp*::*PoxRsrA*$_{901\text{-}1360}$-*his*. **e–h** PCR confirmation of the mutant Δ*PoxKu70*::*PoxRsrA*$_{901\text{-}1360}$-*his*. **i–l** PCR confirmation of the mutant Δ*POX_g08550*::*PoxRsrA*$_{901\text{-}1360}$-*his*. **m–p** PCR confirmation of O*POX_g08550-gfp*. **q–t** PCR confirmation of mutant O*POX_g08550-gfp*::*PoxRsrA*$_{901\text{-}1360}$-*his*. **e** PCR production of *POX_d05452* (using primers *POX_d05452*-VF and *POX_d05452*-VR). **f** PCR production of the left-cross fragment (using primers *POX_d05452*-LF and *g418*-VR). **g** PCR production of the right-cross fragment (using primers *g418*-VF and *POX_d05452*-RR). **h** PCR production of *g418*VF_*PoxRsrA*$_{901\text{-}1360}$-*his* (using primers *g418*-VF and *PoxRsrA*-VR). M: 1 kb DNA Marker, 1–4: four different transformants of Δ*PoxKu70*::*PoxRsrA*$_{901\text{-}1360}$, 5: Δ*PoxKu70*, 6: ddH_2O. **i** PCR production of *POX_d05452* (using primers *POX_d05452*-VF and *POX_d05452*-VR). **j** PCR production of the left-cross fragment (using primers *POX_d05452*-LF and *ble*-VR). **k** PCR production of the right-cross fragment (using primers *ble*-VF and *POX_d05452*-RR). **l** PCR production of *PoxRsrA*$_{901\text{-}1360}$-*his* (using primers *ble*-VF and *PoxRsrA*-VR). M: 1 kb Marker, 1–3: three different transformants of Δ*POX_g08550*::*PoxRsrA*$_{901\text{-}1360}$-*his*, 4: Δ*PoxKu70*, 5: ddH_2O. **m** PCR production of *POX_d05452* (using primers *POX_d05452*-VF and *POX_d05452*-VR). **n** PCR production of the left-cross fragment (using primers *POX_d05452*-LF and *G418*-VR). **o** PCR production of the right-cross fragment (using primers *G418*-VF and *POX_d05452*-RR). **p** PCR production of *POX_g08550-gfp* (using primers *POX_g08550*P-F and *gfp*-R). M: 1 kb DNA

Marker, 1–5: five different transformants of O*POX_g08550-gfp*, 6: Δ*PoxKu70*, 7: ddH_2O. **q** PCR production of *POX_b03158* (using primers *POX_b03158*-VF and *POX_b03158*-VR). **r** PCR production of the left-cross fragment (using primers *POX_b03158*-LF and *ble*-VR). **s** PCR production of the right-cross fragment (using primers *ble*-VF and *POX_b03158*-RR). **t** PCR production of *ble-PoxRsrA*$_{901\text{-}1360}$ (using primers *ble*-VF and *PoxRsrA*-VR). M: 1 kb DNA Marker, 1–3: three different transformants of O*POX_g08550-gfp*::*PoxRsrA*$_{901\text{-}1360}$-*his*, 4: O*POX_g08550-gfp*, 5: ddH_2O. *POX_c04083* ter refers to *POX_c04083* terminator. *gfp*: gene encoding green fluorescence protein. *G418*: Geneticin resistance gene. *ble*: bleomycin resistance gene. WT: the wild-type strain. Genes *POX_d05452* and *POX_b03158* respectively encode the aspartic peptidase PepA and PepB.

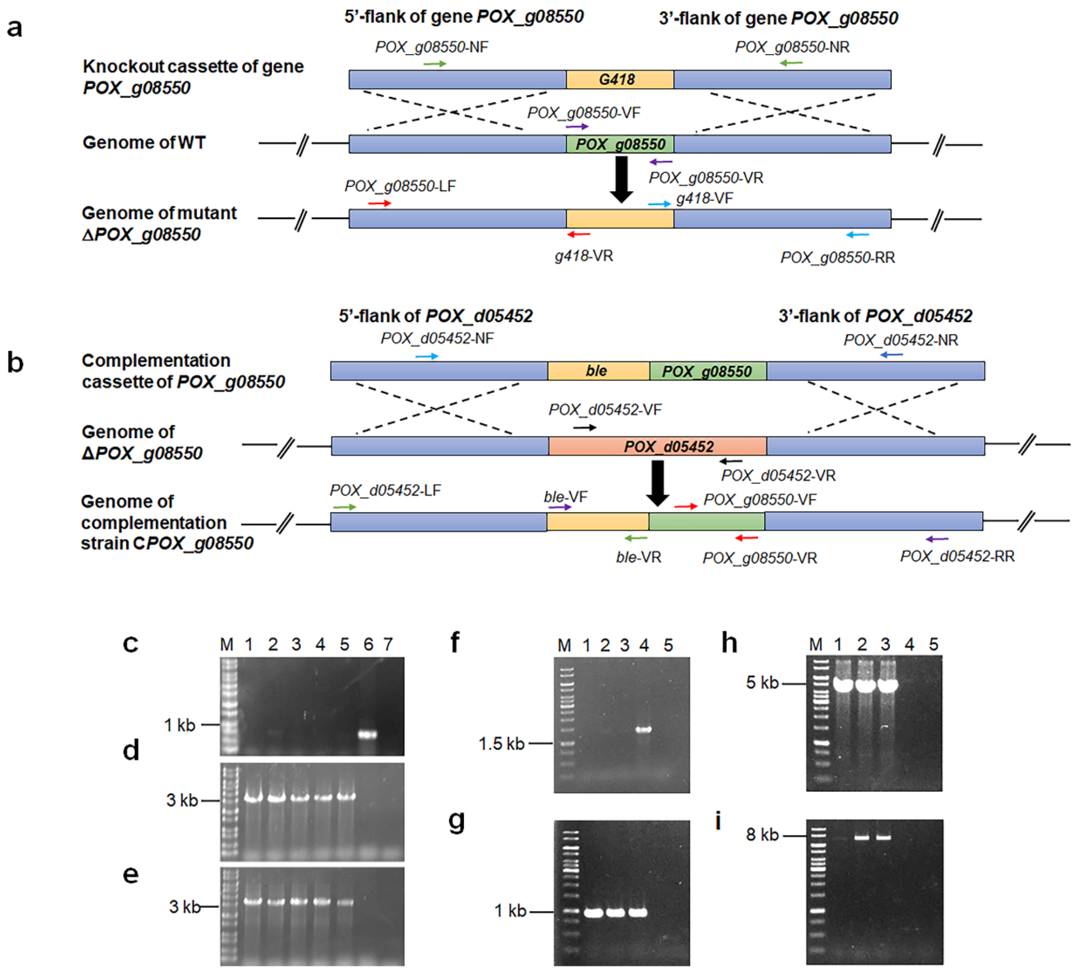

Supplementary Fig. S18. Construction of *P. oxalicum* mutants Δ*POX_g08550* and C*POX_g08550*.

a Construction strategy of the mutant Δ*POX_g08550*. **b** Construction strategy of the mutant C*POX_g08550*. **c–e** PCR confirmation of the mutant Δ*POX_g08550*. **c** PCR production of *POX_g08550* (using primers *POX_g08550*-VF and *POX_g08550*-VR). **d** PCR production of left-cross fragment (using primers *POX_g08550*-LF and *G418*-VR). **e** PCR production of right-cross fragment (using primers *G418*-VF and *POX_g08550*-RR). M: 1 kb DNA Marker, 1–5: five different transformants of Δ*POX_g08550*, 6: WT, 7: ddH$_2$O. **f–i** PCR confirmation of the mutant C*POX_g08550*. **f** PCR production

of *POX_d05452* (using primers *POX_d05452*-VF and *POX_d05452*-VR). **g** PCR production of *POX_g08550* (using primers *POX_g08550*-VF and *POX_g08550*-VR). **h** PCR production of the left-cross fragment (using primers *POX_d05452*-LF and *ble*-VR). **i** PCR production of the right-cross fragment (using primers *ble* -VF and *POX_d05452*-RR). M: 1 kb DNA Marker, 1–3: three different transformants of C*POX_g08550*, 4: WT, 5: ddH_2O. *G418*: Geneticin resistance gene. *ble*: bleomycin resistance gene. WT: the wild-type strain.

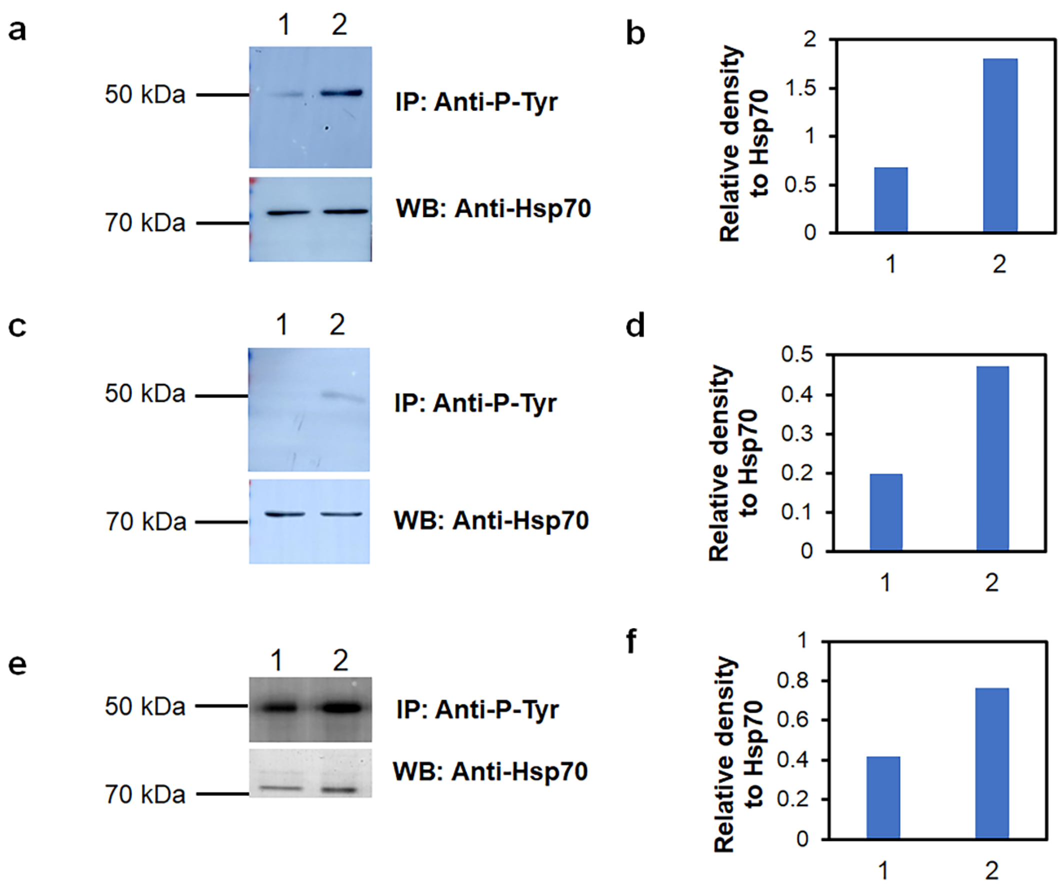

Supplementary Fig. S19. Determination of phosphorylation level at tyrosine (Tyr; Y) in PoxRsrA$_{901\text{-}1360}$.

a, c, e Western blotting (WB) showing phosphorylation level at tyrosine in PoxRsrA$_{901\text{-}1360}$. **b, d, f** Relative density of bands corresponding to the phosphorylated PoxRsrA$_{901\text{-}1360}$ that is normalized to that of the Hsp70. 1: Δ*PoxKu70*::*PoxRsrA*$_{901\text{-}1360}$-*his*; 2: Δ*POX_g08550*::*PoxRsrA*$_{901\text{-}1360}$-*his*. The phosphorylated protein is enriched by anti-His antibody, then detected with anti-P-Tyr antibody. The heat shock protein Hsp70 is used as control. IP: Immunoprecipitation.

>POX01907$_{901\text{-}1360}$

RSSRPKSNALIADLGVVKPDFDGEEEQPPPVTDTGRPRRAAAPTFGDSNDS

EGAVAGSRRGQSVKDGELTEKPAVRRGGRATG**T**R**T**QRRGAKVVQQDPKSQ

PSTPQGSNTPVPPAPKIESGIDALVEVALPPEKELVEKEPLPSASRPKAGRGR

AKDGIYVFESTETDPTTATPKPSESGYGSLQPTSYWSVPEQRDFPRLLAHFG

RDFEGISSFMKTKTTVMVKN**YY**QRRLDSGQKDFEEIVLVAEEKKARGEP**T**GP

LPVP**S**VAPKRR**Y**EATPSAIIPRPLAPHGESVSESEDARFAPK**S**KPAV**TS**PQTV

PVHSRPVVEGDRTSSRYAPLAQASTPGANSPMAATYGDDAARGSRTQPPP

SRM**S**GPRIGFF**T**EDRRDSPILQSGAQRASDMPISARHVATGAMPQEMTGME

PIALQTYMPGQPSSSLMQTSHSRHNSLTQPPSSPAARPRTELEHPSYH

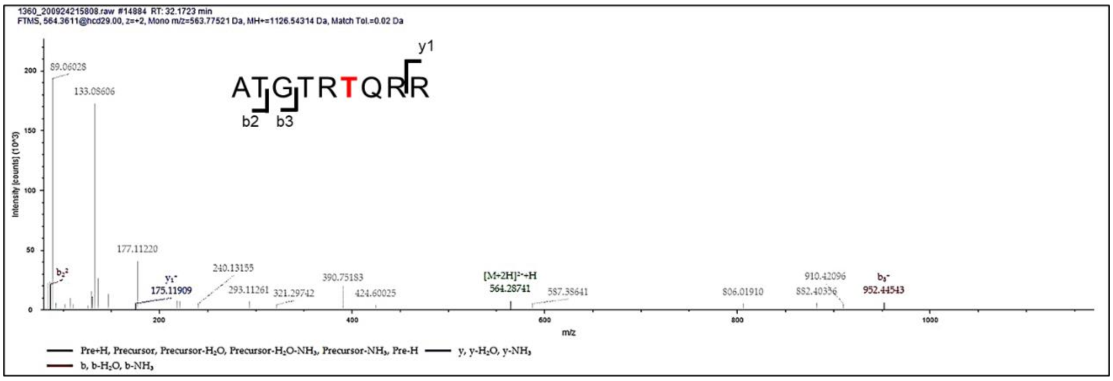

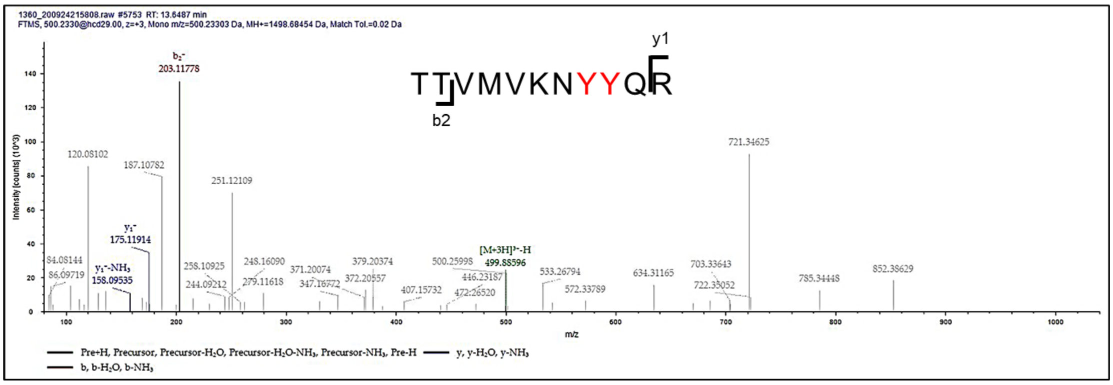

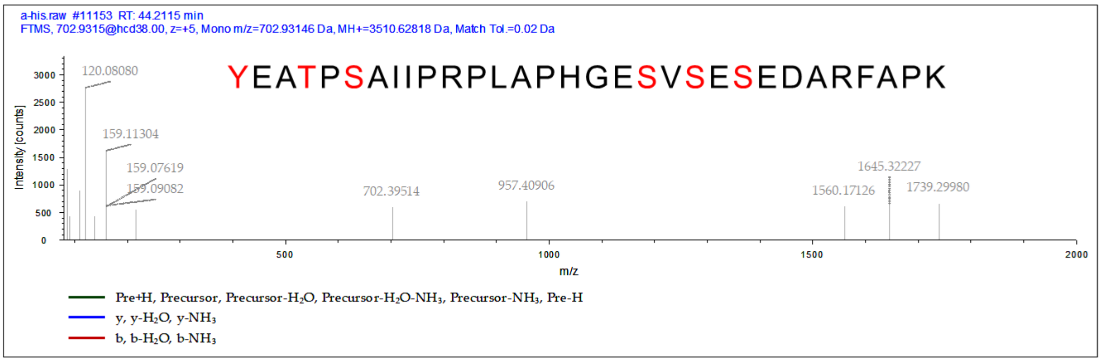
a-his.raw #11153 RT: 44.2115 min
FTMS, 702.9315@hcd38.00, z=+5, Mono m/z=702.93146 Da, MH+=3510.62818 Da, Match Tol.=0.02 Da
YEATPSAIIPRPLAPHGESVSESEDARFAPK
Intensity [counts]
m/z
120.08080
159.11304
159.07619
159.09082
702.39514
957.40906
1560.17126
1645.32227
1739.29980
Pre+H, Precursor, Precursor-H2O, Precursor-H2O-NH3, Precursor-NH3, Pre-H
y, y-H2O, y-NH3
b, b-H2O, b-NH3

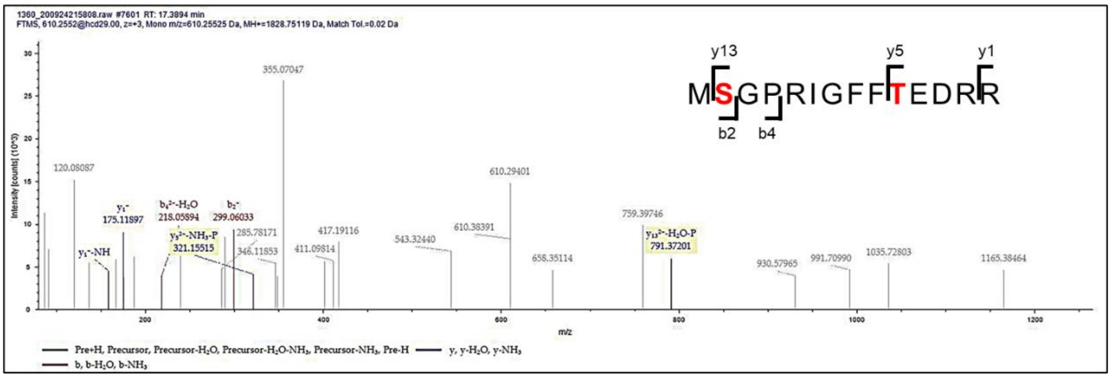
1360_200924215808.raw #7601 RT: 17.3894 min
FTMS, 610.2552@hcd29.00, z=+3, Mono m/z=610.25525 Da, MH+=1828.75119 Da, Match Tol.=0.02 Da
y13 y5 y1
MSGPRIGFFTEDRR
b2 b4
m/z
355.07047
120.08087
610.29401
759.39746
Pre+H, Precursor, Precursor-H2O, Precursor-H2O-NH3, Precursor-NH3, Pre-H
y, y-H2O, y-NH3
b, b-H2O, b-NH3

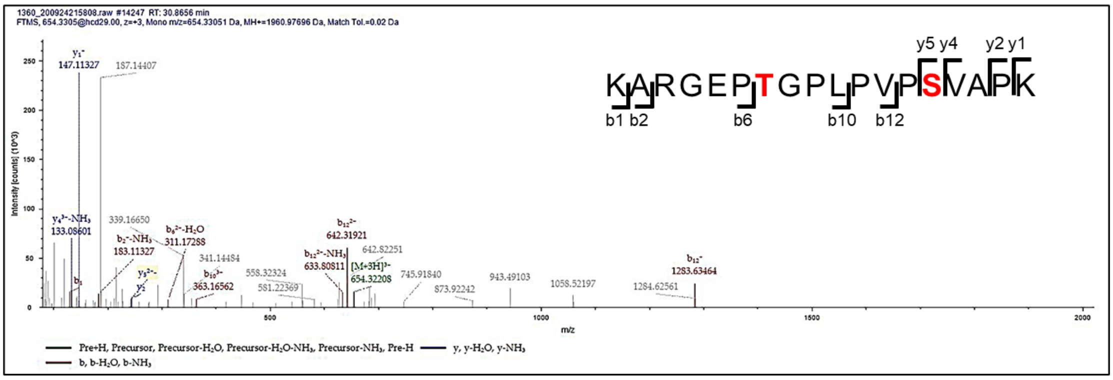
1360_200924215808.raw #14247 RT: 30.8656 min
FTMS, 654.3305@hcd29.00, z=+3, Mono m/z=654.33051 Da, MH+=1960.97696 Da, Match Tol.=0.02 Da
y5 y4 y2 y1
KARGEPTGPLPVPSVAPK
b1 b2 b6 b10 b12
m/z
147.11327
187.14407
642.31921
1283.63464
Pre+H, Precursor, Precursor-H2O, Precursor-H2O-NH3, Precursor-NH3, Pre-H
y, y-H2O, y-NH3
b, b-H2O, b-NH3

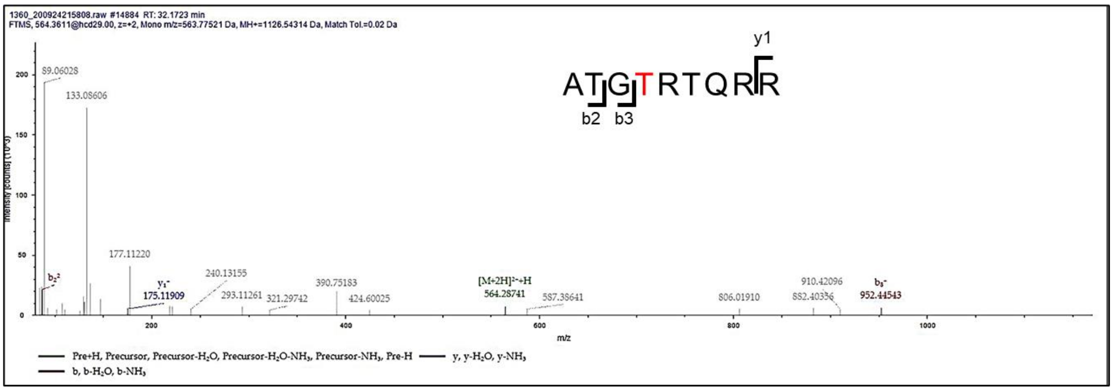
1360_200924215808.raw #14884 RT: 32.1723 min
FTMS, 564.3611@hcd29.00, z=+2, Mono m/z=563.77521 Da, MH+=1126.54314 Da, Match Tol.=0.02 Da
y1
ATGTRTQRR
b2 b3
m/z
89.06028
133.08606
177.11220
175.11909
952.44543
Pre+H, Precursor, Precursor-H2O, Precursor-H2O-NH3, Precursor-NH3, Pre-H
y, y-H2O, y-NH3
b, b-H2O, b-NH3

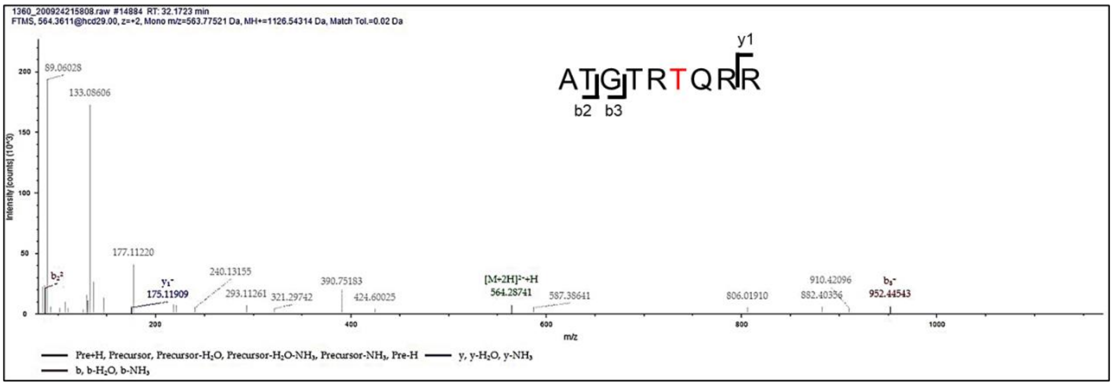
1360_200924215808.raw #14884 RT: 32.1723 min
FTMS, 564.3611@hcd29.00, z=+2, Mono m/z=563.77521 Da, MH+=1126.54314 Da, Match Tol.=0.02 Da
ATGTRTQRR
y1
b2 b3
Intensity [counts] (10^3)
m/z
Pre+H, Precursor, Precursor-H2O, Precursor-H2O-NH3, Precursor-NH3, Pre-H
y, y-H2O, y-NH3
b, b-H2O, b-NH3

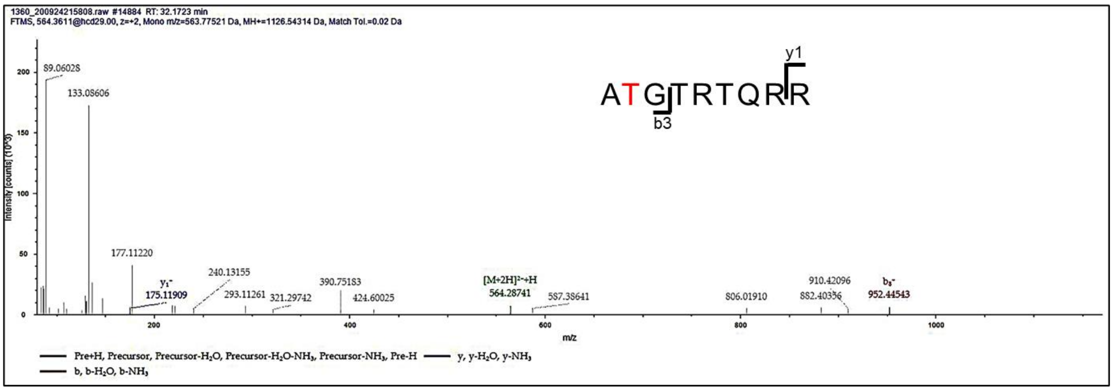
1360_200924215808.raw #14884 RT: 32.1723 min
FTMS, 564.3611@hcd29.00, z=+2, Mono m/z=563.77521 Da, MH+=1126.54314 Da, Match Tol.=0.02 Da
ATGTRTQRR
y1
b3
Intensity [counts] (10^3)
m/z
Pre+H, Precursor, Precursor-H2O, Precursor-H2O-NH3, Precursor-NH3, Pre-H
y, y-H2O, y-NH3
b, b-H2O, b-NH3

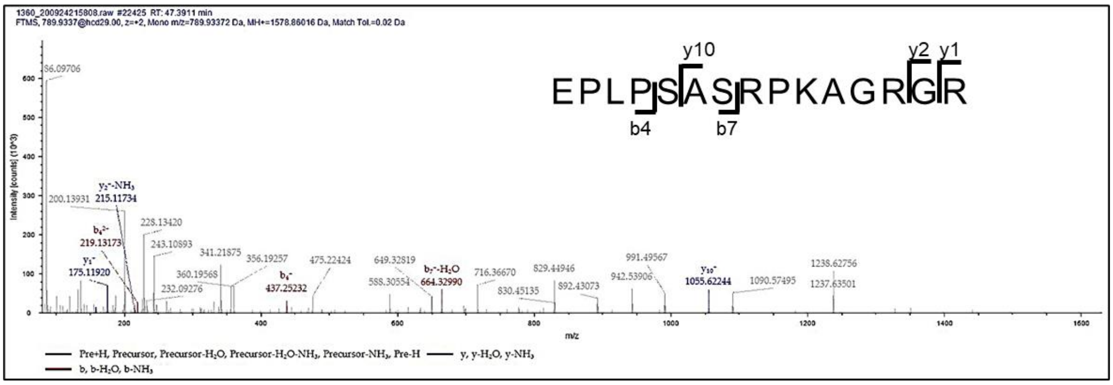
1360_200924215808.raw #22425 RT: 47.3911 min
FTMS, 789.9337@hcd29.00, z=+2, Mono m/z=789.93372 Da, MH+=1578.86016 Da, Match Tol.=0.02 Da
EPLPSASRPKAGRGR
y10
y2 y1
b4
b7
Intensity [counts] (10^3)
m/z
Pre+H, Precursor, Precursor-H2O, Precursor-H2O-NH3, Precursor-NH3, Pre-H
y, y-H2O, y-NH3
b, b-H2O, b-NH3

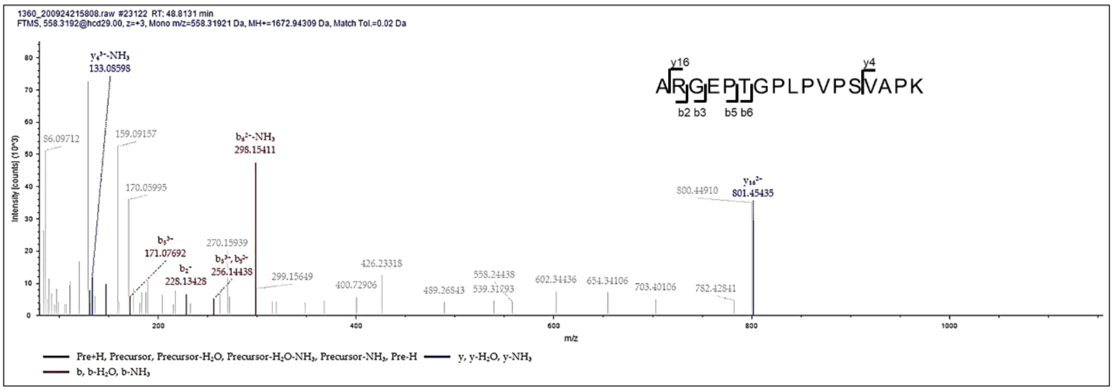
1360_200924215808.raw #23122 RT: 48.8131 min
FTMS, 558.3192@hcd29.00, z=+3, Mono m/z=558.31921 Da, MH+=1672.94309 Da, Match Tol.=0.02 Da
ARGEPTGPLPVPSVAPK
y16
y4
b2 b3
b5 b6
Intensity [counts] (10^3)
m/z
Pre+H, Precursor, Precursor-H2O, Precursor-H2O-NH3, Precursor-NH3, Pre-H
y, y-H2O, y-NH3
b, b-H2O, b-NH3

Supplementary Fig. S20. LC-MS/MS assay of PoxRsrA$_{901\text{-}1360}$ phosphorylation in Δ*POX_g08550*::*PoxRsrA*$_{901\text{-}1360}$*-his* and Δ*PoxKu70*::*PoxRsrA*$_{901\text{-}1360}$*-his*.

Phosphorylated amino acid residues are shown in red for Δ*POX_g08550*::*PoxRsrA*$_{901\text{-}1360}$-*his* and green for Δ*PoxKu70*::*PoxRsrA*$_{901\text{-}1360}$-*his*.

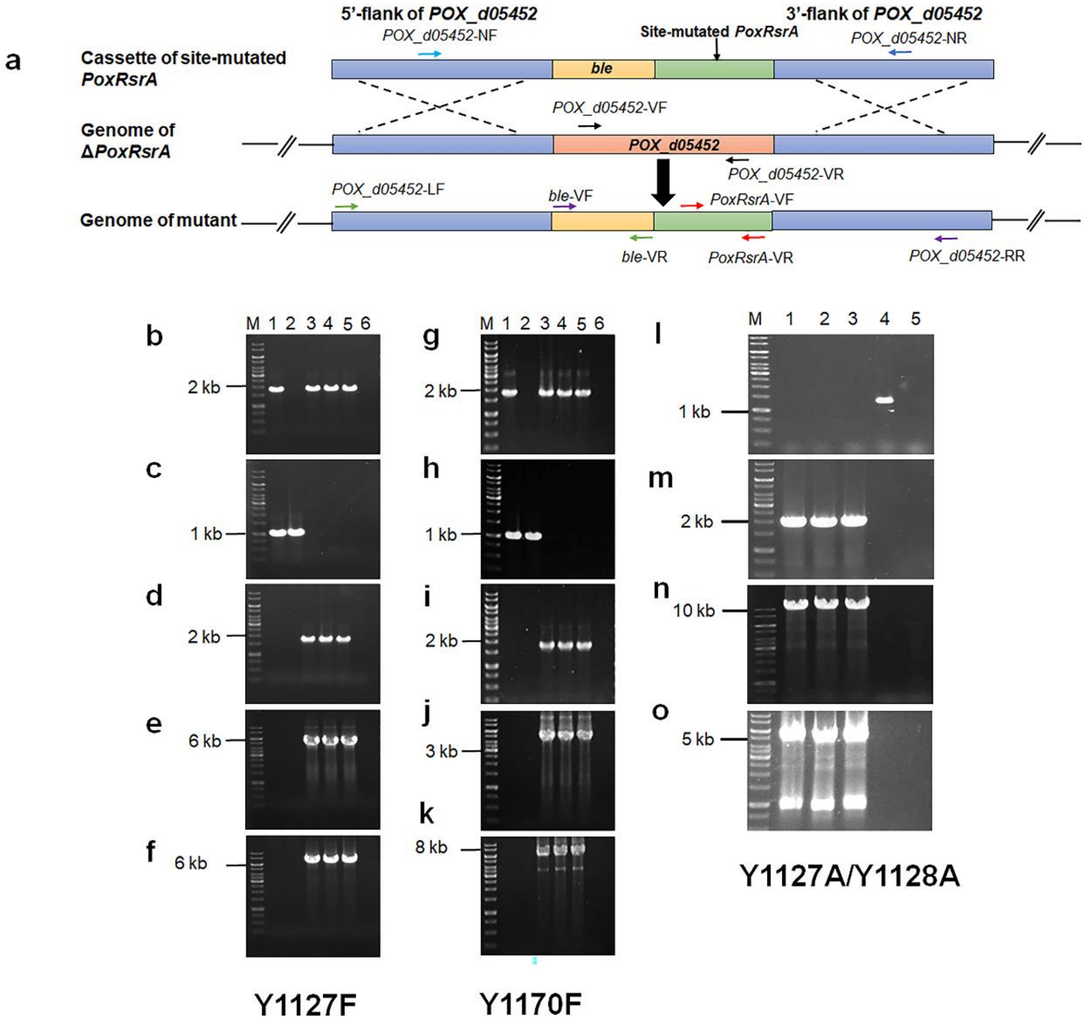

Supplementary Fig. S21. Construction of *P. oxalicum* mutants Y1127F, Y1170F and Y1127A/Y1128A.

a Construction strategy of *P. oxalicum* mutants. **b-f** PCR confirmation of the mutant Y1127F. **g–k** PCR confirmation of the mutant Y1170F. **l-o** PCR confirmation of the mutant Y1127A/Y1128A. **b** PCR production of *PoxRsrA* (using primers *PoxRsrA*-VF and *PoxRsrA*-VR). **c** PCR production of *POX_d05452* (using primers *POX_d05452*-VF and *POX_d05452*-VR). **d** PCR production of *ble* (using primers *ble*-VF and *ble*-VR). **e** PCR production of the

left-cross fragment (using primers *POX_d05452*-LF and *ble*-VR). **f** PCR production of the right-cross fragment (using primers *PoxRsrA*-VF and *POX_d05452*-RR). M: 1 kb Marker, 1: WT, 2: Δ*PoxRsrA*, 3–5: three different transformants of Y1127F, 6: ddH_2O. **g** PCR production of *PoxRsrA* (using primers *PoxRsrA*-VF and *PoxRsrA*-VR). **h** PCR production of *POX_d05452* (using primers *POX_d05452*-VF and *POX_d05452*-VR). **i** PCR production of *ble* (using primers *ble*-VF and *ble*-VR). **j** PCR production of left-cross fragment (using primers *POX_d05452*-LF and *ble*-VR). **k** PCR production of right-cross fragment (using primers *PoxRsrA*-VF and *POX_d05452*-RR). M: 1 kb DNA Marker, 1: WT, 2: Δ*PoxRsrA*, 3–5: three different transformants of Y1170F, 6: ddH_2O. **l** PCR production of *POX_d05452* (using primers *POX_d05452*-VF and *POX_d05452*-VR). **m** PCR production of left-cross fragment (using primers *POX_d05452*-LF and *ble*-VR). **n** PCR production of right-cross fragment (using primers *ble*-VF and *POX_d05452*-RR). **o** PCR production of *PoxRsrA* (using primers *PoxRsrA*-VF and *PoxRsrA*-VR). M: 1 kb DNA Marker, 1–3: three different transformants of Y1127A/Y1128A, 4: WT, 5: ddH_2O. WT: the wild-type strain. *ble*: bleomycin resistance gene.

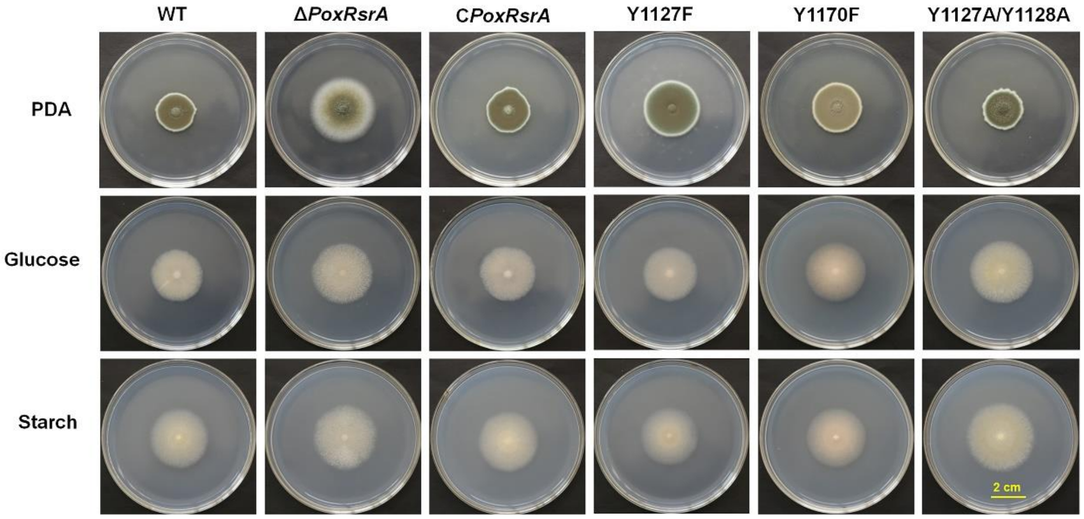

Supplementary Fig. S22. Colony investigation of *P. oxalicum* mutants Y1127F, Y1170F, and Y1127A/Y1128A on different carbon sources: potato dextrose agar (PDA), glucose, or soluble corn starch.

All tested strains are cultured on solid plates for 5 days. The wild-type strain (WT), mutant Δ*PoxRsrA* and complementation strain C*PoxRsrA* are used as controls.

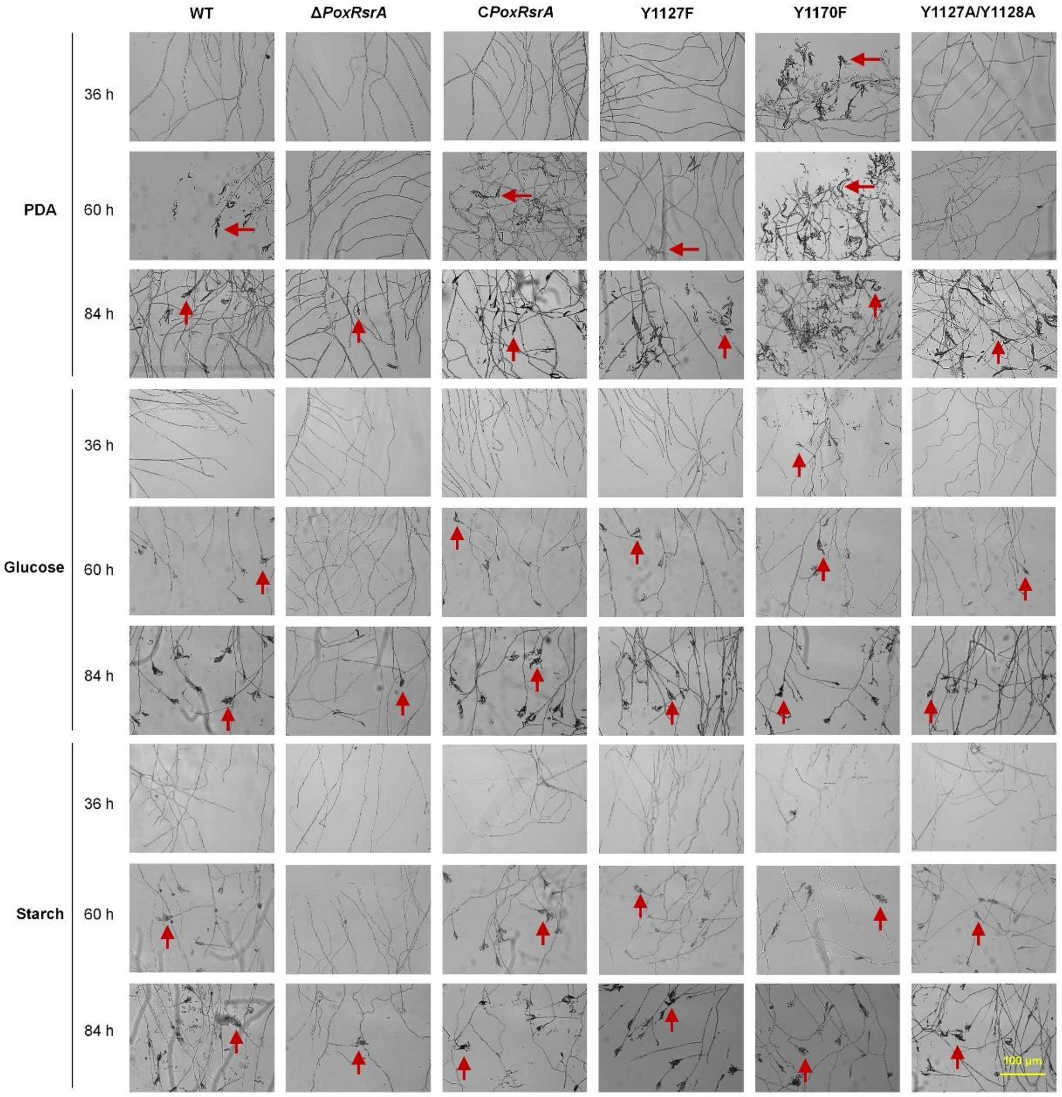

Supplementary Fig. S23. Mycelial investigation of *P. oxalicum* mutants Y1127F, Y1170F, and Y1127A/Y1128A on different carbon sources: potato dextrose agar (PDA), glucose, or soluble corn starch.

All tested strains are cultured on solid plates for 36, 60 and 84 h, respectively. The wild-type strain (WT), mutant Δ*PoxRsrA* and complementation strain C*PoxRsrA* are used as controls. The red arrows indicate conidiospores.

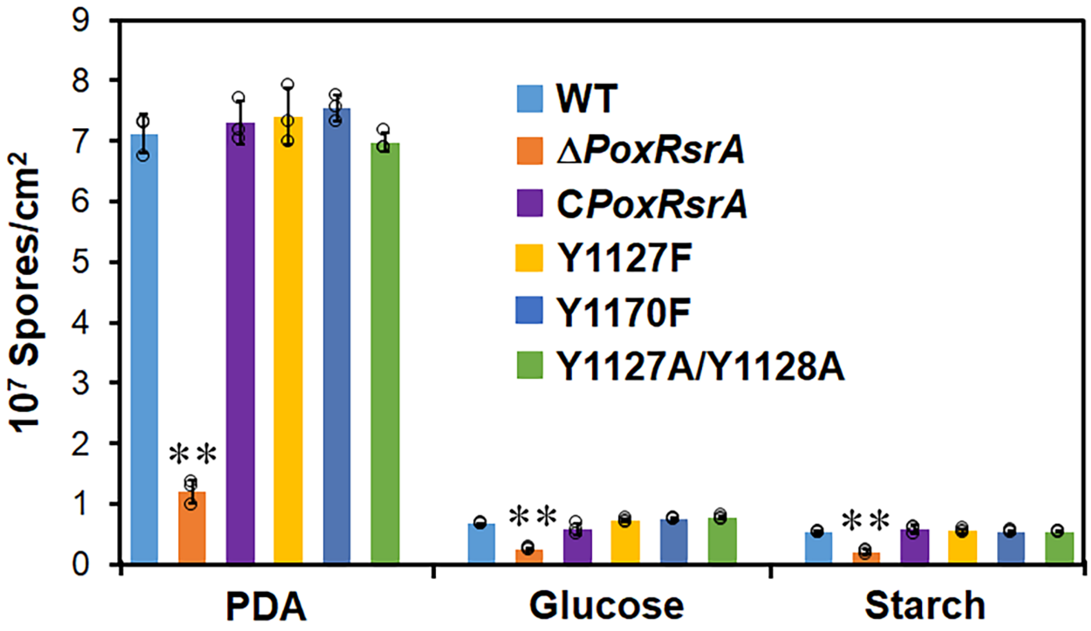

Supplementary Fig. S24. The production of asexual spores by *P. oxalicum* mutants Y1127F, Y1170F and Y1127A/Y1128A on different carbon sources: potato dextrose agar (PDA), glucose, or soluble corn starch.

All tested strains are cultured on solid plates for 5 days. The wild-type strain (WT), mutant Δ*PoxRsrA* and complementation strain C*PoxRsrA* are used as controls. **$p < 0.01$ indicates significant differences between mutants and WT or complementation strain, assessed by Student's *t*-test.

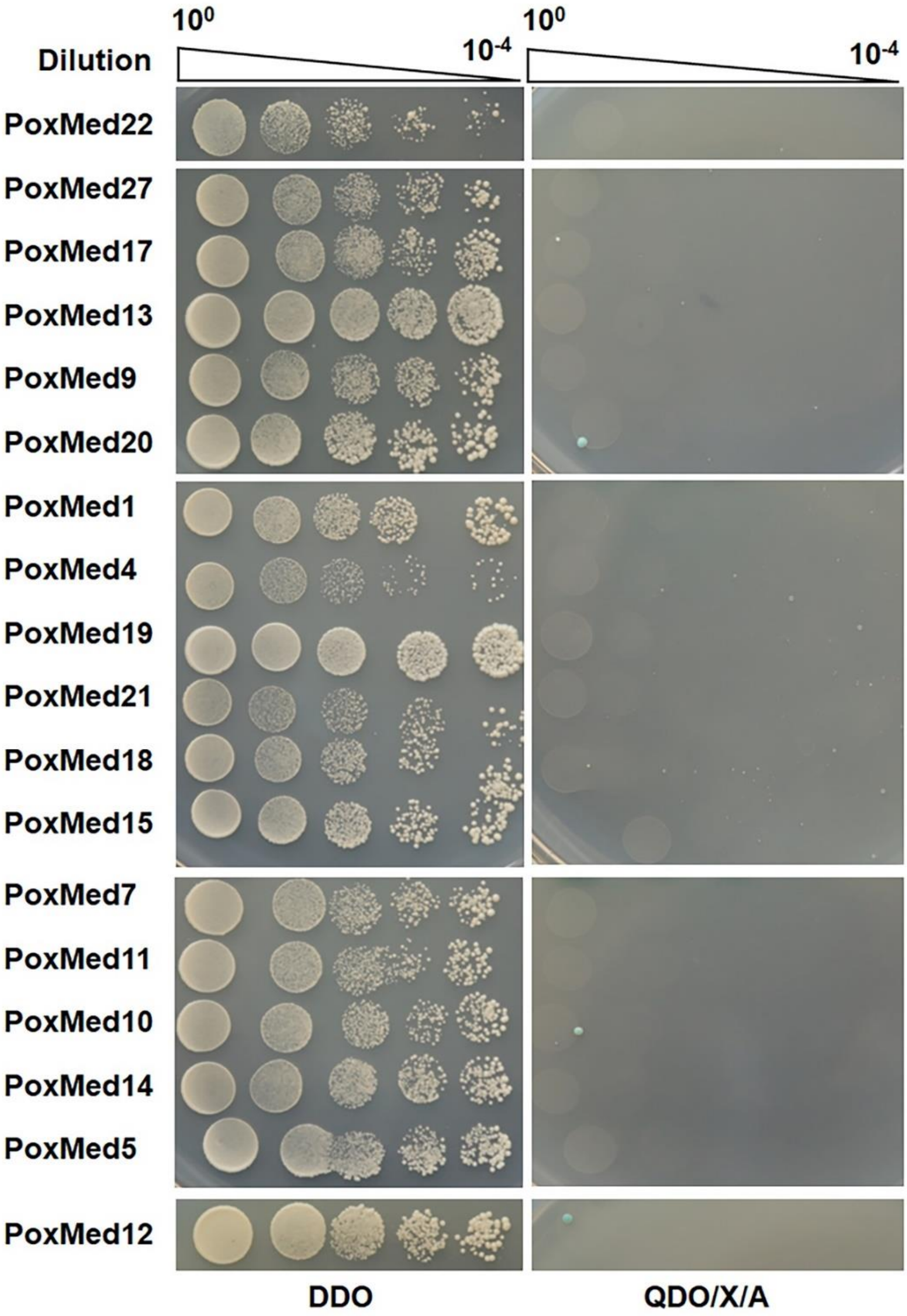

Supplementary Fig. S25. Yeast two-hybrid assay of PoxRsrA interacting with 18 Mediator subunits.

Yeast cells carrying PoxRsrA$_{1128–1730}$ as the bait and Mediator subunits as the preys are diluted by a tenfold gradient and then cultured on SD-Trp/-Leu (DDO), or SD-Trp/-Leu/-Ade/-His/+X-α-Gal/+AbA (QDO/X/A) for 4 days. The original concentration of each yeast sample was adjusted to OD600 = 1.0.

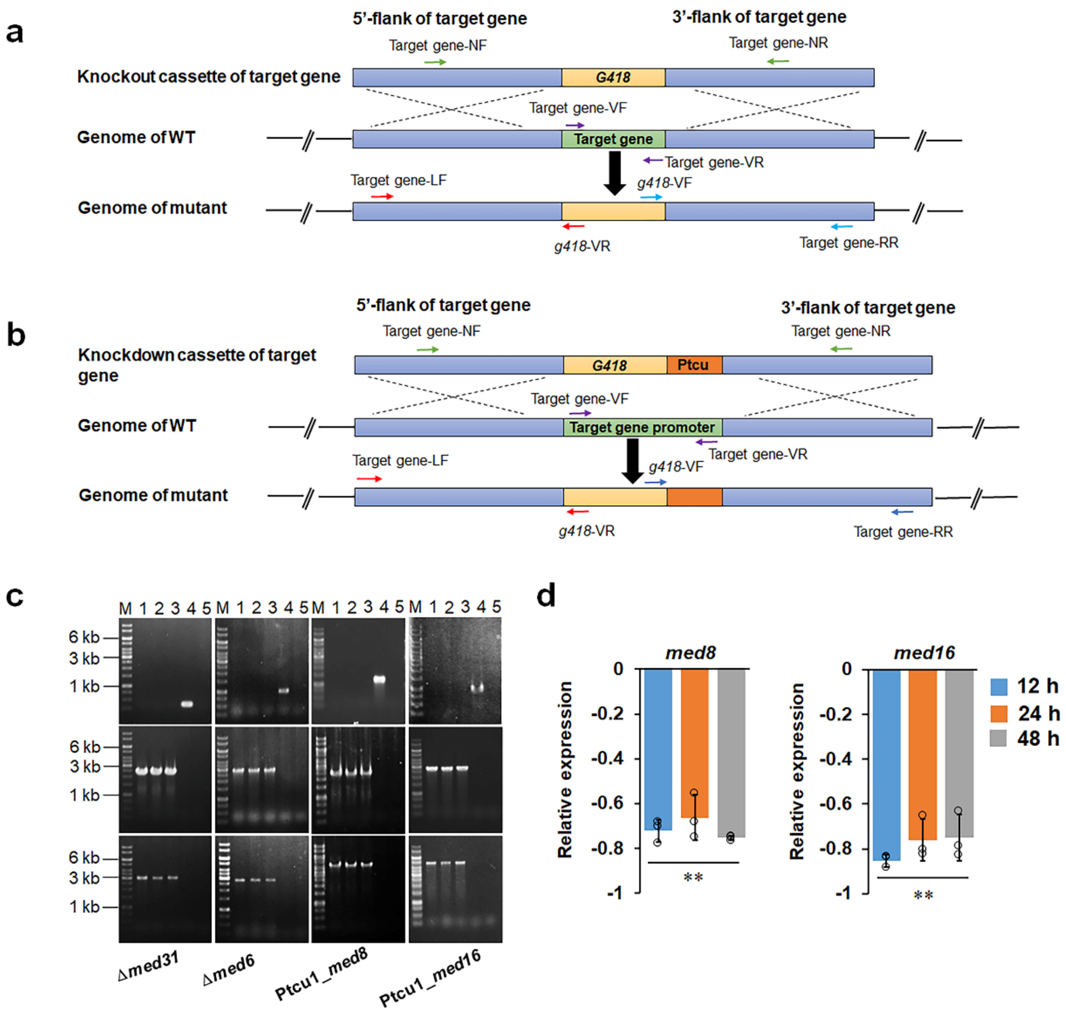

Supplementary Fig. S26. Construction of *P. oxalicum* mutants Δ*med31*, Δ*med6*, Ptcu-*med8* and Ptcu-*med16* and transcription levels of *med8* and *med16* in Ptcu-*med8* and Ptcu-*med16*.

a Construction strategy of the mutants Δ*med31*, Δ*med6*. **b** Construction strategy of the mutants Ptcu-*med8* and Ptcu-*med16*. **c** PCR confirmation of the mutants. Ptcu refers to the promoter of the copper transporter-encoding gene *tcu1*. *G418*: Geneticin resistance gene. In (**c**), M: 1 kb DNA Marker, 1: Mutant-1, 2: Mutant-2, 3: Mutant-3, 4: WT, 5: ddH_2O. The panels from top to bottom indicate PCR production of the target gene (using primers target gene-VF and

target gene-VR), left-cross fragment (using primers target gene-LF and *g418*-VR) and right-cross fragment (using primers *g418*-VF and target gene-RR). **d** Transcription levels of *med8* and *med16* in mutants Ptcu-*med8* and Ptcu-*med16*, respectively. Fungal strains are cultured in glucose medium for 24 h, then transferred into medium containing soluble corn starch with 5 μM Cu^{2+} and cultivated for 12–48 h. Expression levels of the tested genes in the mutants are normalized against those in the wild-type strain (WT). $^{**}p \leq 0.01$ indicated significant difference between mutant and WT. Each experiment was repeated three times.

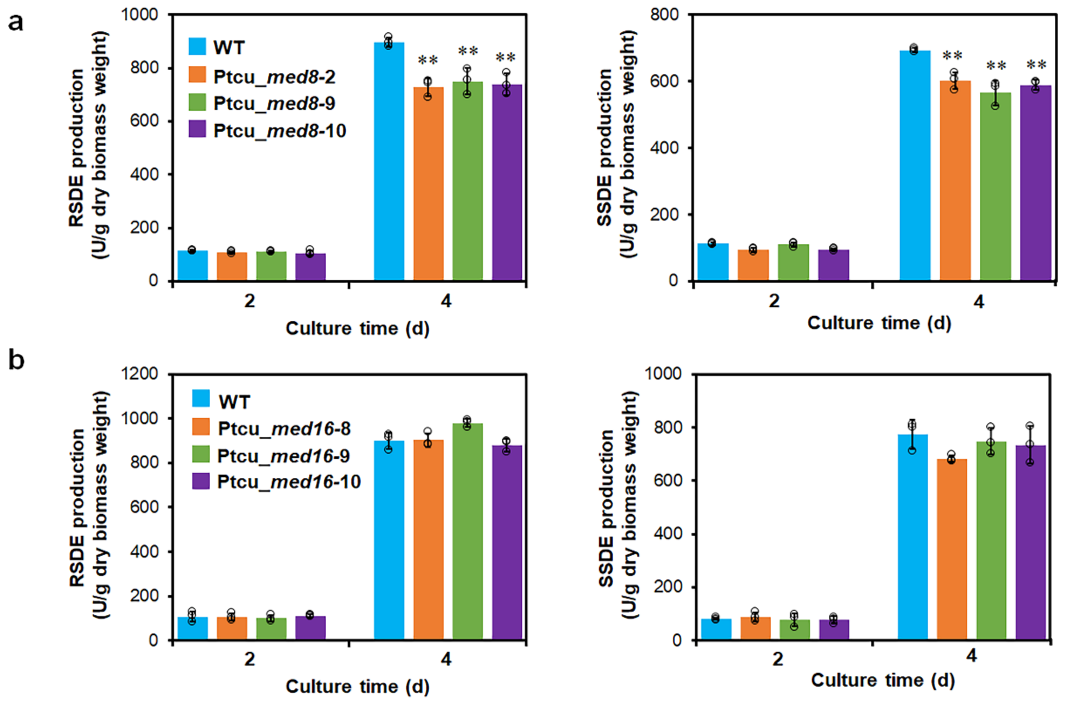

Supplementary Fig. S27. Amylase production by *P. oxalicum* mutants Ptcu-*med8* and Ptcu-*med16*.

a RSDE and SSDE production by the mutant Ptcu-*med8* and WT. **b** RSDE and SSDE production by the mutant Ptcu-*med31* and WT. All fungal strains are grown in medium containing soluble corn starch with 5 μM Cu^{2+} for 2–4 days after transfer from glucose. Data values are mean ± standard deviation. Each mutant includes three independent transformants. **p < 0.01 indicates significant differences between mutants and WT, assessed by Student's *t*-test. RSDE: raw starch degrading enzyme; SSDE: soluble starch degrading enzyme.

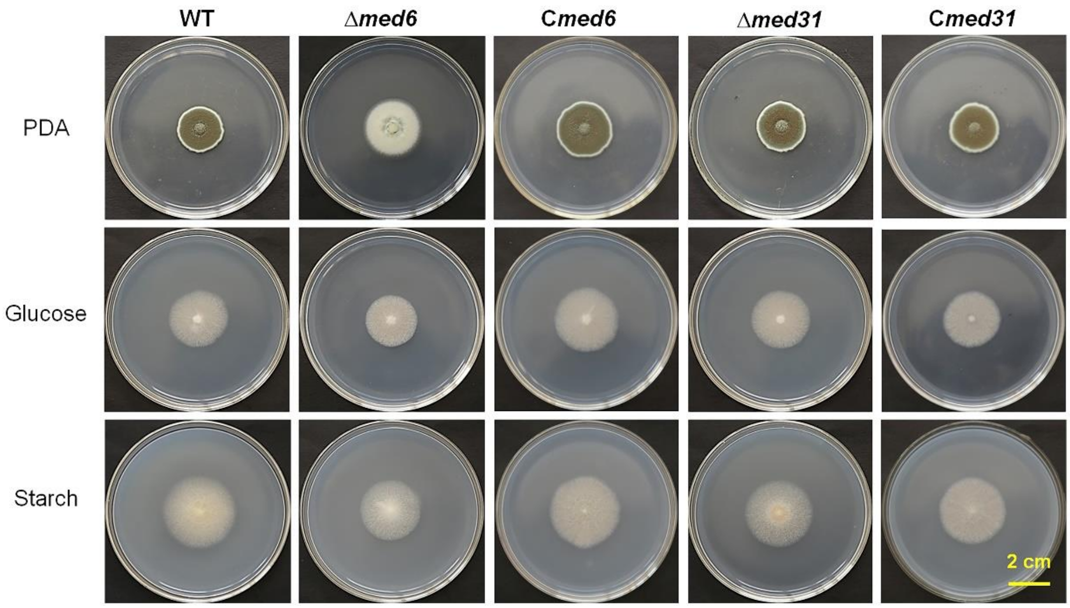

Supplementary Fig. S28. Colony investigation of *P. oxalicum* mutants ∆*med6* and ∆*med31*, and complementation strains C*med6* and C*med31* on different carbon sources including potato dextrose agar (PDA), glucose and soluble corn starch.

The wild-type strain (WT) was used as control. All strains are grown on solid plates for 5 days.

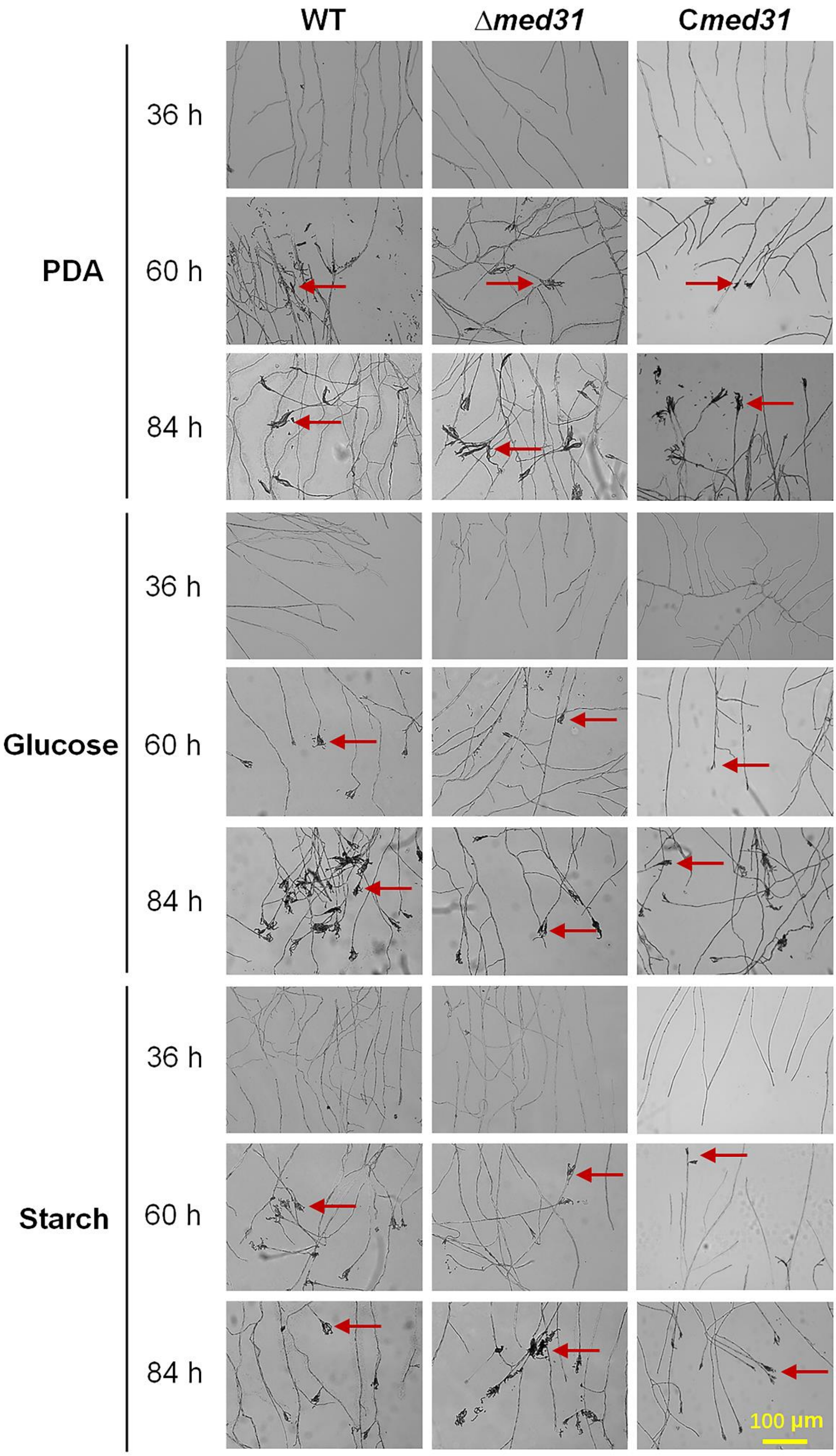
WT
Δmed31
Cmed31
PDA
Glucose
Starch
36 h
60 h
84 h
100 μm

Supplementary Fig. S29. Mycelial observation of *P. oxalicum* mutant Δ*med31* and complementation strain C*med31* in comparison with the wild-type strain (WT).

All strains were grown on solid plates containing potato dextrose agar (PDA), glucose, or soluble corn starch for 36 h, 60 h and 84 h. The red arrows indicate conidiospores.

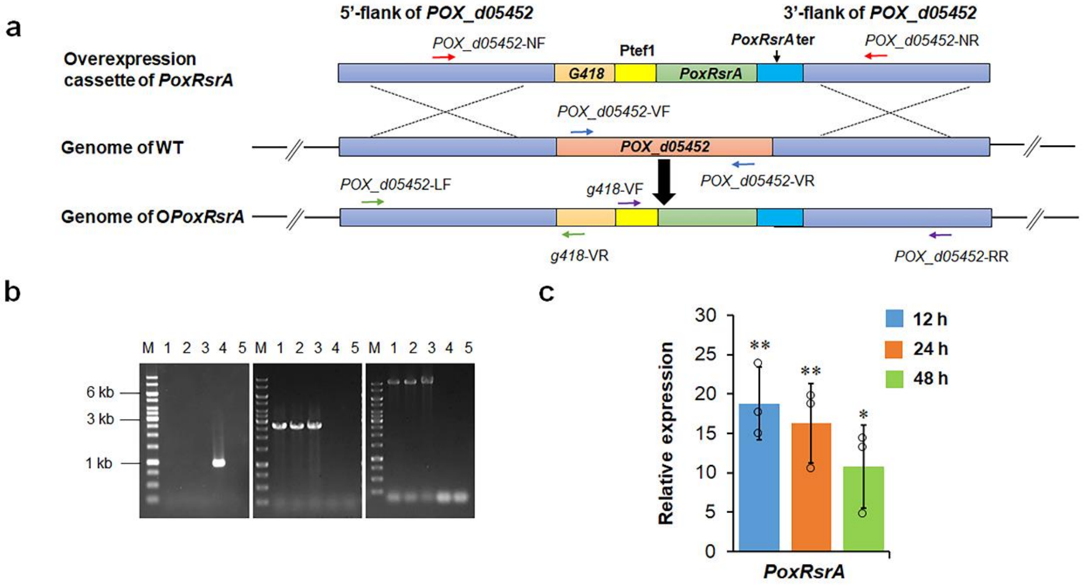

Supplementary Fig. S30. Construction of the *P. oxalicum* overexpression strain O*PoxRsrA* and transcription level of *PoxRsrA* in the strain O*PoxRsrA*.

a Construction strategy of the strain O*PoxRsrA*. **b** PCR verification of the strain O*PoxRsrA*. In (**a**), *PoxRsrA* ter refers to *PoxRsrA* terminator. *G418*: Geneticin resistance gene. WT: the wild-type strain. In (**b**), M: 1 kb DNA Marker; 1–3: three different transformants of O*PoxRsrA*; 4: Δ*PoxKu70*; 5: ddH$_2$O. The panels from left to right are PCR production of *POX_d05452* (using primers *POX_d05452*-VF and *POX_d05452*-VR), left-cross fragment (using primers *POX_d05452*-LF and *g418*-VR) and right-cross fragment (using primers *g418*-VF and *POX_d05452*-RR). **c** Transcription level of *PoxRsrA* in overexpression strain O*PoxRsrA*. Fungal strains are cultured in glucose medium for 24 h, then transferred into medium containing soluble corn starch and cultivated for 12–48 h. Expression levels of the tested genes in the mutants are normalized against those in the wild-type strain (WT). **$p \leq 0.01$ and *$p \leq 0.05$ indicated

significant difference between mutant and WT. Each experiment was repeated three times.

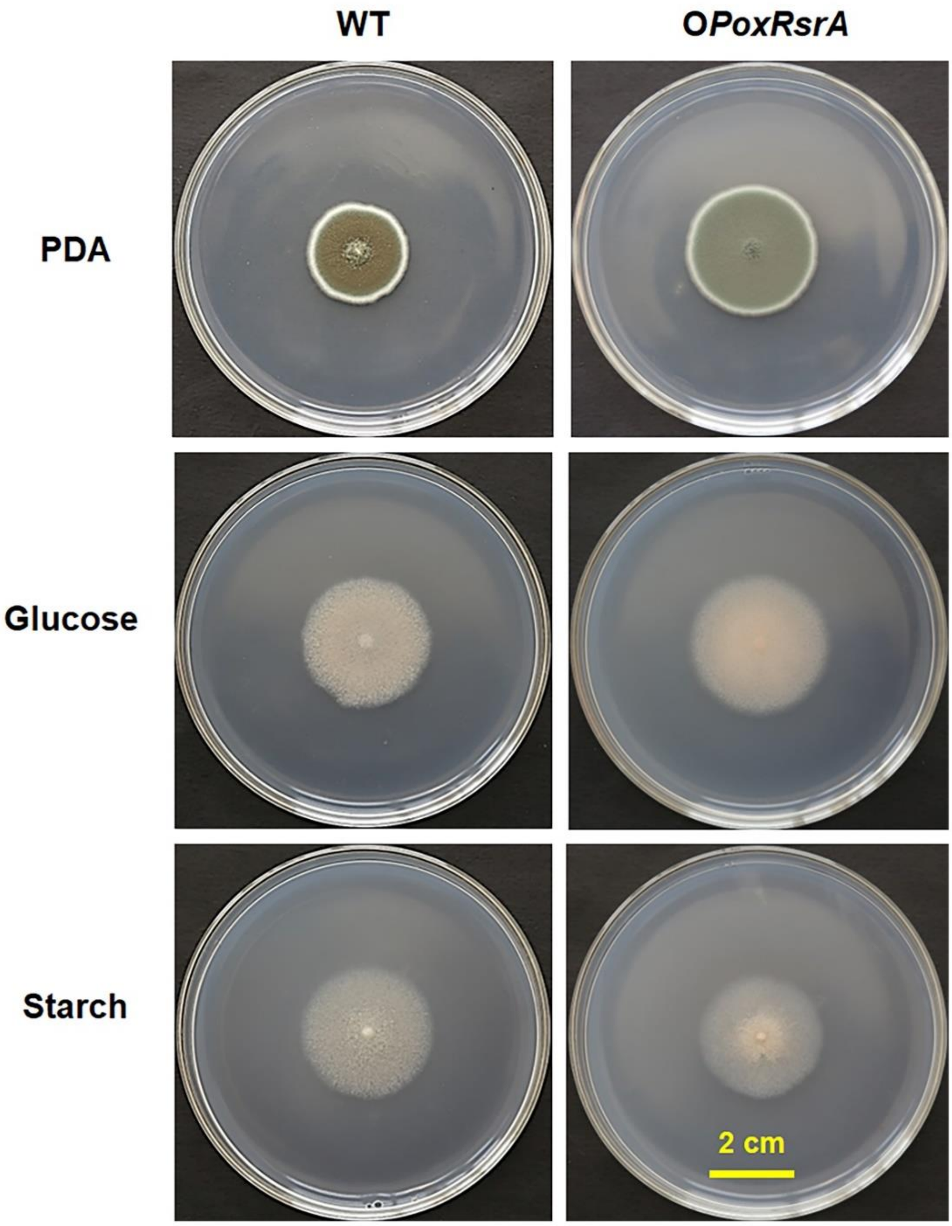

Supplementary Fig. S31. Colony observation of *P. oxalicum* overexpression strain O*PoxRsrA* and the wild-type strain (WT) on different carbon sources: potato dextrose agar (PDA), glucose or soluble corn starch.

All strains are grown on solid plates for 5 days.

Supplementary Table S1 *In vitro* TAP-MS detecting 12 candidates that interacted with oligopeptide PoxRsrA$_{901–1360}$

Protein ID	Functional prediction	Sequence identified by LC-MS
POX_e06400	Heat shock protein 70 family	[K].LVSDFFNK.[D]/[R].IINEPTAAAIAYGLDK.[K]/[R].FEELCQDLFR.[S]
POX_g08550	3-hydroxyisobutyryl-CoA hydrolase	[R].VGFVSAVYENR.[A]/[R].IFGADEALR.[V]/[R].QVFNQLSEDSSVR.[A]/[K].AASQGGALAGEASVDPAR.[K]/[R].FPDESPYVAHVEINR.[A]/[R].VGFVSAVYENR.[A]/[R].AIVLSGAGEK.[A]/[R].IFGADEALR.[V]
POX_a01268	Cytochrome c-like domain	[R].NDLITYLK.[E]/[K].VGPNLHGLFGR.[K]/[K].DAGFTPGDAEK.[G]/[K].QADVTWDEK.[T]/[K].MAFGGLK.[K]
POX_a01477	Pleckstrin homology domain	[R].TSFFGNFGK.[K]/[R].TSFFGNFGK.[K]/[R].SSIFGSLLGK.[K]/[K].LGGLFR.[K]/[K].LDTEEVPAAAAEAETKPVEAK.[E]
POX_b03033	Ribosomal protein S27a	[R].TLSDYNIQK.[EV]/[-G].MQIFVK.[T]/[K].ESTLHLVLR.[L]
POX_e06638	Translation elongation factor EFTu/EF1A	[K].TLLEAIDAIEPPVRPSNKPLR.[L]/[K].FSELLEK.[I]/[K].TLLEAIDAIEPPVRPSNKPLR.[L]
POX_a00047	Glyceraldehyde 3-phosphate dehydrogenase, NAD(P) binding domain	[K].AGIALNSNFIK.[L]/[K].LVSWYDNEWGYSHR.[V]/[K].VINDNFGLVEGLMTTVHSYTATQK.[T]/[R].TAAQNIIPSSTGAAK.[A]
POX_a01111	6-phosphogluconate dehydrogenase, NADP-binding	[K].DLPANLLQAQR.[D]
POX_b03158	Aspartic peptidase domain	[K].HQDFAEATQEPGLAFAFGR.[F]/[R].YGSGSLSGFVSQDTLK.[I]/[R].FDGILGLGFDTISVNK.[M]
POX_a00855	Citrate synthase active site	[R].ALGVLPQLIIDR.[A]
POX_a01318	Translation elongation factor EFTu/EF1A, domain 2	[K].DSVVSGFQWATR.[E]
POX_b02345	Basic-leucine zipper domain	[K].LDGALFEAVEHDKLPSKSVPPLSCDTSHEPGLSDVMFDNLVNILE.[-]

Supplementary Table S2 *Penicillium oxalicum* strains used in this study

Strain	Description	Genotypes	References
WT (Δ*PoxKu70*)	Deletion strain of *PoxKu70*	*PoxKu70*$^{-}$; *Hph*$^{R+}$	1
Δ*PoxRsrA*	Deletion strain of *PoxRsrA*	*PoxKu70*$^{-}$; *PoxRsrA*$^{-}$; *Hph*$^{R+}$; G418^{R+}	2
C*PoxRsrA*	Complementation strain of Δ*PoxRsrA*	*PoxKu70*$^{-}$; *Hph*$^{R+}$; G418^{R+}; *Ble*$^{R+}$	2
Δ1440-1794	Deletion strain of *PoxRsrA*$_{1440\text{-}1794}$	*PoxKu70*$^{-}$; *PoxRsrA*$_{1440\text{-}1794}^{-}$; *Hph*$^{R+}$; *G418*$^{R+}$	This study
Δ1135-1794	Deletion strain of *PoxRsrA*$_{1135\text{-}1794}$	*PoxKu70*$^{-}$; *PoxRsrA*$_{1135\text{-}1794}^{-}$; *Hph*$^{R+}$; *G418*$^{R+}$	This study
Δ1080-1794	Deletion strain of *PoxRsrA*$_{1080\text{-}1794}$	*PoxKu70*$^{-}$; *PoxRsrA*$_{1080\text{-}1794}^{-}$; *Hph*$^{R+}$; *G418*$^{R+}$	This study
Δ891-1794	Deletion strain of *PoxRsrA*$_{891\text{-}1794}$	*PoxKu70*$^{-}$; *PoxRsrA*$_{891\text{-}1794}^{-}$; *Hph*$^{R+}$; *G418*$^{R+}$	This study
Δ827-1794	Deletion strain of *PoxRsrA*$_{827\text{-}1794}$	*PoxKu70*$^{-}$; *PoxRsrA*$_{827\text{-}1794}^{-}$; *Hph*$^{R+}$; *G418*$^{R+}$	This study
Δ1-1084	Deletion strain of *PoxRsrA*$_{1\text{-}1084}$	*PoxKu70*$^{-}$; *PoxRsrA*$_{1\text{-}1084}^{-}$; *Hph*$^{R+}$; *G418*$^{R+}$	This study
Δ1-881	Deletion strain of *PoxRsrA*$_{1\text{-}881}$	*PoxKu70*$^{-}$; *PoxRsrA*$_{1\text{-}881}^{-}$; *Hph*$^{R+}$; *G418*$^{R+}$	This study
Δ1-831	Deletion strain of *PoxRsrA*$_{1\text{-}831}$	*PoxKu70*$^{-}$; *PoxRsrA*$_{1\text{-}831}^{-}$; *Hph*$^{R+}$; *G418*$^{R+}$	This study
Δ1-703	Deletion strain of *PoxRsrA*$_{1\text{-}703}$	*PoxKu70*$^{-}$; *PoxRsrA*$_{1\text{-}703}^{-}$; *Hph*$^{R+}$; *G418*$^{R+}$	This study
Δ1-565	Deletion strain of *PoxRsrA*$_{1\text{-}565}$	*PoxKu70*$^{-}$; *PoxRsrA*$_{1\text{-}565}^{-}$; *Hph*$^{R+}$; *G418*$^{R+}$	This study
Δ1-467	Deletion strain of *PoxRsrA*$_{1\text{-}467}$	*PoxKu70*$^{-}$; *PoxRsrA*$_{1\text{-}467}^{-}$; *Hph*$^{R+}$; *G418*$^{R+}$	This study
Δ*SANT2*	Mutant Δ*PoxRsrA* carrying *PoxRsrA* lacking *SANT2*	*PoxKu70*$^{-}$; *PoxRsrA*$^{-}$; *PoxRsrA* lacking *SANT2*; *Hph*$^{R+}$; *G418*$^{R+}$; *Ble*$^{R+}$	This study
D1508A	Mutant Δ*PoxRsrA* carrying *PoxRsrA*^{D1508A}	*PoxKu70*$^{-}$; *PoxRsrA*$^{-}$; *PoxRsrA*$^{D1508A+}$; *Hph*$^{R+}$; *G418*$^{R+}$; *Ble*$^{R+}$	This study
W1509A	Mutant Δ*PoxRsrA* carrying *PoxRsrA*^{W1509A}	*PoxKu70*$^{-}$; *PoxRsrA*$^{-}$; *PoxRsrA*$^{W1509A+}$; *Hph*$^{R+}$; *G418*$^{R+}$; *Ble*$^{R+}$	This study
M1510A	Mutant Δ*PoxRsrA* carrying *PoxRsrA*^{M1510A}	*PoxKu70*$^{-}$; *PoxRsrA*$^{-}$; *PoxRsrA*$^{M1510A+}$; *Hph*$^{R+}$; *G418*$^{R+}$; *Ble*$^{R+}$	This study

R866K	Mutant Δ*PoxRsrA* carrying *PoxRsrA*^{R866K}	*PoxKu70*$^{–}$; *PoxRsrA*$^{–}$; *PoxRsrA*$^{R866K+}$; *Hph*$^{R+}$; *G418*$^{R+}$; *Ble*$^{R+}$	This study
R866A	Mutant Δ*PoxRsrA* carrying *PoxRsrA*^{R866A}	*PoxKu70*$^{–}$; *PoxRsrA*$^{–}$; *PoxRsrA*$^{R866A+}$; *Hph*$^{R+}$; *G418*$^{R+}$; *Ble*$^{R+}$	This study
Y1127F	Mutant Δ*PoxRsrA* carrying *PoxRsrA*^{Y1127F}	*PoxKu70*$^{–}$; *PoxRsrA*$^{–}$; *PoxRsrA*$^{Y1127F+}$; *Hph*$^{R+}$; *G418*$^{R+}$; *Ble*$^{R+}$	This study
Y1170F	Mutant Δ*PoxRsrA* carrying *PoxRsrA*^{Y1170F}	*PoxKu70*$^{–}$; *PoxRsrA*$^{–}$; *PoxRsrA*$^{Y1170F+}$; *Hph*$^{R+}$; *G418*$^{R+}$; *Ble*$^{R+}$	This study
Y1127A/1128A	Mutant Δ*PoxRsrA* carrying *PoxRsrA*$^{Y1127A/1128A}$	*PoxKu70*$^{–}$; *PoxRsrA*$^{–}$; *PoxRsrA*$^{Y1127A/Y1128A+}$; *Hph*$^{R+}$; *G418*$^{R+}$; *Ble*$^{R+}$	This study
PoxRsrA::GFP	In the WT, *PoxRsrA* was replaced by *PoxRsrA::gfp*	*PoxKu70*$^{–}$; *PoxRsrA::gfp*$^{+}$; *Hph*$^{R+}$; *G418*$^{R+}$	This study
Δ1731-1794::*gfp*	Deletion strain of *PoxRsrA*$_{1731-1794}$; heterologous expression of *gfp-his*	*PoxKu70*$^{–}$; *PoxRsrA*$_{1731-1794}$$^{–}$; *PoxRsrA*$_{1731-1794}$::*gfp-his*$^{+}$; *Hph*$^{R+}$; *G418*$^{R+}$.	This study
Ptef1-*PoxRsrA*	In the WT, the native promoter of *PoxRsrA* was replaced by the promoter Ptef1	*PoxKu70*$^{–}$; Ptef1-*PoxRsrA* $^{+}$; *Hph*$^{R+}$; *G418*$^{R+}$	This study
O*PoxRsrA*	Overexpression strain of gene *PoxRsrA*	*PoxKu70*$^{–}$; *PoxRsrA*$^{++}$; *Hph*$^{R+}$; *G418*$^{R+}$	This study
Δ*med31*	Deletion strain of *PoxMed31*	*PoxKu70*$^{–}$; *PoxMed31*$^{–}$; *Hph*$^{R+}$; *G418*$^{R+}$	This study
C*med31*	Complementation strain of Δ*med31*	*PoxKu70*$^{–}$; *Hph*$^{R+}$; *G418*$^{R+}$; *Ble*$^{R+}$	This study
Δ*med6*	Deletion strain of *PoxMed6*	*PoxKu70*$^{–}$; *PoxMed6*$^{–}$; *Hph*$^{R+}$; *G418*$^{R+}$	This study
C*med6*	Complementation strain of Δ*med6*	*PoxKu70*$^{–}$; *Hph*$^{R+}$; *G418*$^{R+}$; *Ble*$^{R+}$	This study
P_{tcu1}-*med8*	Knockdown strain of *PoxMed8*	*PoxKu70*$^{–}$; *PoxMed8*$^{–}$; Ptcu1-*med8*$^{+}$; *Hph*$^{R+}$; *G418*$^{R+}$	This study
P_{tcu1}-*med16*	Knockdown strain of *PoxMed16*	*PoxKu70*$^{–}$; *PoxMed16*$^{–}$; Ptcu1-*med16*$^{+}$; *Hph*$^{R+}$; *G418*$^{R+}$	This study
Δ*POX_g08550*	Deletion strain of *POX_g08550*	*PoxKu70*$^{–}$; *POX_g08550*$^{–}$; *Hph*$^{R+}$; *G418*$^{R+}$	This study
C*POX_g08550*	Complementation strain of *POX_g08550*	*PoxKu70*$^{–}$; *Hph*$^{R+}$; *G418*$^{R+}$; *Ble*$^{R+}$	This study
O*PoxRsrA*$_{901-1360}$	Overexpression strain of *PoxRsrA*$_{901-1360}$	*PoxKu70*$^{–}$; *Hph*$^{R+}$; *PoxRsrA*$_{901-1360}$$^{++}$; *G418*$^{R+}$	This study

O*POX_g08550*	Overexpression strain of *POX_g08550*	*PoxKu70*$^{-}$; *Hph*$^{R+}$; *POX_g08550*$^{++}$; *G418*$^{R+}$	This study
Δ*POX_g08550*:: *PoxRsrA*$_{901\text{-}1360}$	Mutant where *POX_g08550* is deleted and *POXRSRA*$_{901\text{-}1360}$ is overexpressed	*PoxKu70*$^{-}$; *Hph*$^{R+}$; *POX_g08550*$^{-}$; *PoxRsrA*$_{901\text{-}1360}$$^{++}$; *G418*$^{R+}$; *Ble*$^{R+}$	This study
O*POX_g08550*:: *PoxRsrA*$_{901\text{-}1360}$	Mutant where *POX_g08550* and *PoxRsrA*$_{901\text{-}1360}$ are overexpressed	*PoxKu70*$^{-}$; *Hph*$^{R+}$; *POX_g08550*$^{++}$; *PoxRsrA*$_{901\text{-}1360}$$^{++}$; *G418*$^{R+}$; *Ble*$^{R+}$	This study

References

1. Zhao, S., Yan, Y.S., He, Q.P., Yang, L., Yin, X., Li, C.X., Mao, L.C., Liao, L.S., Huang, J.Q., Xie, S.B., Nong, Q.D., Zhang, Z., Jing, L., Xiong, Y.R., Duan, C.J., Liu, J.L., & Feng, J.X. Comparative genomic, transcriptomic and secretomic profiling of *Penicillium oxalicum* HP7-1 and its cellulase and xylanase hyper-producing mutant EU2106, and identification of two novel regulatory genes of cellulase and xylanase gene expression. *Biotechnol Biofuels* **9**, 203 (2016).
2. Zhang, M. Y., Zhao, S., Ning, Y. N., Fu, L. H., Li, C. X., Wang, Q., You, R., Wang, C. Y., Xu, H. N., Luo, X. M., & Feng, J. X. Identification of an essential regulator controlling the production of raw-starch-digesting glucoamylase in *Penicillium oxalicum*. *Biotechnol. Biofuels* **12**, 7 (2019).
