# Supplementary material for: Regulation of fungal raw-starch-degrading enzyme production depends on transcription factor phosphorylation and recruitment of the Mediator complex
Source: Commun Biol. 2023 Oct 12;6:1032. doi: 10.1038/s42003-023-05404-x (PMC10570388; doi:10.1038/s42003-023-05404-x)
Supplement: Supplementary file 2 — Description of Additional Supplementary Data [file 42003_2023_5404_MOESM2_ESM.docx]

**Description of Additional Supplementary Files**

**File name:** Supplementary Data 1

**Description:** Primers used in this study

**File name:** Supplementary Data 2

**Description:** Source data presented in the main figures in Excel format.

**File name:** Supplementary Data 3

**Description:** Uncropped and unedited blots/gels presented in the main figures.
