# Supplementary material for: Regulation of fungal raw-starch-degrading enzyme production depends on transcription factor phosphorylation and recruitment of the Mediator complex
Source: Commun Biol. 2023 Oct 12;6:1032. doi: 10.1038/s42003-023-05404-x (PMC10570388; doi:10.1038/s42003-023-05404-x)
Supplement: Supplementary file 3 — Supplementary Data 1_4revised [file 42003_2023_5404_MOESM3_ESM.docx]

**Supplementary Data**

**Regulation of fungal raw-starch-degrading enzyme production depends on transcription factor phosphorylation and recruitment of the Mediator complex**

Yuan-Ni Ning^1,2,3^, Di Tian^1,2,3^, Man-Li Tan^3^, Xue-Mei Luo^3^, Shuai Zhao^1,2,3^*, Jia-Xun Feng^1,2,3^*

^1^State Key Laboratory for Conservation and Utilization of Subtropical Agro-bioresources, Guangxi University, 100 Daxue Road, Nanning, Guangxi 530004, People’s Republic of China.

^2^Guangxi Research Center for Microbial and Enzyme Engineering Technology, Guangxi University, 100 Daxue Road, Nanning, Guangxi 530004, People’s Republic of China.

^3^College of Life Science and Technology, Guangxi University, 100 Daxue Road, Nanning, Guangxi 530004, People’s Republic of China.

**Content:**

**Supplementary Data 1** Primers used in this study

*Correspondence: jiaxunfeng@sohu.com; shuaizhao0227@gxu.edu.cn

Tel: +86-771-323-9401

Mailing address: College of Life Science and Technology, Guangxi University, 100 Daxue Road, Nanning, Guangxi 530004, People’s Republic of China

**Supplementary Data 1** Primers used in this study

| **Name** | **Sequence (5’-3’)** |
| --- | --- |
| **Primers used for construction of mutants where the key amino acid residues required for transcriptional activation were site-mutated** | |
| *POX_d05452*-LF | GGCACCAAACACCGCTACA |
| *POX_d05452*-LR | TTTAGAGGTAATCCTTCTTTCTAGAGATGGACGAGACGGTACGATT |
| *POX_d05452*-RF | ATGTAGATCACCAAGTTGCA |
| *POX_d05452*-RR | TTCGGACGGTGTCACTTCG |
| *POX_d05452*-NF | AATGAGCGTAGTGTGAGAGCACCAAT |
| *POX_d05452*-NR | GTCGCACATCCAATCGCATAGAGT |
| *POX_d05452*-VF | CGCCTACGGAAAATGACG |
| *POX_d05452*-VR | ATGTCGCCGAAGATGGAG |
| *PoxRsrA*-VF | GTTCCTGCCTTCGGTTCA |
| *PoxRsrA*-VR | TCGCATCACTCGGGTCAA |
| *PoxRsrA*p-F | TCTTACCGCTGTTGAGATCCAGAGGGCTCAGGTTCAATGGGT |
| *PoxRsrA*ter-R | CGTGCAACTTGGTGATCTACATACCGTGCTGGTTTTCTCTGTG |
| *PoxRsrA*^D1508A^-F | ACAATGCATGGATGGCTCGCTTCGATCCGAGAGCTCAACA |
| *PoxRsrA*^D1508A^-R | GCGAGCCATCCATGCATTGTTGACAGCATTTCCCGGGCCA |
| *PoxRsrA*^W1509A^-F | ACAATGATGCAATGGCTCGCTTCGATCCGAGAGCTC |
| *PoxRsrA*^W1509A^-R | GCGAGCCATTGCATCATTGTTGACAGCATTTCCCG |
| *PoxRsrA*^M1510A^-F | CTGTCAACAATGATTGGGCAGCTCGCTTCGATCCGAGAGC |
| *PoxRsrA*^M1510A^-R | GCTCTCGGATCGAAGCGAGCTGCCCAATCATTGTTGACAG |
| *ble*-F | TCTAGAAAGAAGGATTACCTC |
| *ble*-R | CTGGATCTCAACAGCGGTA |
| **Primers used for construction of mutants where different regions of PoxRsrA was deleted** | |
| *PoxRsrA*_1440-1794_-LF | ACAATCTATCGGACCTGTGCT |
| *PoxRsrA*_1440-1794_-LR | CGCGGAGATTAGATGTAGCTATATTTAGTTGGATGAAACTTCACTAGCA |
| *PoxRsrA*_1440-1794_-NF | CGAGTGATGCGATGGAAGTGT |
| *PoxRsrA*_1440-1794_-NR | GCGTCTGCGTCATTCACATT |
| *PoxRsrA*_1440-1794_-VF | GGTCAACCAAGCCCTTATCA |
| *PoxRsrA*_1440-1794_-VR | AAGGCTGAAGGGGGAGTGTA |
| *PoxRsrA*_1135-1794_-LF | GGTTCCTGCCTTCGGTTCA |
| *PoxRsrA*_1135-1794_-LR | CGCGGAGATTAGATGTAGCTATATTTAAGAGTCAAGGCGTCGCTGGTA |
| *PoxRsrA*_1135-1794_-NF | TCCCGCTCAGCGTACAACC |
| *PoxRsrA*_1135-1794_-NR | TCGTGGACCGTTTACGGCT |
| *PoxRsrA*_1135-1794_-VF | GTCTGGACCTCGTATTGGATT |
| *PoxRsrA*_1135-1794_-VR | TTGCCGCTGTGAACTGG |
| *PoxRsrA*_1080-1794_-LF | GTTCCTGCCTTCGGTTCA |
| *PoxRsrA*_1080-1794_-LR | CGCGGAGATTAGATGTAGCTATATTTA ACTTTCAGAAGGCTTGGGTGT |
| *PoxRsrA*_1080-1794_-NF | CGTACAACCCAAGAATCTGT |
| *PoxRsrA*_1080-1794_-NR | CCAGTAGGAGCGATTCTTTATG |
| *PoxRsrA*_1080-1794_-VF | CTTTGGACGTGACTTTGAGG |
| *PoxRsrA*_1080-1794_-VR | CCATACTGCCAGGGTGC |
| *PoxRsrA*_891-1794_-LF | TCCCTTTGCCCGTTCCC |
| *PoxRsrA*_891-1794_-LR | CGCGGAGATTAGATGTAGCTATATTTA TTTGTTCAGTTTAGCCTTGTACTT |
| *PoxRsrA*_891-1794_-NF | GAATCCCGAAGTCATGGCTCT |
| *PoxRsrA*_891-1794_-NR | CCAGTAGGAGCGATTCTTTATG |
| *PoxRsrA*_891-1794_-VF | ACGCTTTGATTGCTGACTTG |
| *PoxRsrA*_891-1794_-VR | CCATACTGCCAGGGTGC |
| *PoxRsrA*_827-1794_-LF | CCCTTCGTCCGGGTTTAA |
| *PoxRsrA*_827-1794_-LR | CGCGGAGATTAGATGTAGCTATATTTATTCCATCGCATCACTCGGGTC |
| *PoxRsrA*_827-1794_-NF | CTGCACCCGAGCGATTGT |
| *PoxRsrA*_827-1794_-NR | GCGTCTGCGTCATTCACATT |
| *PoxRsrA*_827-1794_-VF | CGCTTTGATTGCTGACTTG |
| *PoxRsrA*_827-1794_-VR | CCATACTGCCAGGGTGC |
| *PoxRsrA*_1-1084_-RF | CTTGATCAACCGTCACCTCGAATGCAACCGACTAGCTACTGG |
| *PoxRsrA*_1-1084_-RR | AAGGCTGAAGGGGGAGTGTA |
| *PoxRsrA*_1-1084_-NF | TGATGATCGGTACGACCATTA |
| *PoxRsrA*_1-1084_-NR | CGACTGATGGGAACCATTCAC |
| *PoxRsrA*_1-1084_-VF | ATGCTACGACACCATCTGAC |
| *PoxRsrA*_1-1084_-VR | TCAGAATCATTGGAATCACC |
| *PoxRsrA*_1-881_-RF | CTTGATCAACCGTCACCTCGAATGATCAAGTACAAGGCTAAACTG |
| *PoxRsrA*_1-881_-RR | GTATTGTTGGGGACCGCT |
| *PoxRsrA*_1-881_-NF | TGATGATCGGTACGACCATTA |
| *PoxRsrA*_1-881_-NR | CTGAGAAAACACGCTGGAATA |
| *PoxRsrA*_1-881_-VF | GGTTCCTGCCTTCGGTTCA |
| *PoxRsrA*_1-881_-VR | TTGCCCCATTTCTTCGGATA |
| *PoxRsrA*_1-831_-RF | CTTGATCAACCGTCACCTCGAATGCCACCGCCAAATGA |
| *PoxRsrA*_1-831_-RR | CGTATTGTTGGGGACCGCT |
| *PoxRsrA*_1-831_-NF | TGATGATCGGTACGACCATTA |
| *PoxRsrA*_1-831_-NR | CTGAGAAAACACGCTGGAATAAC |
| *PoxRsrA*_1-831_-VF | GCTACGACACCATCTGACAA |
| *PoxRsrA*_1-831_-VR | ATCACTCGGGTCAACGGTAT |
| *PoxRsrA*_1-703_-RF | CTTGATCAACCGTCACCTCGAATGTACATGTCCCATTACAAGCGAT |
| *PoxRsrA*_1-703_-RR | ATCTTTTCCTGGGCTGTGGG |
| *PoxRsrA*_1-703_-NF | TTATGTTTCACAAGATAACGCAGAG |
| *PoxRsrA*_1-703_-NR | CGTGGGGTTGGTTCGGAG |
| *PoxRsrA*_1-703_-VF | ATGCTACGACACCATCTGACA |
| *PoxRsrA*_1-703_-VR | GATGCTGCTTGGTCACCTTGT |
| *PoxRsrA*_1-565_-RF | CTTGATCAACCGTCACCTCGAATGCCACCCTTGGAAGATCCCA |
| *PoxRsrA*_1-565_-RR | TTCGGAGACAGGCGTAGCAC |
| *PoxRsrA*_1-565_-NF | AAAGGCACAGTAGTTGTTGTTCG |
| *PoxRsrA*_1-565_-NR | GAACCGTTTGGGGTGATGTG |
| *PoxRsrA*_1-565_-VF | ATCCCTTTGCCCGTTCCCAC |
| *PoxRsrA*_1-565_-VR | CATCTCACGACGCTCTTCAT |
| *PoxRsrA*_1-467_-RF | CTTGATCAACCGTCACCTCGAATGAAGCCCCAGACTCAGCAA |
| *PoxRsrA*_1-467_-RR | TTCGGAGACAGGCGTAGCAC |
| *PoxRsrA*_1-467_-NF | TGACATGATGATCGGTACGA |
| *PoxRsrA*_1-467_-NR | GAACCGTTTGGGGTGATGTG |
| *PoxRsrA*_1-467_-VF | TTGAACGGAGAGACGAATGG |
| *PoxRsrA*_1-467_-VR | GCTGAGCGGGAAGAGGTGAG |
| *PoxRsrA*-LF | GTCAAACTCCTGTGCCGTAT |
| *PoxRsrA*-LR | GGTAATCCTTCTTTCTAGACCGGGCCACCCGATGAAG |
| *PoxRsrA*-RF | AATATCATCTTCTGTCGACGTTGGACGTGCGACATGAGC |
| *PoxRsrA*-RR | TGCCAGTTGTTGGAGGT |
| *tef1*P-F | AATATCATCTTCTGTCGACGCTCGTGGTTTGGCGGT |
| *tef1*P-R | TCGAGGTGACGGTTGATCAAG |
| *POX_c04083*Ter-F | ATATAGCTACATCTAATCTCCGCG |
| *POX_c04083*Ter-R | GGTAATCCTTCTTTCTAGACAGGAGCAAGTTCGTGGGT |
| *g418*-F | TCTAGAAAGAAGGATTACC |
| *g418*-R | GTCGACAGAAGATGATATT |
| *g418*-VR | GTGAATGCTCCGTAACACCCAAT |
| *g418*-VF | CGCTACTGCTTACAAGTGGGCTGAT |
| **Primers used for construction of mutants for investigation of subcellular localization of PoxRsrA** | |
| *PoxRsrA*orf-LF | CGAGTGATGCGATGGAAGTGT |
| *PoxRsrA*orf-LR | GGCTGAAGGGGGAGTGTA |
| *PoxRsrA*-RR | TGCCAGTTGTTGGAGGT |
| *PoxRsrA*-NF | CTTTGATTGCTGACTTGGGA |
| *PoxRsrA*-NR | GCGTCTGCGTCATTCACATT |
| *POX_c04083*ter-F | AGCTGTACAAGCACCACCACCACCACCACTAAATATAGCTACATCTAATCTCCGCG |
| *gfp*-F | TACACTCCCCCTTCAGCCGTGAGCAAGGGCG |
| *gfp*-R | TAGCTATATTTAGTGGTGGTGGTGGTGGTGCTTGTACAGCTCGTCCAT |
| *g418*-F | TCTAGAAAGAAGGATTACC |
| *g418*-R | GTCGACAGAAGATGATATT |
| *g418*-VR | GTGAATGCTCCGTAACACCCAAT |
| *g418*-VF | CGCTACTGCTTACAAGTGGGCTGAT |
| **Primers used for construction of *PoxRsrA* overexpression *Penicillium oxalicum* strains** | |
| *PoxRsrA*-F | ATGGACCCCCTTCGAGTG |
| *PoxRsrA*Ter-R | CGTGCAACTTGGTGATCTACATACCGTGCTGGTTTTCTCTGTG |
| *POX_d05452*-LF | GGCACCAAACACCGCTACA |
| *POX_d05452*-LR | TTTAGAGGTAATCCTTCTTTCTAGAGATGGACGAGACGGTACGATT |
| *POX_d05452*-RF | ATGTAGATCACCAAGTTGCA |
| *POX_d05452*-RR | TTCGGACGGTGTCACTTCG |
| *POX_d05452*-NF | AATGAGCGTAGTGTGAGAGCACCAAT |
| *POX_d05452*-NR | GTCGCACATCCAATCGCATAGAGT |
| *POX_d05452*-VF | CGCCTACGGAAAATGACG |
| *POX_d05452*-VR | ATGTCGCCGAAGATGGAG |
| *tef1*P-F | AATATCATCTTCTGTCGACGCTCGTGGTTTGGCGGT |
| *tef1*P-R | CACTCGAAGGGGGTCCATTCGAGGTGACGGTTGATCAAG |
| *g418*-F | TCTAGAAAGAAGGATTACC |
| *g418*-R | GTCGACAGAAGATGATATT |
| g418-VR | GTGAATGCTCCGTAACACCCAAT |
| g418-VF | CGCTACTGCTTACAAGTGGGCTGAT |
| **Primers used for construction of mutants where the phosphorylated amnio acid residues were site-mutated** | |
| *PoxRsrA*P-F | TCTTACCGCTGTTGAGATCCAGAGGGCTCAGGTTCAATGGGT |
| *PoxRsrA*Ter-R | CGTGCAACTTGGTGATCTACATACCGTGCTGGTTTTCTCTGTG |
| *PoxRsrA*_Y1127F_-F | GAAAAACTTCTACCAGCGACGCCTTGACTCTG |
| *PoxRsrA*_Y1127F_-R | CAGAGTCAAGGCGTCGCTGGTAGAAGTTTTTC |
| *PoxRsrA*_Y1170F_-F | TTGCTCCAAAACGTCGTTTCGAAGCTACCCCATCGGCCATCAT |
| *PoxRsrA*_Y1170F_-R | ATGATGGCCGATGGGGTAGCTTCGAAACGACGTTTTGGAGCAA |
| *PoxRsrA*-VF | TGCGAAAGTCGTTCAGCAGGAT |
| **Primers used for construction of *Penicillium oxalicum* deletion mutants** | |
| *PoxMed8*-LF | GTCTGCCACAAGGAGTCTACC |
| *PoxMed8*-LR | GGTAATCCTTCTTTCTAGAAGCCGAGCCTGTCCGAATAA |
| *PoxMed8*-RF | GCGAGTCCCCCTTTCCTTCTCCACGATGGCTCCCCTCACCTCGG |
| *PoxMed8*-RR | GACGAGCCGACGGTTTCATT |
| *PoxMed8*-NF | AGATTGCGTCACTTGCCTGTT |
| *PoxMed8*-NR | TGCCAGTGCTGTGGTCAAGG |
| *PoxMed8*-VF | GGATTGGTGGTTTGAGGGAT |
| *PoxMed8*-VR | CTCGCTATGCTTGACGATGATGTG |
| *PoxMed16*-LF | GAGTGTACAGGCGCTTCAGC |
| *PoxMed16*-LR | GGTAATCCTTCTTTCTAGACACGAATCTCACATTTGGGTC |
| *PoxMed16*-RF | GCGAGTCCCCCTTTCCTTCTCCACGATGCCCCTGATCATGGATGA |
| *PoxMed16*-RR | CCCTGATAGGCGTGTTTGA |
| *PoxMed16*-NF | GCGATGATGGCGTCAGGTA |
| *PoxMed16*-NR | CCAGGAATGTTGGTTGGTTTT |
| *PoxMed16*-VF | ATGGTCCGAAGGATTGTTTG |
| *PoxMed16*-VR | GCAGTTTGGCGGATGTTTT |
| *PoxMed31*-LF | TGTCAAGCAGCAGTGCGATG |
| *PoxMed31*-LR | GGTAATCCTTCTTTCTAGAGCTTGCGATAGACGTGTATATGT |
| *PoxMed31*-RF | AATATCATCTTCTGTCGACTCCATTGTTGGACGCAGACA |
| *PoxMed31*-RR | CGGCTGGTCTGTCTCTGGAT |
| *PoxMed31*-NF | CCAGAGTCTTGGTCACGATG |
| *PoxMed31*-NR | GCCTGGCTTTCAATGTTCCT |
| *PoxMed31*-VF | GAATCTACGCTTCACGCTCG |
| *PoxMed31*-VR | CTTCTCCCTTGTCCTCGCTT |
| *PoxMed6*-LF | TGACTGTCTCCCTTCATACTG |
| *PoxMed6*-LR | GGTAATCCTTCTTTCTAGAAGTGGCAAGATCAGAGAATTCG |
| *PoxMed6*-RF | AATATCATCTTCTGTCGACTTTGCTCGATTTAGATGATACC |
| *PoxMed6*-RR | TGCCACTTCTGGAGGCTTTG |
| *PoxMed6*-NF | TCCATTCGGTGGTGGGGTTC |
| *PoxMed6*-NR | CGGATAACGAAACGAGGGTC |
| *PoxMed6*-VF | AGTAGGTGAAATCCCCCGAA |
| *PoxMed6*-VR | GAGAGTTTTCCAGGCGTGTC |
| *POX_a01211*P-F | AGTTCTACGACGCACCAGCCAA |
| *POX_a01211*P-R | CGTGGAGAAGGAAAGGGGGACTCGC |
| *g418*-F | TCTAGAAAGAAGGATTACC |
| *g418*-R | GTCGACAGAAGATGATATT |
| g418-VR | GTGAATGCTCCGTAACACCCAAT |
| g418-VF | CGCTACTGCTTACAAGTGGGCTGAT |
| *PoxRsrA*_R866A_-F | TGCAGAATCTCTGCCTGGTGCAGATTTCAGACAGTGCATC |
| *PoxRsrA*_R866A_-R | GATGCACTGTCTGAAATCTGCACCAGGCAGAGATTCTGCA |
| *PoxRsrA*_R866K_-F | TGCAGAATCTCTGCCTGGTAAGGATTTCAGACAGTGCATC |
| *PoxRsrA*_R866K_-R | GATGCACTGTCTGAAATCCTTACCAGGCAGAGATTCTGCA |
| *PoxRsrA*P-F | TCTTACCGCTGTTGAGATCCAGAGGGCTCAGGTTCAATGGGT |
| *PoxRsrA*Ter-R | CGTGCAACTTGGTGATCTACATACCGTGCTGGTTTTCTCTGTG |
| **Primers used for construction of *Penicillium oxalicum* mutant PoxRsrA::GFP** | |
| *PoxRsrA-*F | CGAGTGATGCGATGGAAGTG |
| *PoxRsrA-*R | GCGTGGTGGCTGGGACCCAG |
| *gfp*-F | CTGGGTCCCAGCCACCACGCATGGTGAGCAAGGGCG |
| *gfp*-R | TTACTTGTACAGCTCGTCCAT |
| *PoxRsrA-*LF | CTTTGATTGCTGACTTGGGA |
| *PoxRsrA-*LR | GCGTCTGCGTCATTCACATT |
| *PoxRsrA-*NF | CCTCGTATTGGATTCTTTACCG |
| *PoxRsrA-*NR | TGAAGCGGACGCCTCATAAC |
| *g418-*VF | CGCTACTGCTTACAAGTGGGCTGAT |
| *g418-*VR | GTGAATGCTCCGTAACACCCAAT |
| **Primers used for construction of *Penicillium oxalicum* mutant Ptef1-*PoxRsrA*** | |
| *PoxRsrA-*NF | GGTTCAATGGGTCCTGCTTC |
| *PoxRsrA-*NR | GGCATTTCAGCACGAAGAGAC |
| *PoxRsrA-*LF | GCTGAAAGAGCCCATGTAGG |
| *PoxRsrA-*LR | GGTAATCCTTCTTTCTAGACCGGGCCACCCGATGAAGCGGA |
| *PoxRsrA-*RR | GCAGTCGCCATTCTTGTTCG |
| *g418-*F | TCTAGAAAGAAGGATTACC |
| *g418-*VF | CGCTACTGCTTACAAGTGGGCTGAT |
| *g418-*VR | GTGAATGCTCCGTAACACCCAAT |
| **Primers used for heterologous expression of *PoxRsrA*** | |
| *PoxRsrA*_830-883+1080-1140_-F1 | CATGGCTGATATCGGATCCGAATTCATGTTTCTGCCACCGCCAAATGA |
| *PoxRsrA*_830-883+1080-1140_-R1 | GCTGCCGCTACCGCCGCTACCGCCGCTACCGCCCTTGATCTCTTCCTTTGTCAGGTAA |
| *PoxRsrA*_830-883+1080-1140_-F2 | GGCGGTAGCGGCGGTAGCGGCGGTAGCGGCAGCGGGTATGGATCACTGCAACC |
| *PoxRsrA*_830-883+1080-1140_-R2 | GAGTGCGGCCGCAAGCTTGTCGACGTTACTCGAAATCTTTTTGTCCAGAGT |
| *PoxRsrA*_830-883_-F | CATGGCTGATATCGGATCCGAATTCATGTTTCTGCCACCGCCAAATGA |
| *PoxRsrA*_830-883_-R | GAGTGCGGCCGCAAGCTTGTCGACGTTACTTGATCTCTTCCTTTGTCAGGTAA |
| *PoxRsrA*_1080-1140_-F | CATGGCTGATATCGGATCCGAATTCGGGTATGGATCACTGCAACC |
| *PoxRsrA*_1080-1140_-R | GAGTGCGGCCGCAAGCTTGTCGACGTTACTCGAAATCTTTTTGTCCAGAGT |
| **Primers used for amplification of EMSA probes** | |
| *PoxGA15A*-F1 | ATGAAGGATCTCCAAGTGTAGC |
| *PoxGA15A*-F2 | FAM -ATGAAGGATCTCCAAGTGTAGC |
| *PoxGA15A*-R1 | AGTGATGAGCCTGGTAGAAGAA |
| *PoxGA15A*-R2 | FAM-AGTGATGAGCCTGGTAGAAGAA |
| *POX_b02418*-F | TGGGTCGCCTCAACATCTA |
| *POX_b02418*-R | FAM-CTTGACCGAGCGAAAATCAGAA |
| *PoxAmy13A*-F | TCGGACCAACCCATAAGG |
| *PoxAmy13A*-R | FAM-TGCCTCCTGATGATACCACA |
| *β-tubulin*-F | ACCTCACTTGCTCCGCTCTG |
| *β-tubulin*-R | FAM-ACAAACTTCATAGATGGAGTGGACA |
| *PoxGA15A*_-873_-F | TTTGGGCTTGCCTATGTCCG |
| *PoxGA15A*_-683_-F | ACGGGCAAAAGACGGAGATT |
| *PoxGA15A*_-531_-F | GAGTCTTGGAGGGGGCGAAT |
| *PoxGA15A*_-530_-F | AGTCTTGGAGGGGGCGAAT |
| *PoxGA15A*_-529_-F: | GTCTTGGAGGGGGCGAAT |
| *PoxGA15A*_-528_-F: | TCTTGGAGGGGGCGAAT |
| *PoxGA15A*_-527_-F: | CTTGGAGGGGGCGAAT |
| *PoxGA15A*_-526_-F: | TTGGAGGGGGCGAAT |
| *PoxGA15A*_-521_-F | GGGGGCGAATTCGATCAGGCAC |
| *PoxGA15A*_-511_-F | TCGATCAGGCACGCCCGTCCAT |
| *PoxGA15A*_-501_-F | ACGCCCGTCCATCTTCACACTA |
| *PoxGA15A*_-491_-F | ATCTTCACACTATCGGCTAGGT |
| *PoxGA15A*_-481_-F | TATCGGCTAGGTTCTTGCTA |
| *PoxGA15A*_-235_-F | GGAACCCCCCAGTAGAAAAC |
| *PoxGA15A*_-216_-R | GTTTTCTACTGGGGGGTTCC |
| *PoxGA15A*_-466_-R | AAGAACCTAGCCGATAGTGTGA |
| *PoxGA15A*_-477_-R | CGATAGTGTGAAGATGGACG |
| *PoxGA15A*_-487_-R | AAGATGGACGGGCGTGCCTGAT |
| *PoxGA15A*_-497_-R | GGCGTGCCTGATCGAATTCGCC |
| *PoxGA15A*_-507_-R | ATCGAATTCGCCCCCTCCAAGA |
| *PoxGA15A*_-517_-R | CCCCCTCCAAGACTCCTCGCAC |
| *PoxGA15A*_-518_-R | CCCCTCCAAGACTCCTCGCACG |
| *PoxGA15A*_-519_-R | CCCTCCAAGACTCCTCGCACG |
| *PoxGA15A*_-520_-R | CCTCCAAGACTCCTCGCACG |
| *PoxGA15A*_-521_-R | CTCCAAGACTCCTCGCACG |
| *PoxGA15A*_-522_-R | TCCAAGACTCCTCGCACG |
| *PoxGA15A*_-523_-R | CCAAGACTCCTCGCACGCC |
| *PoxGA15A*_-524_-R | CAAGACTCCTCGCACGCC |
| *PoxGA15A*_-525_-R | AAGACTCCTCGCACGCC |
| *PoxGA15A*_-526_R | AGACTCCTCGCACGCC |
| *PoxGA15A*_-527_-R | GACTCCTCGCACGCCCTG |
| *PoxGA15A*_-528_-R | ACTCCTCGCACGCCCTG |
| *PoxGA15A*_-529_-R | CTCCTCGCACGCCCTGGCC |
| *PoxGA15A*_-530_-R | TCCTCGCACGCCCTGGCC |
| *PoxGA15A*_-531_-R | CCTCGCACGCCCTGGCC |
| *PoxGA15A*_-532_-R | CTCGCACGCCCTGGCC |
| *PoxGA15A*_-661_-R | CAGAATCTCCGTCTTTTGCCC |
| *PoxGA15A*_-855_-R | GGACATAGGCAAGCCCAAATA |
| *PoxGA15A*_C-527A_-F | GGCGTGCGAGGAGTATTGGAGGGGGCGAATTC |
| *PoxGA15A*_C-527A_-R | GAATTCGCCCCCTCCAATACTCCTCGCACGCC |
| *PoxGA15A*_G-524A_-F | GGCGTGCGAGGAGTCTTAGAGGGGGCGAATTC |
| *PoxGA15A*_G-524A_-R | GAATTCGCCCCCTCTAAGACTCCTCGCACGCC |
| *PoxGA15A*_G-523T_-F | GGCGTGCGAGGAGTCTTGTAGGGGGCGAATTC |
| *PoxGA15A*_G-523T_-R | GAATTCGCCCCCTACAAGACTCCTCGCACGCC |
| **Primers used for yeast two-hybrid analysis** | |
| *PoxRsrA*_1_-F | CATGGAGGCCGAATTCATGGACCCCCTTCGAGTG |
| *PoxRsrA*_468_-F | CATGGAGGCCGAATTCATGAAGCCCCAGACTCAGCAA |
| *PoxRsrA*_1128_-F | CATGGAGGCCGAATTCATGTACCAGCGACGCCTTGACTCT |
| *PoxRsrA*_1434_-F | CATGGAGGCCGAATTCATGGCTAGTGAAGTTTCATCCAACT |
| *PoxRsrA*_1478_-F | CATGGAGGCCGAATTCATGCCTTATCAAGCGTCCTCCCG |
| *PoxRsrA*_1483_-F | CATGGAGGCCGAATTCATGTCCCGAGGATATCCTGATTA |
| *PoxRsrA*_1488_-F | CATGGAGGCCGAATTCATGGATTACAATATGAGCAATTAT |
| *PoxRsrA*_1493_-F | CATGGAGGCCGAATTCATGAATTATGGGCCGCCTGCCA |
| *PoxRsrA*_1498_-F | CATGGAGGCCGAATTCATGGCCAATGGCCCGGGAAATGC |
| *PoxRsrA*_1503_-F | CATGGAGGCCGAATTCATGAATGCTGTCAACAATGATTG |
| *PoxRsrA*_1504_-F | CATGGAGGCCGAATTCATGGCTGTCAACAATGATTGGAT |
| *PoxRsrA*_1506_-F | CATGGAGGCCGAATTCATGAACAATGATTGGATGGCTCG |
| *PoxRsrA*_1507_-F | CATGGAGGCCGAATTCATGAATGATTGGATGGCTCGCTT |
| *PoxRsrA*_1508_-F | CATGGAGGCCGAATTCATGGATTGGATGGCTCGCTTCGA |
| *PoxRsrA*_1509_-F | CATGGAGGCCGAATTCATGTGGATGGCTCGCTTCGATC |
| *PoxRsrA*_1510-_F | CATGGAGGCCGAATTCATGATGGCTCGCTTCGATCCGAG |
| *PoxRsrA*_1511_-F | CATGGAGGCCGAATTCATGGCTCGCTTCGATCCGAGAG |
| *PoxRsrA*_1512_-F | CATGGAGGCCGAATTCATGCGCTTCGATCCGAGAGCTCAA |
| *PoxRsrA*_1513_-F | CATGGAGGCCGAATTCATGTTCGATCCGAGAGCTCAACA |
| *PoxRsrA*_1514_-F | CATGGAGGCCGAATTCATGGATCCGAGAGCTCAACAGG |
| *PoxRsrA*_1515_-F | CATGGAGGCCGAATTCATGCCGAGAGCTCAACAGGGAGG |
| *PoxRsrA*_1516_-F | CATGGAGGCCGAATTCATGAGAGCTCAACAGGGAGGTCC |
| *PoxRsrA*_1517_-F | CATGGAGGCCGAATTCATGGCTCAACAGGGAGGTCCGC |
| *PoxRsrA*_1518_-F | CATGGAGGCCGAATTCATGCAACAGGGAGGTCCGCCTAC |
| *PoxRsrA*_1519_-F | CATGGAGGCCGAATTCATGCAGGGAGGTCCGCCTACCCA |
| *PoxRsrA*_1529_-F | CATGGAGGCCGAATTCATGCCTCCTCCAGCTCCATCGT |
| *PoxRsrA*_1539_-F | CATGGAGGCCGAATTCATGCGACCGGTCTCGTCAATGGC |
| *PoxRsrA*_1549_-F | CATGGAGGCCGAATTCATGTCCTACACGCAGTACGCGG |
| *PoxRsrA*_1559_-F | CATGGAGGCCGAATTCATGCAGCATGCTGCGCCGCCCTT |
| *PoxRsrA*_1569_-F | CATGGAGGCCGAATTCATGCCAGCCCCTTCCCCTGCG |
| *PoxRsrA*_1580_-F | CATGGAGGCCGAATTCATGGCCAGTTCACAGCGGCAAAG |
| *PoxRsrA*_1637_-F | CATGGAGGCCGAATTCATGGCCAATAGTCCGTTTGCTCT |
| *PoxRsrA*_1730_-R | GCAGGTCGACGGATCCTTAGGCTGAAGGGGGAGTGTA |
| *PoxRsrA*_1688_-R | GCAGGTCGACGGATCCTTATCGATGCGACTGATGGGAACC |
| *PoxRsrA*_1590_-R | GCAGGTCGACGGATCCTTACACGCTGGAATAACTTTGC |
| *PoxRsrA*_1569_-R | GCAGGTCGACGGATCCTTATGGTAACGAGCTCAAGGGCG |
| *PoxRsrA*_1559_-R | GCAGGTCGACGGATCCTTACTGAGATTGGCCACCCGCGT |
| *PoxRsrA*_1549_-R | GCAGGTCGACGGATCCTTAGGAGCCTTGGGTGGCCATTG |
| *PoxRsrA*_1539_-R | GCAGGTCGACGGATCCTTATCGACCCACTTGAGACGATG |
| *PoxRsrA*_1529_-R | GCAGGTCGACGGATCCTTAAGGTGGCTGGGACTGGGTAG |
| *PoxRsrA*_1518_-R | GCAGGTCGACGGATCCTTATTGAGCTCTCGGATCGAAG |
| *PoxRsrA*_1517_-R | GCAGGTCGACGGATCCTTAAGCTCTCGGATCGAAGCGA |
| *PoxRsrA*_1516_-R | GCAGGTCGACGGATCCTTATCTCGGATCGAAGCGAGCC |
| *PoxRsrA*_1515_-R | GCAGGTCGACGGATCCTTACGGATCGAAGCGAGCCATC |
| *PoxRsrA*_1514_-R | GCAGGTCGACGGATCCTTAATCGAAGCGAGCCATCCAAT |
| *PoxRsrA*_1513_-R | GCAGGTCGACGGATCCTTAGAAGCGAGCCATCCAATCATT |
| *PoxRsrA*_1512_-R | GCAGGTCGACGGATCCTTAGCGAGCCATCCAATCATTGTT |
| *PoxRsrA*_1511_-R | GCAGGTCGACGGATCCTTAAGCCATCCAATCATTGTTGA |
| *PoxRsrA*_1510_-R | GCAGGTCGACGGATCCTTACATCCAATCATTGTTGACAGC |
| *PoxRsrA*_1509_-R | GCAGGTCGACGGATCCTTACCAATCATTGTTGACAGCATTT |
| *PoxRsrA*_1508_-R | GCAGGTCGACGGATCCTTAATCATTGTTGACAGCATTTCC |
| *PoxRsrA*_1440_-R | GCAGGTCGACGGATCCTTAGTTGGATGAAACTTCACTAGC |
| *PoxRsrA*_1134_-R | GCAGGTCGACGGATCCTTAAGAGTCAAGGCGTCGCTGGTA |
| *PoxRsrA*_565_-R | GCAGGTCGACGGATCCTTACATTGGCATTTCAGCACGAAGAGA |
| *PoxRsrA*_467_-R | GCAGGTCGACGGATCCTTATGTCGCAGGCGGAGGACTG |
| *PoxRsrA*_D1508A_-F | ACAATGCATGGATGGCTCGCTTCGATCCGAGAGCTCAACA |
| *PoxRsrA*_D1508A_-R | GCGAGCCATCCATGCATTGTTGACAGCATTTCCCGGGCCA |
| *PoxRsrA*_W1509A_-F | ACAATGATGCAATGGCTCGCTTCGATCCGAGAGCTC |
| *PoxRsrA*_W1509A_-R | GCGAGCCATTGCATCATTGTTGACAGCATTTCCCG |
| *PoxRsrA*_M1510A_-F | CTGTCAACAATGATTGGGCAGCTCGCTTCGATCCGAGAGC |
| *PoxMed4*-F | CGCTCATATGGCCATGGAGGCCAGTATGAATTCGCTCATGCAATCTT |
| *PoxMed4*-R | TCCCGTATCGATGCCCACCCGGGTGCTAGAAGTCATCATCATCGGGGT |
| *PoxMed1*-F | CGCTCATATGGCCATGGAGGCCAGTATGGCTACTCCGTCAGCCAAACCTG |
| *PoxMed1*-R | TCCCGTATCGATGCCCACCCGGGTGTCAACCGCTGACGGCCCGCT |
| *PoxMed19*-F | CGCTCATATGGCCATGGAGGCCAGTATGTCTGATCGGACCCCTGCT |
| *PoxMed19*-R | TCCCGTATCGATGCCCACCCGGGTGTCATCTGGCCCCAATGCCAAAC |
| *PoxMed21*-F | CGCTCATATGGCCATGGAGGCCAGTATGGCGGACATTCTGACCCAG |
| *PoxMed21*-R | TCCCGTATCGATGCCCACCCGGGTGTCATTGCTCCCTGTCACCGTAAATT |
| *PoxMed13*-F | CGCTCATATGGCCATGGAGGCCAGTATGGACTTTCCCGGAGGCGC |
| *PoxMed13*-R | TCCCGTATCGATGCCCACCCGGGTGTCAAAAGATATAGCTGAGAAGCTCC |
| *PoxMed27*-F | CGCTCATATGGCCATGGAGGCCAGTATGAATGACGCATCAGCCAACGT |
| *PoxMed27*-R | TCCCGTATCGATGCCCACCCGGGTGTCAAGCGCAACTGGCGTGTAA |
| *PoxMed8*-F | CGCTCATATGGCCATGGAGGCCAGTATGGCTCCCCTCACCTCGG |
| *PoxMed8*-R | TCCCGTATCGATGCCCACCCGGGTGTCATGGCATATGACCCGTG |
| *PoxMed9*-F | CGCTCATATGGCCATGGAGGCCAGTATGGCTTCCAGATCCCCCAC |
| *PoxMed9*-R | TCCCGTATCGATGCCCACCCGGGTGTCATGGCCTGGTCGCCTCTC |
| *PoxMed16*-F | CGCTCATATGGCCATGGAGGCCAGTATGCCCCTGATCATGGATG |
| *PoxMed16*-R | TCCCGTATCGATGCCCACCCGGGTGTTATCCAGATCCTGCCACCTCC |
| *PoxMed18*-F | CGCTCATATGGCCATGGAGGCCAGTATGCACGAGCTCCTGCTCTT |
| *PoxMed18*-R | TCCCGTATCGATGCCCACCCGGGTGTCACTTGGCCCGCGTGTCCA |
| *PoxMed15*-F | CGCTCATATGGCCATGGAGGCCAGTATGAATCCCGCGGCGTTCTC |
| *PoxMed15*-R | TCCCGTATCGATGCCCACCCGGGTGTCATGCGCGGGAGGAGATGG |
| *PoxMed10*-F | CGCTCATATGGCCATGGAGGCCAGTATGGCCCCTGTAATGCTCAAG |
| *PoxMed10*-R | TCCCGTATCGATGCCCACCCGGGTGTCACTTGGATGCGCTCTCAG |
| *PoxMed11*-F | CGCTCATATGGCCATGGAGGCCAGTATGAGTCAAGAGTCACCAGCTCAAC |
| *PoxMed11*-R | TCCCGTATCGATGCCCACCCGGGTGTCAATCAACTTGCATCTCATCCG |
| *PoxMed7*-F | CGCTCATATGGCCATGGAGGCCAGTATGGCGGACGCAACCGAGCAAC |
| *PoxMed7*-R | TCCCGTATCGATGCCCACCCGGGTGTCAGTTGATCTTTTCCAAGAGG |
| *PoxMed14*-F | CGCTCATATGGCCATGGAGGCCAGTATGCCCGGTGTCATCATGGA |
| *PoxMed14*-R | TCCCGTATCGATGCCCACCCGGGTGTCAATCAATGGTGATGACATTGT |
| *PoxMed5*-F | CGCTCATATGGCCATGGAGGCCAGTATGCGGTCGGATATGTCATCAG |
| *PoxMed5*-R | TCCCGTATCGATGCCCACCCGGGTGCTAGAACATACCCTCCATATCCAAA |
| *PoxMed12*-F | CGCTCATATGGCCATGGAGGCCAGTATGATTCCTCATTCCTCCGC |
| *PoxMed12*-R | TCCCGTATCGATGCCCACCCGGGTGCTAAACACGCACACGTCTGG |
| *PoxMed31*-F | CGCTCATATGGCCATGGAGGCCAGTATGGAACCGGCGCAAGAC |
| *PoxMed31*-R | TCCCGTATCGATGCCCACCCGGGTGTCATGTGTGTGTCCCATTTCC |
| *PoxMed17*-F | CGCTCATATGGCCATGGAGGCCAGTATGGTCGATTCTTTCACCCTGCC |
| *PoxMed17*-R | TCCCGTATCGATGCCCACCCGGGTGTCATAGACGCGTCTCGTGCCTT |
| *PoxMed20*-F | CGCTCATATGGCCATGGAGGCCAGTATGCCACTCACAGGCGTCTACT |
| *PoxMed20*-R | TCCCGTATCGATGCCCACCCGGGTGCTATCGATTGAACCTCAGCACT |
| *PoxMed6*-F | CGCTCATATGGCCATGGAGGCCAGTATGGCAAGCCAGTCAGCCCCG |
| *PoxMed6*-R | TCCCGTATCGATGCCCACCCGGGTGTCAACTCACAACACGACCTTTTTTC |
| **Primers used for RT-qPCR assay** | |
| *Actin*-F | CTCCATCCAGGCCGTTCTG |
| *Actin*-R | CATGAGGTAGTCGGTCAAGTCAC |
| *PoxRsrA*-F | CGATCGACTCAAGGAGAAGAA |
| *PoxRsrA*-R | GCAGTCGCCATTCTTGTTCG |
| *PoxMed8*-F | ATGCCGAACTCACTGAACTCTG |
| *PoxMed8*-R | CCTCCATCTCATCTTCACCCTC |
| *PoxMed16*-F | CCAACCCACCCGTCTCAT |
| *PoxMed16*-R | CCTTGCGTTGCGACCATT |
| **Primers for construction of complementation strains** | |
| *POX_d05452*-LF | GGCACCAAACACCGCTACA |
| *POX_d05452*-LR | TTTAGAGGTAATCCTTCTTTCTAGAGATGGACGAGACGGTACGATT |
| *POX_d05452*-RF | ATGTAGATCACCAAGTTGCA |
| *POX_d05452*-RR | TTCGGACGGTGTCACTTCG |
| *POX_d05452*-NF | AATGAGCGTAGTGTGAGAGCACCAAT |
| *POX_d05452*-NR | GTCGCACATCCAATCGCATAGAGT |
| *POX_d05452*-VF | CGCCTACGGAAAATGACG |
| *POX_d05452*-VR | ATGTCGCCGAAGATGGAG |
| *ble*-F | TCTAGAAAGAAGGATTACCTC |
| *ble*-R | CTGGATCTCAACAGCGGTA |
| POX_d05452-LF | GGCACCAAACACCGCTACA |
| POX_d05452-LR | TTTAGAGGTAATCCTTCTTTCTAGAGATGGACGAGACGGTACGATT |
| POX_d05452-RF | ATGTAGATCACCAAGTTGCA |
| POX_d05452-RR | TTCGGACGGTGTCACTTCG |
| POX_d05452-NF | AATGAGCGTAGTGTGAGAGCACCAAT |
| POX_d05452-NR | GTCGCACATCCAATCGCATAGAGT |
| POX_d05452-VF | CGCCTACGGAAAATGACG |
| POX_d05452-VR | ATGTCGCCGAAGATGGAG |
| PoxMed31P-F | TCTTACCGCTGTTGAGATCCAGGCCACAGCACCATACATAACC |
| PoxMed31Ter-R | CGTGCAACTTGGTGATCTACATAGGACAGTTCGCTCGGGTTT |
| POX_d05452-LF | GGCACCAAACACCGCTACA |
| POX_d05452-LR | TTTAGAGGTAATCCTTCTTTCTAGAGATGGACGAGACGGTACGATT |
| POX_d05452-RF | ATGTAGATCACCAAGTTGCA |
| POX_d05452-RR | TTCGGACGGTGTCACTTCG |
| POX_d05452-NF | AATGAGCGTAGTGTGAGAGCACCAAT |
| POX_d05452-NR | GTCGCACATCCAATCGCATAGAGT |
| POX_d05452-VF | CGCCTACGGAAAATGACG |
| POX_d05452-VR | ATGTCGCCGAAGATGGAG |
| PoxMed6P-F | TCTTACCGCTGTTGAGATCCAGTAGGGTTTGGGATTGTGGCT |
| PoxMed6Ter-R | CGTGCAACTTGGTGATCTACATATCACCCACGGGATTGTAG |
| **Primers used for construction of Δ*SANT2*** | |
| SANT2-F | AAGTGGGTATGGATCACTGCAAGGACAAAAAGATTTCGAGGAG |
| SANT2-R | TTGCAGTGATCCATACCCACTT |
| *POX_d05452*-LF | GGCACCAAACACCGCTACA |
| *POX_d05452*-LR | TTTAGAGGTAATCCTTCTTTCTAGAGATGGACGAGACGGTACGATT |
| *POX_d05452*-RF | ATGTAGATCACCAAGTTGCA |
| *POX_d05452*-RR | TTCGGACGGTGTCACTTCG |
| *POX_d05452*-NF | AATGAGCGTAGTGTGAGAGCACCAAT |
| *POX_d05452*-NR | GTCGCACATCCAATCGCATAGAGT |
| *POX_d05452*-VF | CGCCTACGGAAAATGACG |
| *POX_d05452*-VR | ATGTCGCCGAAGATGGAG |
| *PoxRsrA*-VF | GTTCCTGCCTTCGGTTCA |
| *PoxRsrA*-VR | TCGCATCACTCGGGTCAA |
| *ble*-F | TCTAGAAAGAAGGATTACCTC |
| *ble*-R | CTGGATCTCAACAGCGGTA |
| SANT2-VF | GTTCCTGCCTTCGGTTCA |
| SANT2-VR | CAGAGTCAAGGCGTCGCTGGTAGAAGTTTTTC |
| **Primers used for copy number test** | |
| *g418*-F | CTTCCCGCTTCAGTGACAAC |
| *g418*-R | GGCTATGACTGGGCACAACA |
| *Actin*-F | CTCCATCCAGGCCGTTCTG |
| *Actin*-R | CATGAGGTAGTCGGTCAAGTCAC |
| *PoxRsrA*-LF | CCCCTTCAGATACCCACA |
| *PoxRsrA*-LR | GGAGAACCGAGCCAACGA |
| *PoxRsrA*-RF | CTGACGGTGATGATAACGAA |
| *PoxRsrA*-RR | CGCAACATTAACGCATACAA |
| *PoxRsrA*-F | CGATCGACTCAAGGAGAAGAA |
| *PoxRsrA*-R | GCAGTCGCCATTCTTGTTCG |
| *PoxMed6*-LF | ATTGAGAGGGGATGTTTACGC |
| *PoxMed6*-LR | TTGTATGATCGTAGGCGTATTTCT |
| *PoxMed6*-F | TCCCAGCCCAGTAAGGAGAA |
| *PoxMed6*-R | CACCAGCCCGTGATAGAATG |
| *PoxMed8*-F | ATGCCGAACTCACTGAACTCTG |
| *PoxMed8*-R | CCTCCATCTCATCTTCACCCTC |
| *PoxMed16*-F | CCAACCCACCCGTCTCAT |
| *PoxMed16*-R | CCTTGCGTTGCGACCATT |
| *PoxMed31*-LF | GCCACAGCACCATACATAACC |
| *PoxMed31*-LR | GAGCAGCGGCAACCTTCTAC |
| *PoxMed31*-F | CACAAGCATTTGCCGCCTAC |
| *PoxMed31*-R | GTCCTCGCTTTCGTCCTCCT |
| *POX_g08550*-LF | CAGGCATTCAACCCTCGTTT |
| *POX_g08550*-LR | GAGTCGGGAGTGGCTTCGTC |
| *POX_g08550*-F | GCTGTCGGAAGACTCGTCTGTT |
| *POX_g08550*-R | GCTCCACGGCAGTGATACA |
