# Supplementary material for: Regulation of fungal raw-starch-degrading enzyme production depends on transcription factor phosphorylation and recruitment of the Mediator complex
Source: Commun Biol. 2023 Oct 12;6:1032. doi: 10.1038/s42003-023-05404-x (PMC10570388; doi:10.1038/s42003-023-05404-x)

**Supplementary Data**

**Regulation of fungal raw-starch-degrading enzyme production depends on transcription factor phosphorylation and recruitment of the Mediator complex**

Yuan-Ni Ning^1,2,3^, Di Tian^1,2,3^, Man-Li Tan^3^, Xue-Mei Luo^3^, Shuai Zhao^1,2,3^*, Jia-Xun Feng^1,2,3^*

^1^State Key Laboratory for Conservation and Utilization of Subtropical Agro-bioresources, Guangxi University, 100 Daxue Road, Nanning, Guangxi 530004, People’s Republic of China.

^2^Guangxi Research Center for Microbial and Enzyme Engineering Technology, Guangxi University, 100 Daxue Road, Nanning, Guangxi 530004, People’s Republic of China.

^3^College of Life Science and Technology, Guangxi University, 100 Daxue Road, Nanning, Guangxi 530004, People’s Republic of China.

**Content:**

**Supplementary Data 3** Uncropped and unedited blots/gels corresponding to Supplementary Figures 2, 5, S1, S5, S6, S7, S8, S9, S13, S17, S18, S19, S21, S26, S30

*Correspondence: jiaxunfeng@sohu.com; shuaizhao0227@gxu.edu.cn

Tel: +86-771-323-9401

Mailing address: College of Life Science and Technology, Guangxi University, 100 Daxue Road, Nanning, Guangxi 530004, People’s Republic of China

**Fig. 2**

**
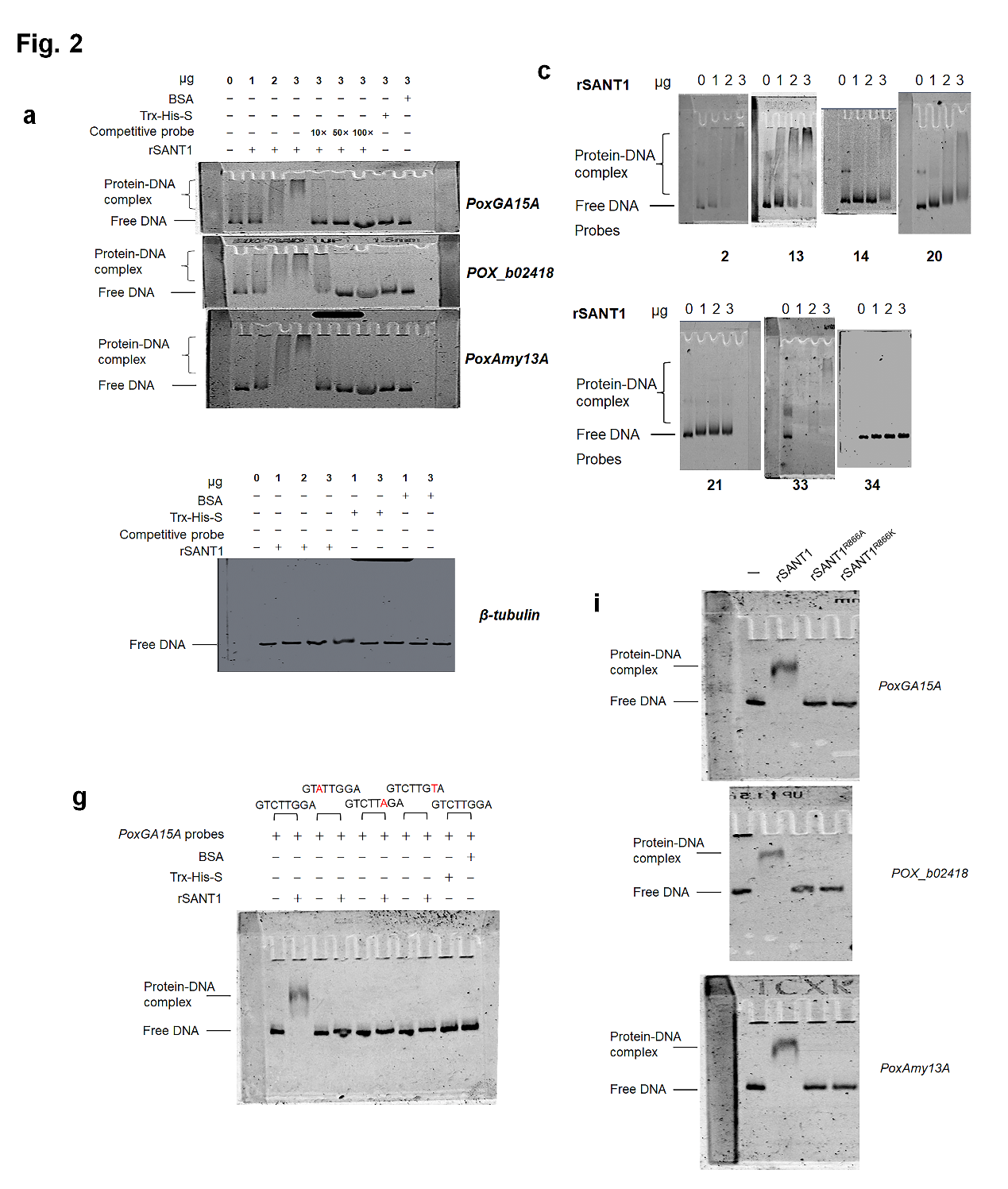
**

**Fig. 5**

**
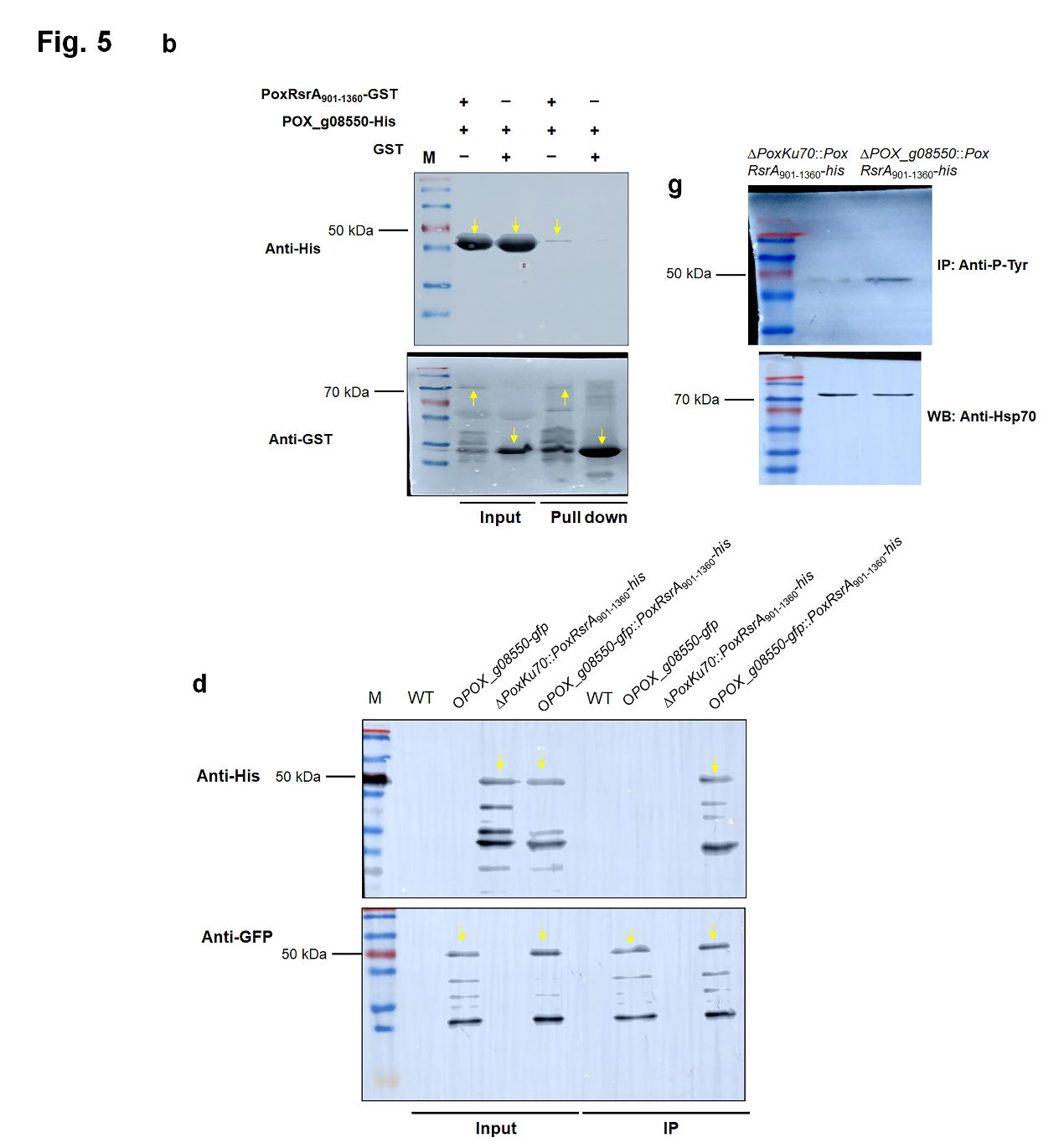
**

**Supplementary Fig. S1**

**
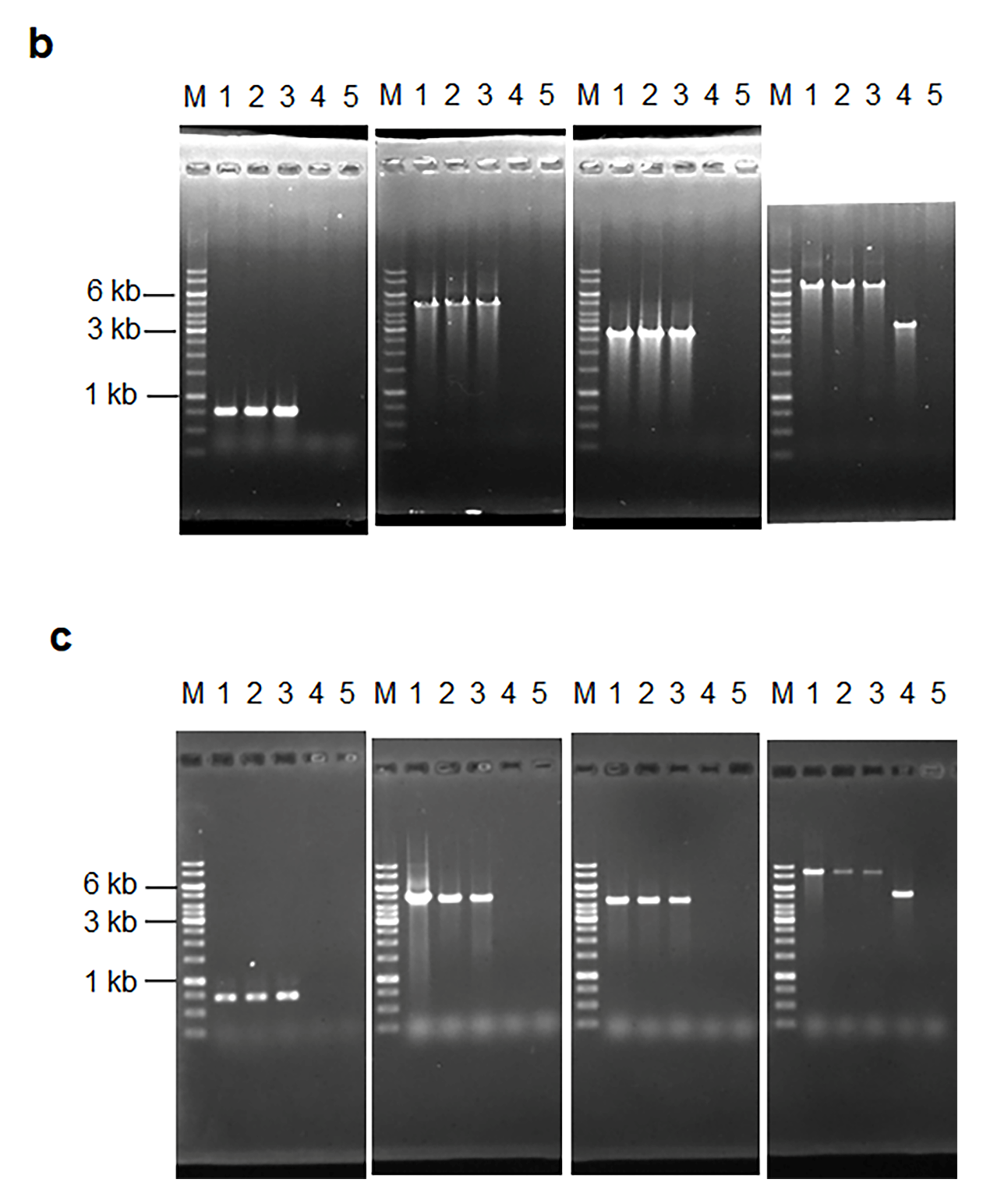
**

**Supplementary Fig. S5**


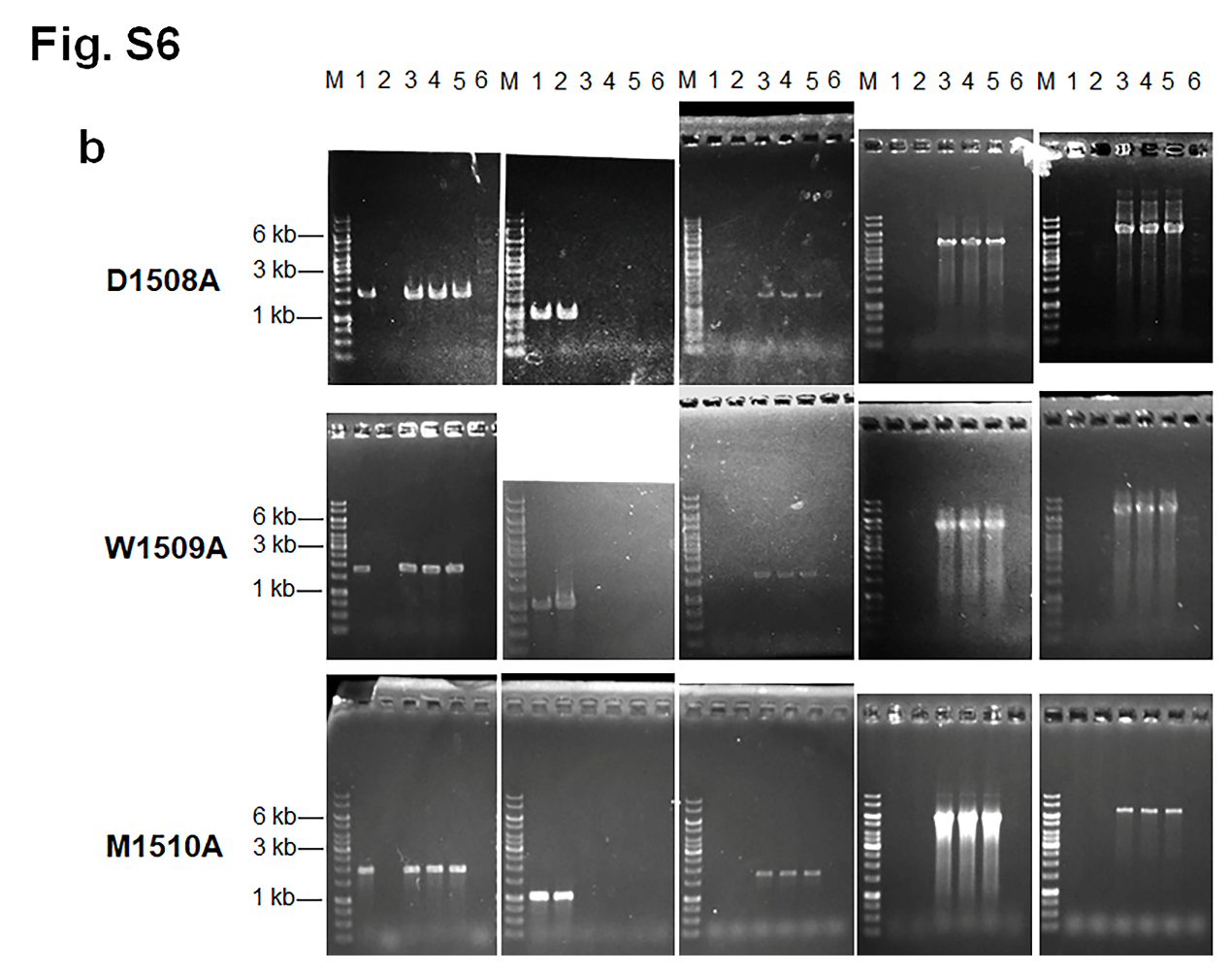


**Supplementary Fig. S6**


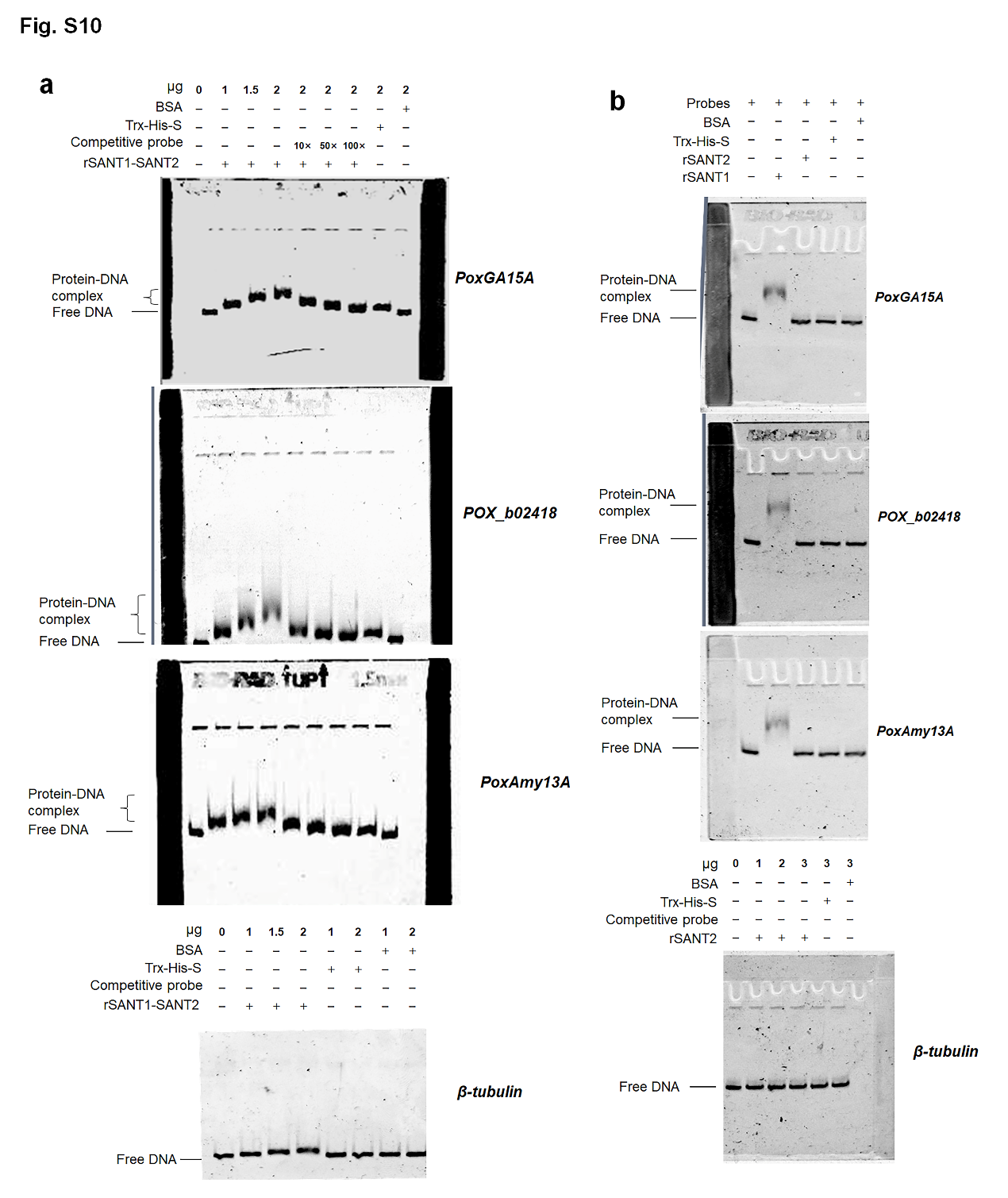


**Supplementary Fig. S7**


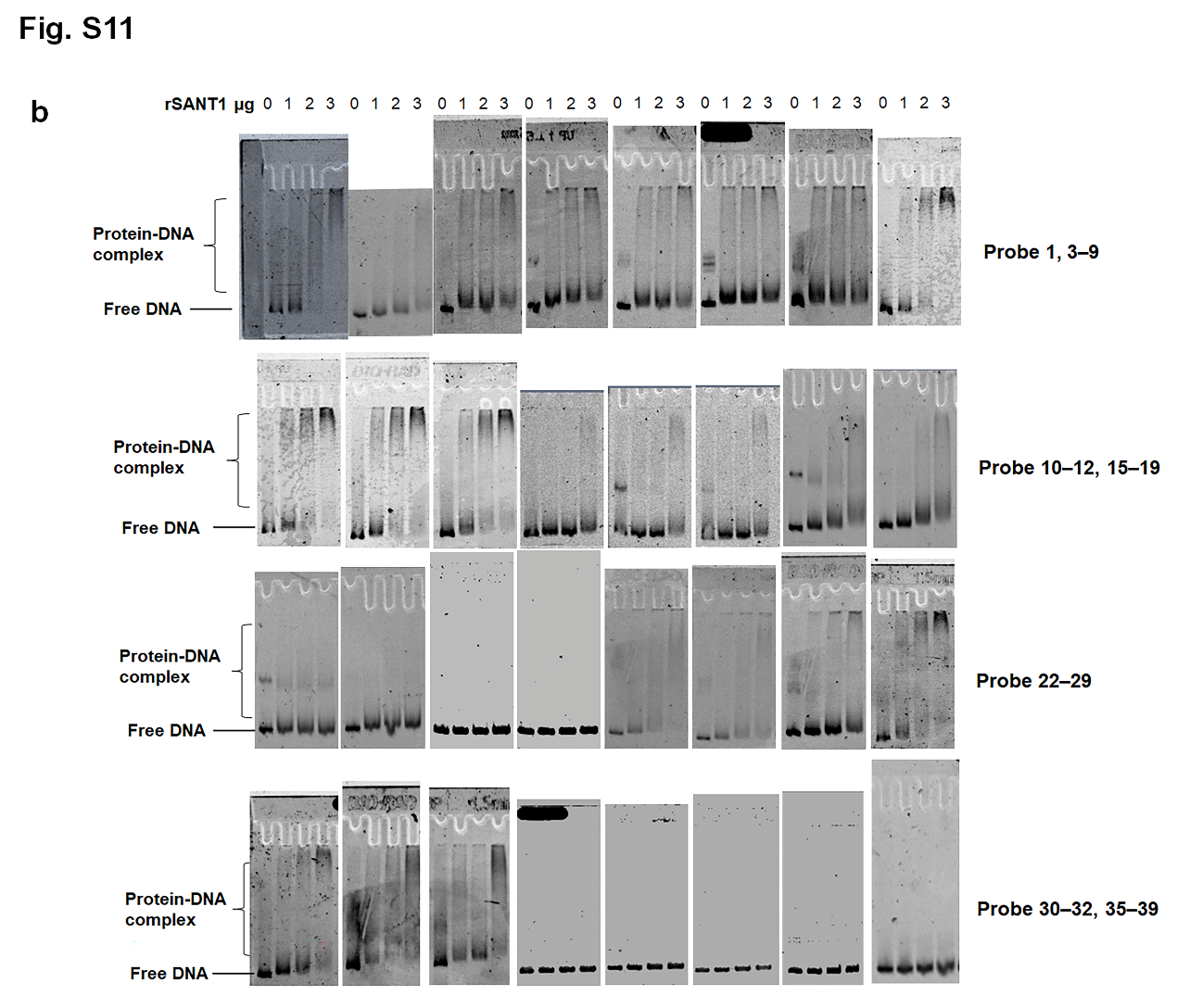


**Supplementary Fig. S8**


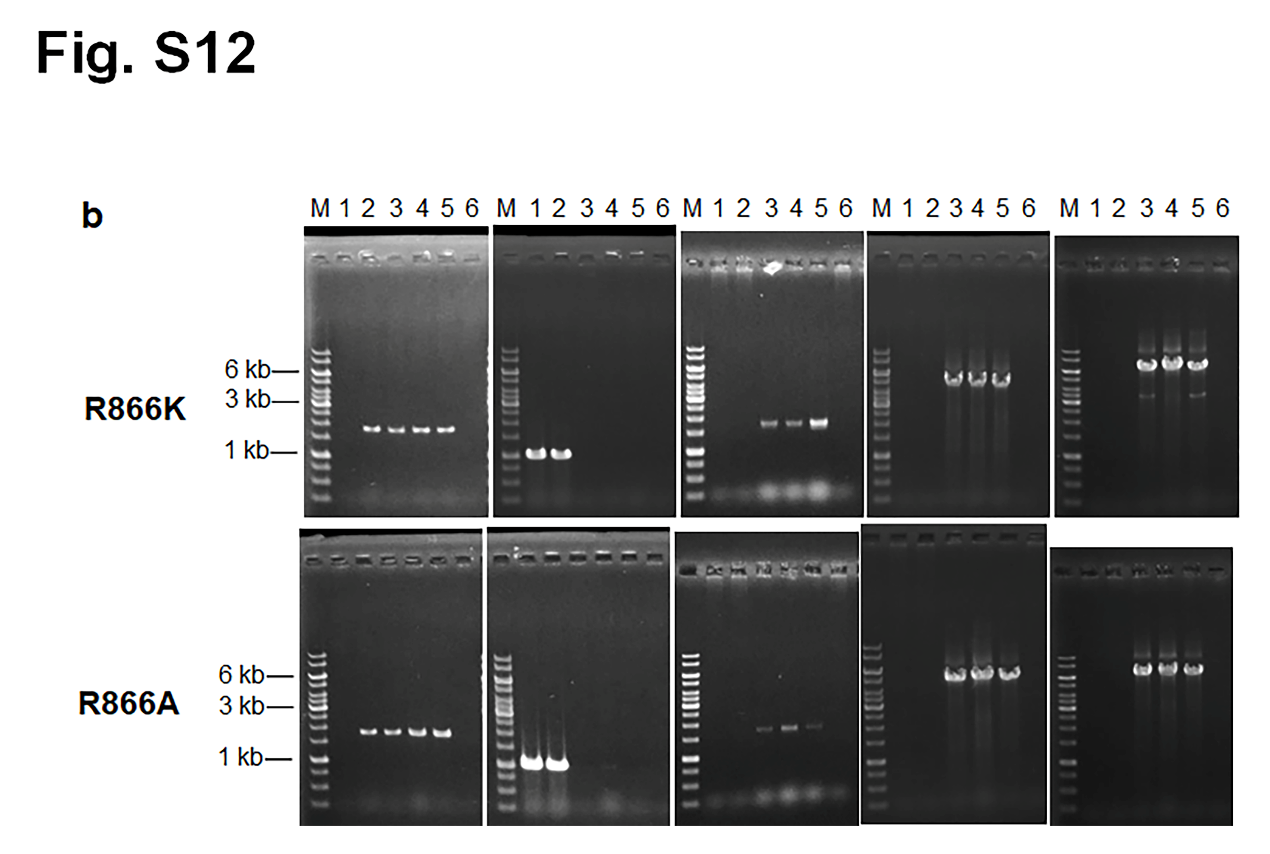


**Supplementary Fig. S9**


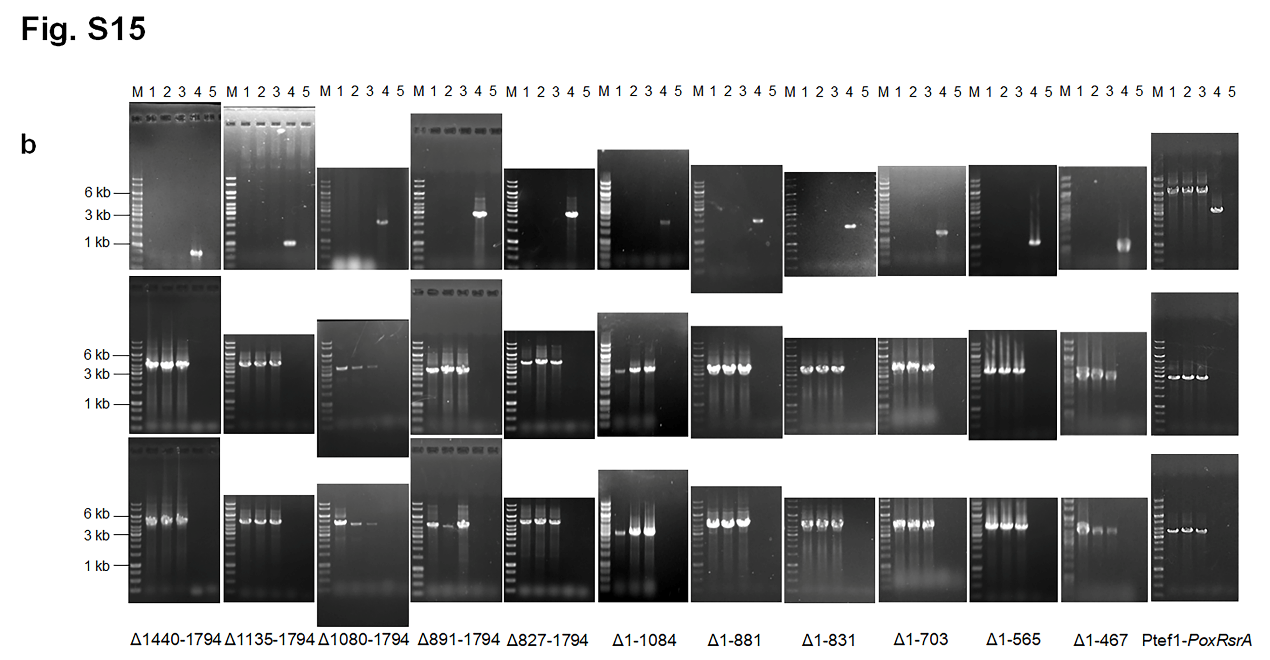


**Supplementary Fig. S13**


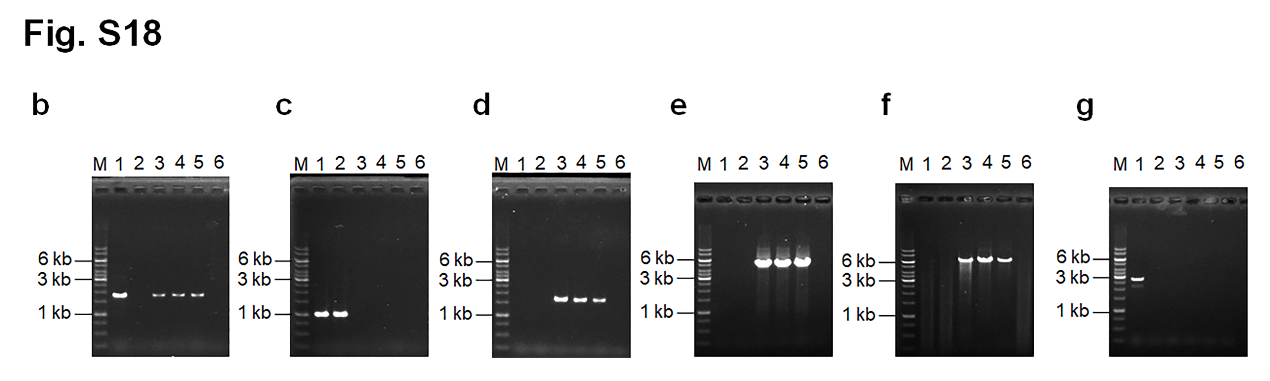


**Supplementary Fig. S17**


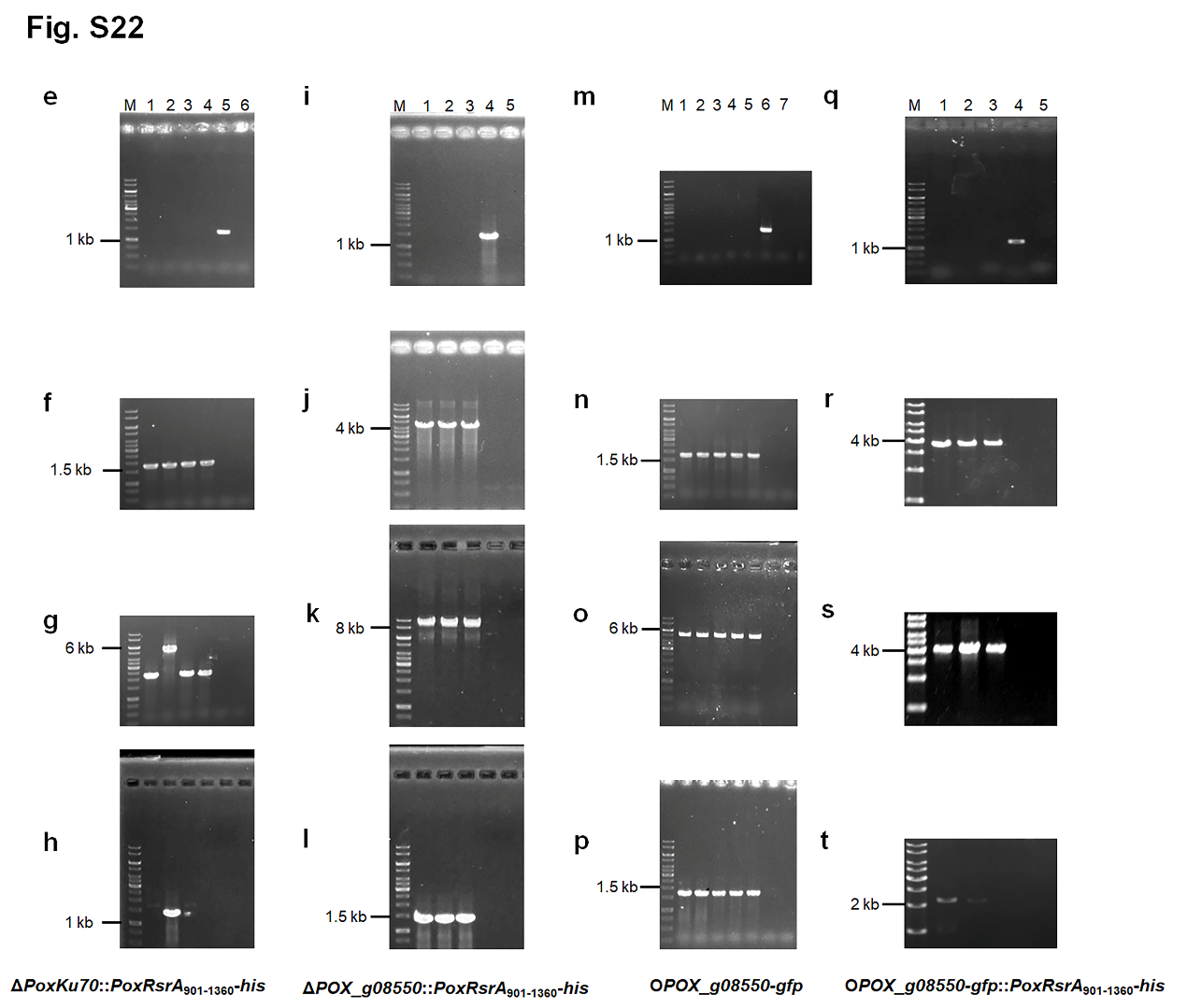


**Supplementary Fig. S18**


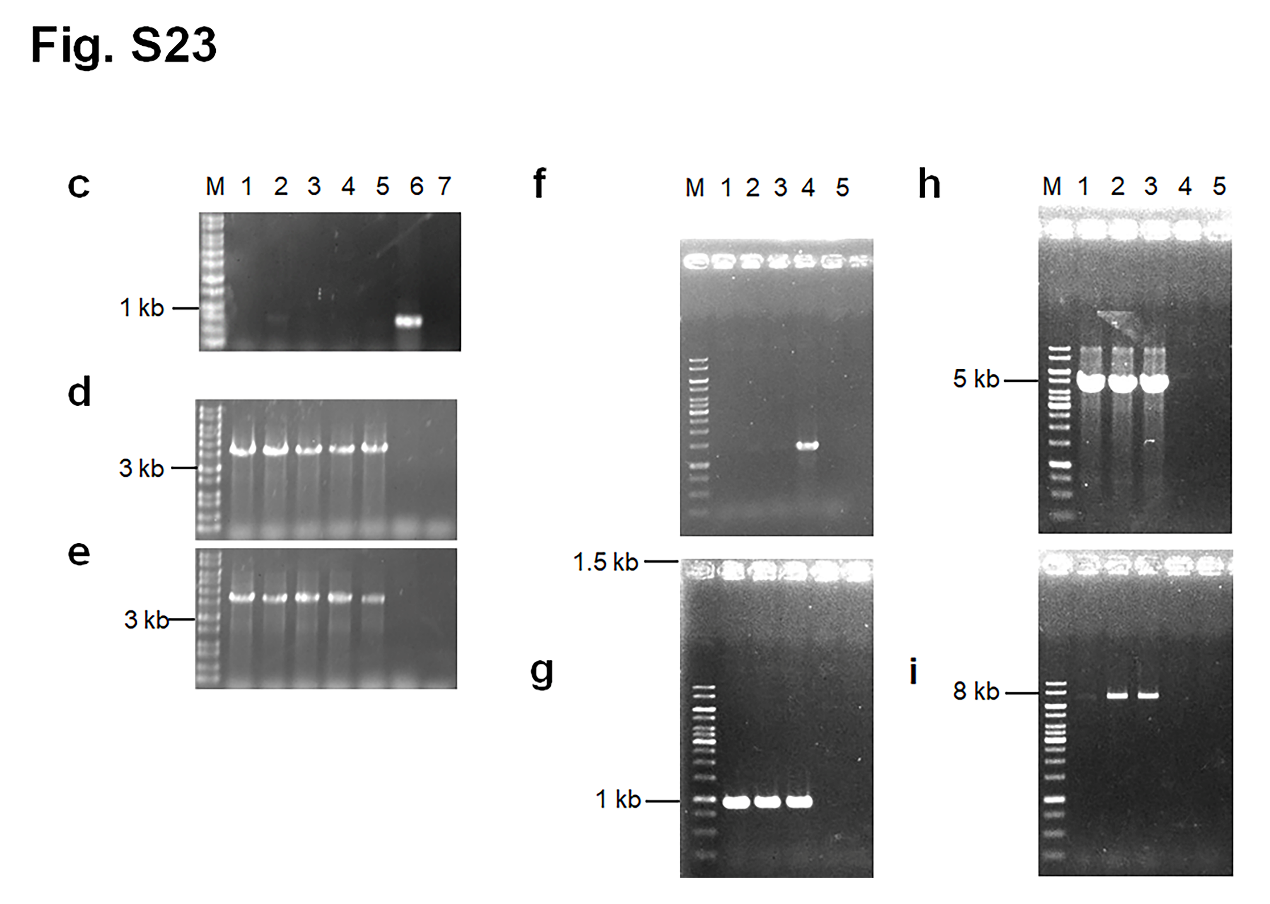


**Supplementary Fig. S19**


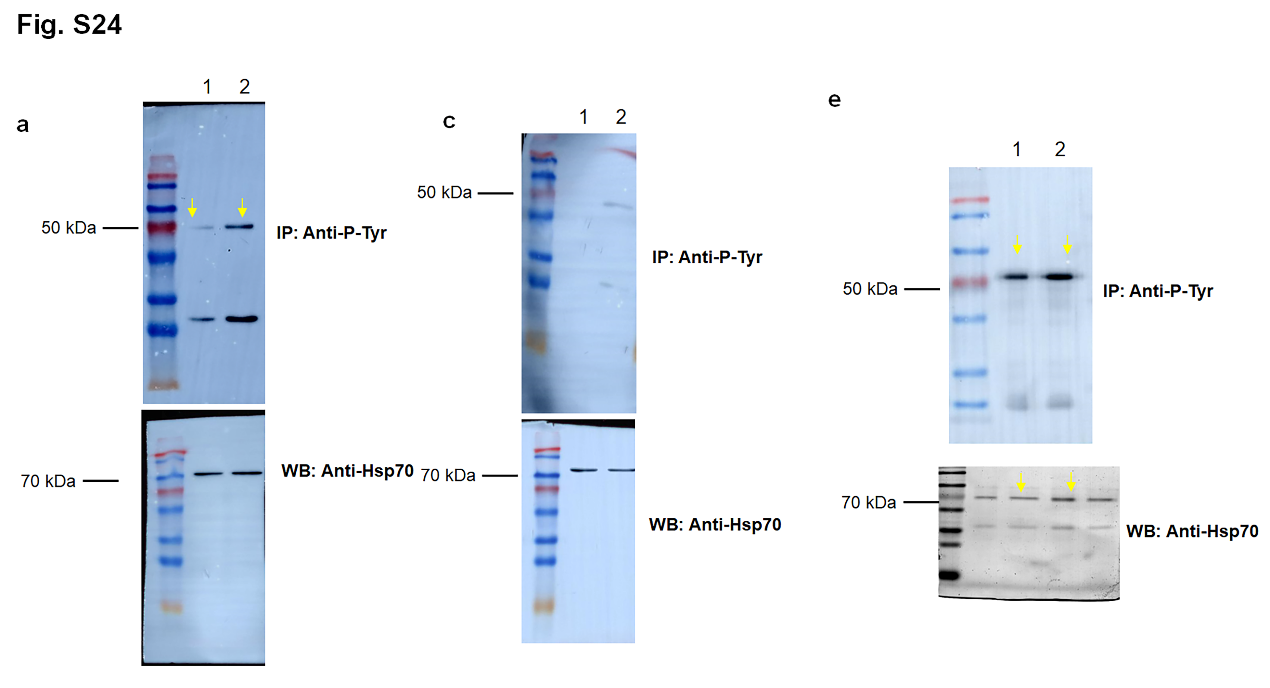


**Supplementary Fig. S21**


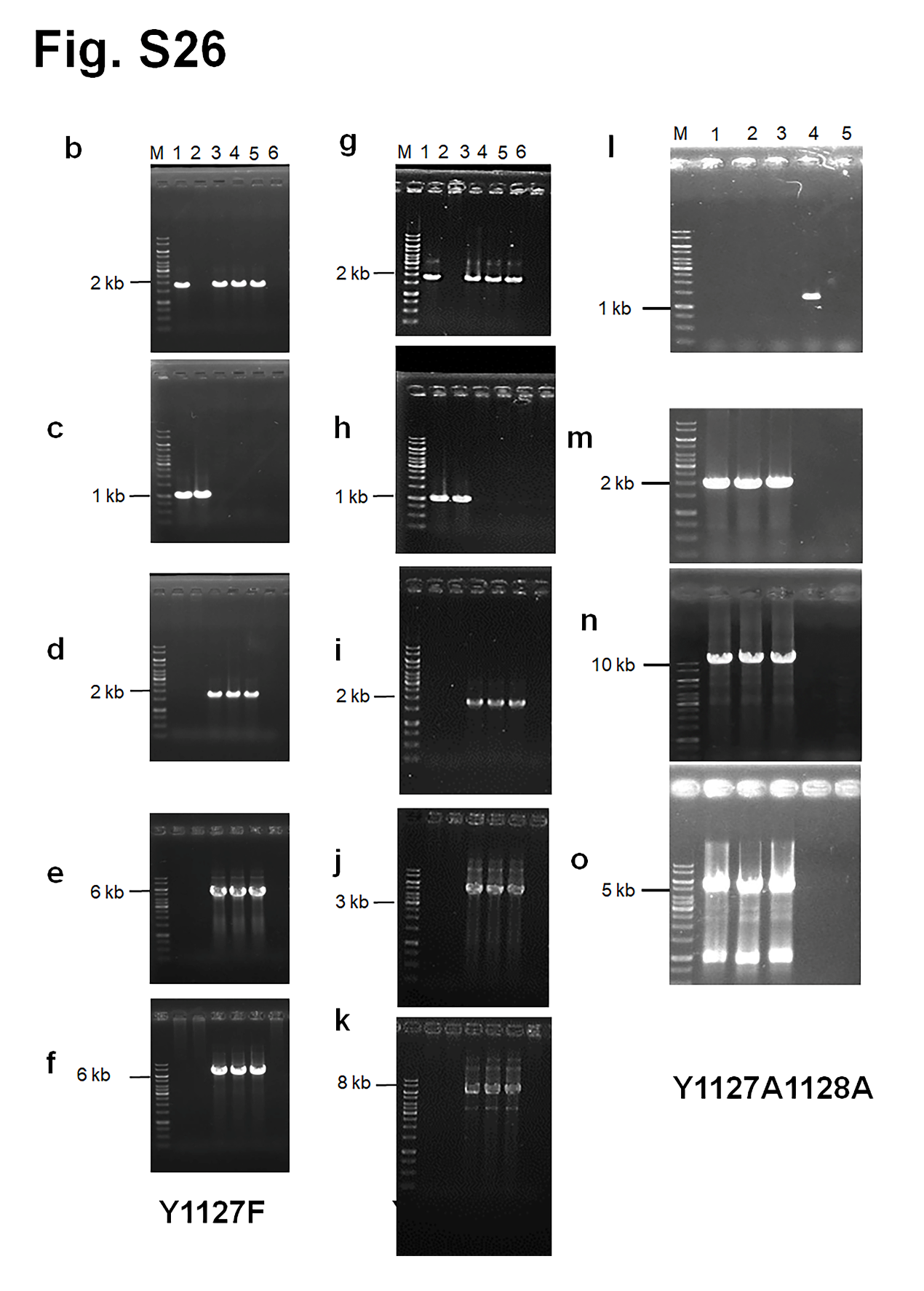


Y1170F

**Supplementary Fig. S26**


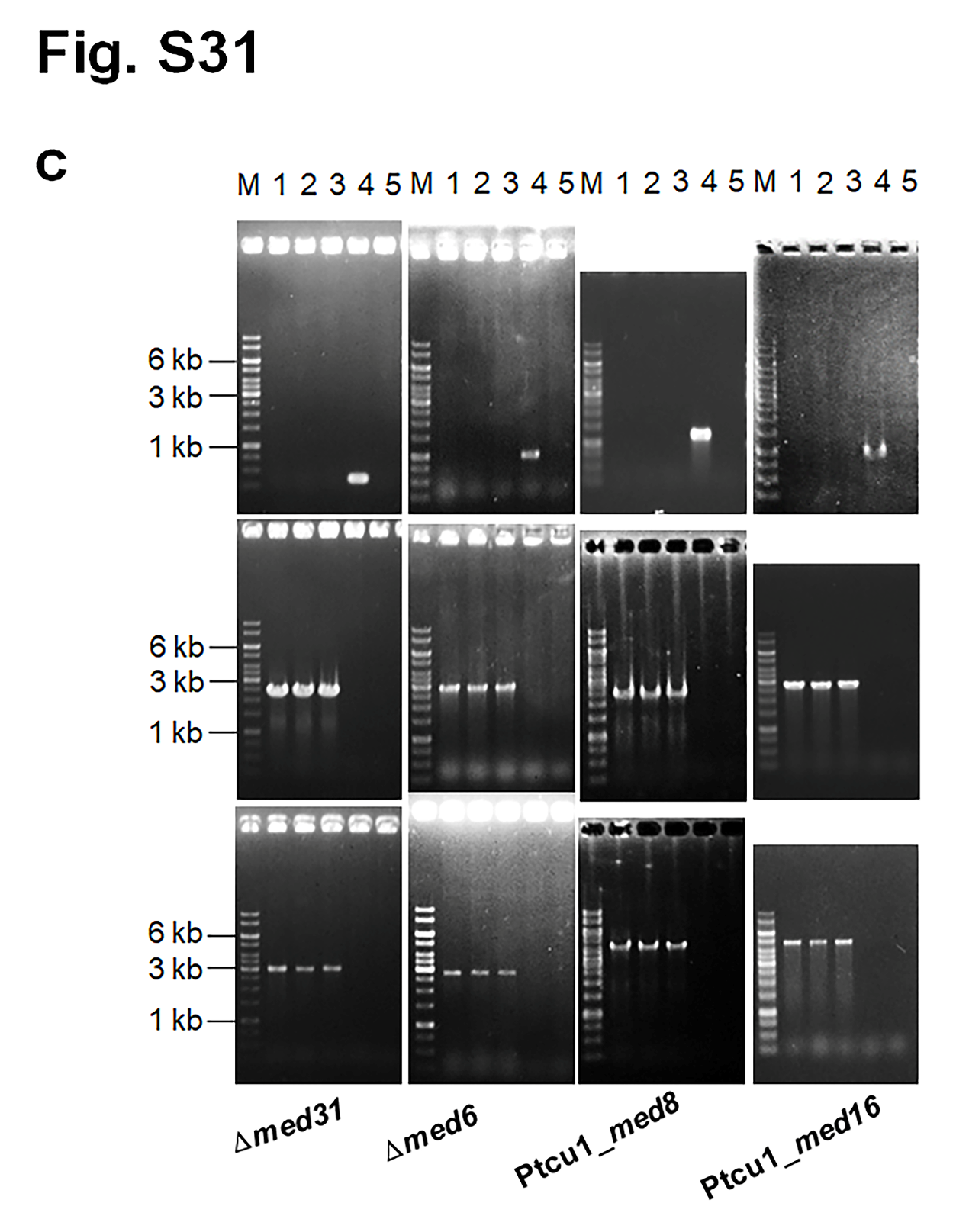


**Supplementary Fig. S30**


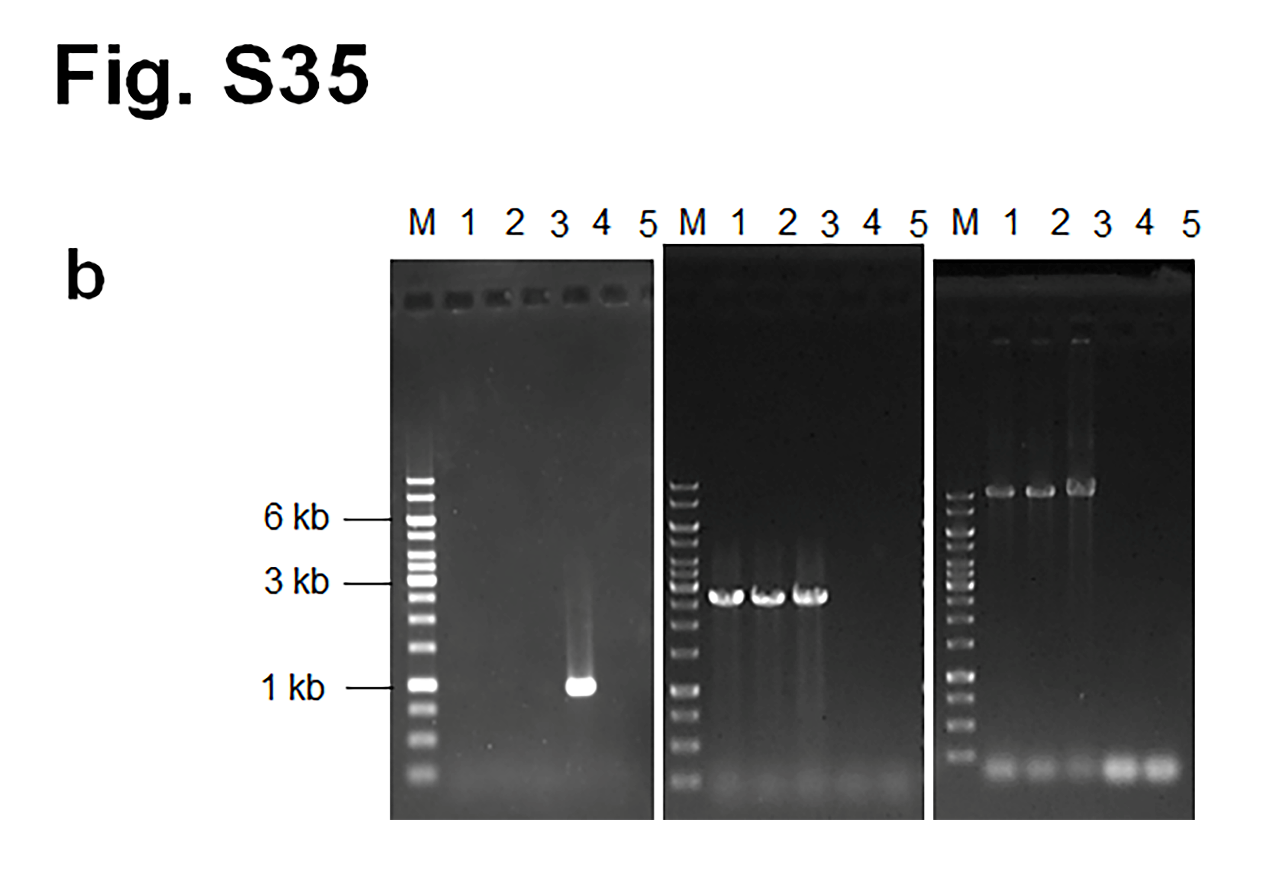

Supplement: Supplementary file 5 — Supplementary Data 3_4revised [file 42003_2023_5404_MOESM5_ESM.docx]
