# Supplementary material for: Regulation of fungal raw-starch-degrading enzyme production depends on transcription factor phosphorylation and recruitment of the Mediator complex
Source: Commun Biol. 2023 Oct 12;6:1032. doi: 10.1038/s42003-023-05404-x (PMC10570388; doi:10.1038/s42003-023-05404-x)
Supplement: Supplementary file 6 — Reporting summary [file 42003_2023_5404_MOESM6_ESM.pdf]

nature portfolio

Corresponding author(s): Jia-Xun Feng
Last updated by author(s): 9/2323

Reporting Summary

Nature Portfolio wishes to improve the reproducibility of the work that we publish. This form provides structure for consistency and transparency in reporting. For further information on Nature Portfolio policies, see our Editorial Policies and the Editorial Policy Checklist.

Statistics

For all statistical analyses, confirm that the following items are present in the figure legend, table legend, main text, or Methods section.

n/a	Confirmed	
☐	☒	The exact sample size (*n*) for each experimental group/condition, given as a discrete number and unit of measurement
☐	☒	A statement on whether measurements were taken from distinct samples or whether the same sample was measured repeatedly
☐	☒	The statistical test(s) used AND whether they are one- or two-sided<br>*Only common tests should be described solely by name; describe more complex techniques in the Methods section.*
☒	☐	A description of all covariates tested
☐	☒	A description of any assumptions or corrections, such as tests of normality and adjustment for multiple comparisons
☐	☒	A full description of the statistical parameters including central tendency (e.g. means) or other basic estimates (e.g. regression coefficient) AND variation (e.g. standard deviation) or associated estimates of uncertainty (e.g. confidence intervals)
☒	☐	For null hypothesis testing, the test statistic (e.g. *F*, *t*, *r*) with confidence intervals, effect sizes, degrees of freedom and *P* value noted<br>*Give P values as exact values whenever suitable.*
☒	☐	For Bayesian analysis, information on the choice of priors and Markov chain Monte Carlo settings
☒	☐	For hierarchical and complex designs, identification of the appropriate level for tests and full reporting of outcomes
☒	☐	Estimates of effect sizes (e.g. Cohen's *d*, Pearson's *r*), indicating how they were calculated

Our web collection on statistics for biologists contains articles on many of the points above.

Software and code

Policy information about availability of computer code

Data collection	No computer code
Data analysis	No computer code

For manuscripts utilizing custom algorithms or software that are central to the research but not yet described in published literature, software must be made available to editors and reviewers. We strongly encourage code deposition in a community repository (e.g. GitHub). See the Nature Portfolio guidelines for submitting code & software for further information.

Data

Policy information about availability of data

All manuscripts must include a data availability statement. This statement should provide the following information, where applicable:

- Accession codes, unique identifiers, or web links for publicly available datasets
- A description of any restrictions on data availability
- For clinical datasets or third party data, please ensure that the statement adheres to our policy

All data for gene sequences could be found in the submitted genome of P. oxalicum strain HP7-1 in GenBank (accession number JRVD02000000). Further inquiries can be directed to the corresponding authors, we can provide all rawdata.

Human research participants

Policy information about studies involving human research participants and Sex and Gender in Research.

Reporting on sex and gender	This information have not been collected. There is no report on sex and gender.
Population characteristics	See above
Recruitment	See above
Ethics oversight	See above

Note that full information on the approval of the study protocol must also be provided in the manuscript.

Field-specific reporting

Please select the one below that is the best fit for your research. If you are not sure, read the appropriate sections before making your selection.

☒ Life sciences ☐ Behavioural & social sciences ☐ Ecological, evolutionary & environmental sciences

For a reference copy of the document with all sections, see nature.com/documents/nr-reporting-summary-flat.pdf

Life sciences study design

All studies must disclose on these points even when the disclosure is negative.

Sample size	All experiments were performed at least three times
Data exclusions	The outlier was excluded
Replication	All experiment can be replicatied
Randomization	The samples were randomly selected for testing
Blinding	The group allocation during data collection were blinded

Reporting for specific materials, systems and methods

We require information from authors about some types of materials, experimental systems and methods used in many studies. Here, indicate whether each material, system or method listed is relevant to your study. If you are not sure if a list item applies to your research, read the appropriate section before selecting a response.

Materials & experimental systems

n/a	Involved in the study	
☐	☒	Antibodies
☒	☐	Eukaryotic cell lines
☒	☐	Palaeontology and archaeology
☒	☐	Animals and other organisms
☒	☐	Clinical data
☒	☐	Dual use research of concern

Methods

n/a	Involved in the study	
☒	☐	ChIP-seq
☒	☐	Flow cytometry
☒	☐	MRI-based neuroimaging

Antibodies

Antibodies used	Anti-P-Tyr: Phospho-Tyrosine Mouse mAb (P-Tyr-100), Cell signaling TECHNOLOGY, Product Number: 9411,lot Number:33, Product Type: Monoclonal Antibody, Species of Origin: Mouse;<br>Hsp70: HSP70(2D17) mAb for Plants, Abmart, Product Number: M20041S, lot Number:324184, Product Type: Monoclonal Antibody, Species of Origin: Mouse;<br>Anti-GFP: ProteinFind® Anti-GFP Mouse Monoclonal Antibody, TransGen Biotech, HT801-02, lot Number: Q20329, Product Type: Monoclonal Antibody, Species of Origin: Mouse;<br>Anti-GST: ProteinFind® Anti-GST Mouse Monoclonal Antibody, TransGen Biotech, HT601-02, lot Number: P20914, Product Type: Monoclonal Antibody, Species of Origin: Mouse;

Anti-His: ProteinFind® Anti-His Mouse Monoclonal Antibody, TransGen Biotech, HT501-02, lot Number:Q10801, Product Type: Monoclonal Antibody, Species of Origin: Mouse;
ProteinFind® Goat Anti-Mouse IgG (H+L), HRP Conjugate, TransGen Biotech, HS201-01 , lot Number:Q20329.

Validation

Anti-P-Tyr: Phospho-Tyrosine Mouse mAb (P-Tyr-100) is a high affinity antibody. ELISAs against a wide variety of phosphopeptides indicate that P-Tyr-100 binds phospho-Tyr in a manner largely independent of the surrounding amino acid sequence. 2D gel Western blot analysis of pervanadate-treated cell extracts also shows that P-Tyr-100 interacts with a broad range of tyrosine-phosphorylated proteins. P-Tyr-100 does not cross-react with peptides containing phospho-Ser or phospho-Thr. (U.S. Patent No's.: 6,441,140; 6,982,318; 7, 259, 022; 7, 344, 714; U.S.S.N. 11,484,485; and all foreign equivalents.), Monoclonal antibody is produced by immunizing animals with phospho-tyrosine containing peptides, Citations: 1. Mil Med Res. 2022 Oct 14;9(1):58. 2. Int J Mol Sci. 2022 Oct 14;23(20):12294. Development. 2022 Nov 1;149(21):dev200292.
Hsp70: TheHsp70 family is a set of highly conserved proteins that are indiced byavariety of biological stresses, including heat stress, in every organism in which the proteins have been examined.The humanHsp70 family members include: Hsp 70, a protein which is stronglyinducible in all organisms but which is also constitutively expressedin primate cells; Hsp 72, a 72 kDa protein that is induced exclusivelyunderstress conditions; Hsc 70, or cognate protein, is a72kDa.constitutively expressed, protein which is involved in the uncoating ofclathrin coated vesicles; GRP78, or BiP, is a glucose regulated78kDaprotein localized in the endoplasmic reticulum; and p75,orHsp75,a75kDa protein that is found within the mitochondria. Source: This Abmart monoclonal antibody is produced by immunizing mice with a polypeptide(Abmart SEAL mAb technology) corresponding to heat shock cognate 70 kDa protein. References: 1. Cell Mol Life Sci.62(6):670-684. 2. Cells Plant Physiol. 104(4):1429-37.
Anti-GFP: The Anti-GFP mouse monoclonal antibody is a highly purified anti mouse monoclonal antibody, which belongs to the same type of IgG1. The immunogen is a full-length synthetic GFP protein. The antibody can highly specifically recognize the GFP-tag at the C-terminal or N-terminal of the recombinant protein, and is applicable to qualitative or quantitative detection of GFP fusion protein. References: 1.Zhang J H , Sun T , Niu A , et al. Perturbation effect of reduced graphene oxide quantum dots (rGOQDs) on aryl hydrocarbon receptor (AhR) pathway in zebrafish[J]. Biomaterials, 2017, 133:49-59. 2.Chen Z H , Wang W T , Huang W , et al. The lncRNA HOTAIRM1 regulates the degradation of PML-RARA oncoprotein and myeloid cell differentiation by enhancing the autophagy pathway[J]. Cell Death and Differentiation, 2016. 3. Zhao L , Cheng D , Huang X , et al. A Light Harvesting Complex-Like Protein in Maintenance of Photosynthetic Components in Chlamydomonas[J]. Plant Physiology, 2017:pp.01465.2016. 4. Zhang J , Lai J , Wang F , et al. A SUMO Ligase AtMMS21 Regulates the Stability of the Chromatin Remodeler BRAHMA in Root Development[J]. Plant Physiology, 2017, 173(3):1574-1582.
Anti-GST: The Anti-GST labeled mouse monoclonal antibody is a highly purified mouse monoclonal antibody, which belongs to the same type of IgG2a. The immunogen is yeast Y258 GST recombinant protein. The antibody has high affinity with GST domain and is suitable for specific detection of GST fusion protein.
Anti-His: The Anti-His mouse monoclonal antibody is a highly purified mouse monoclonal antibody, which belongs to the same type of IgG1, and the immunogen is a synthetic 6 × His tag polypeptide sequence (HHHHHH). The antibody can highly specifically recognize 6 × His tag of the C-terminal or N-terminal of the recombinant protein is applicable to qualitative or quantitative detection of his fusion protein. References: 1. Zhang B , Yang Q , Chen J , et al. CRISPRi-Manipulation of Genetic Code Expansion via RF1 for Reassignment of Amber Codon in Bacteria[J]. Scientific Reports, 2016, 6:20000. 2. Li ST, Wang N, Xu S , et al. Quantitative study of yeast Alg1 beta-1, 4 mannosyltransferase activity, a key enzyme involved in protein N- glycosylation[J]. BBA - General Subjects, 2017, 1861(1):2934-2941.
Anti-Mouse IgG (H+L), HRP Conjugate: HRP (horseradish peroxidase) can catalyze ECL and other chemiluminescent reagents to produce chemiluminescence, or catalyze the color development of DAB, TMB and other substrates. HRP labeled goat anti mouse IgG (H+L) antibody is a highly purified mouse antibody, which can specifically recognize the heavy chain and light chain of mouse IgG, but has no significant cross reaction to other species of IgG. It has the characteristics of low background and high sensitivity, and is suitable for Western Blot, ELISA, etc. References: 1.Zhang B , Yang Q , Chen J , et al. CRISPRi-Manipulation of Genetic Code Expansion via RF1 for Reassignment of Amber Codon in Bacteria[J]. Scientific Reports, 2016, 6:20000. 2.Li ST, Wang N, Xu S , et al. Quantitative study of yeast Alg1 beta-1, 4 mannosyltransferase activity, a key enzyme involved in protein N- glycosylation[J]. BBA - General Subjects, 2017, 1861(1):2934-2941.
